# An Intelligent and Conductive Hydrogel with Multiresponsive and ROS Scavenging Properties for Infection Prevention and Anti‐Inflammatory Treatment Assisted by Electrical Stimulation for Diabetic Wound

**DOI:** 10.1002/advs.202500696

**Published:** 2025-05-08

**Authors:** Tao Zhang, Zongwu Meng, Haoyu Yu, Ping Ding, Tianhan Kai

**Affiliations:** ^1^ Xiangya School of Public Health Central South University Changsha Hunan 410013 China; ^2^ Furong Laboratory Central South University Changsha Hunan 410008 China; ^3^ Hunan Provincial Key Laboratory of Clinical Epidemiology Central South University Changsha Hunan 410078 China

**Keywords:** antibacterial, conductive hydrogel, diabetic wound, electrical stimulation, multiple responses, ROS scavenging, synergistic anti‐inflammatory

## Abstract

Diabetic wounds experience a hyperglycemic, hypoxic environment, combined with ongoing oxidative stress and inflammatory imbalances, significantly disrupts normal healing process. Advanced hydrogels have been considered one of the most exciting medical biomaterials for the potential in wounds healing. Herein, a novel conductive hydrogel (HEPP), designed to release nanozyme (PTPPG) in response to its microenvironment, was created to facilitate glucose (Glu) catabolism. Furthermore, the HEPP integrates photodynamic therapy (PDT), photothermal therapy (PTT), and self‐cascading reactive oxygen species (ROS) to prevent bacterial infections while ensuring a continuous supply of oxygen (O_2_) to the wound. The HEPP not only adeptly controls high ROS levels, but also enhances the regulation of inflammation in the wound area via electrical stimulation (ES), thereby promoting healing that is supported by the immune response. Studies conducted in vitro, along with transcriptomic analyses, indicate that ES primarily mitigates inflammation by regulating Interleukin‐6 (IL‐6) and tumor necrosis factor‐α (TNF‐α). The effects of HEPP combined with ES are primarily connected to their impact on TNF signaling pathways. By reducing the formation of ROS and employing ES to effectively lessen inflammation, this approach offers an innovative method to manage complicated diabetic wounds, ulcers, and a range of inflammatory conditions linked to infections.

## Graphical abstract

This study introduces a novel therapeutic approach that employs a conductive hydrogel (HEPP) responsive to near‐infrared light, pH levels, and reactive oxygen species. Its goal is to enhance the healing environment affected by elevated glucose levels, insufficient oxygen, and bacterial infections in diabetes‐related wounds. The analysis of gene sequencing, along with the experimental findings, revealed the mechanism of the synergistic anti‐inflammatory effects facilitated by this hydrogel assisted by electrical stimulation.

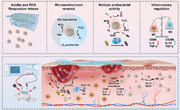

## 1 Introduction

Diabetes is a global metabolic disorder that impacts around 10% of the population worldwide, with an annual growth rate of roughly 1%.^[^ ^1^ ^]^ Hyperglycemia in diabetes leads to a variety of complications, posing a significant threat to public health.^[^ ^2^ ^]^ Diabetic wound ulcers are one of the common complications of diabetes, accompanied by extremely high mortality and amputation rates.^[^ ^3^ ^]^ In contrast to the typical wound healing process, which progresses through a continuous sequence of four distinct phases—hemostasis, inflammation, proliferation, and tissue remodeling, diabetic ulcers exhibit disrupted angiogenesis and a chronic inflammatory state.^[^ ^4^ ^]^ The slow recovery of diabetic ulcers is complicated by infections and low O_2_ levels in the surrounding area, primarily due to elevated blood glucose, increased oxidative stress, and heightened inflammation, which contribute to a weakened local immune response, ultimately hindering the healing of wounds.^[^ ^5^ ^]^ The rapid increase in the number of diabetic patients worldwide presents significant challenges for the clinical treatment of diabetic wound ulcers. The existing approaches to treating diabetic ulcers involves a combination of surgical debridement and antibiotic administration, as well as negative pressure wound treatment, among other techniques.^[^ ^6^ ^]^ However, traditional treatment regimens have obvious deficiencies in controlling infection, antibiotic resistance, and on‐demand treatment.^[^ ^7^ ^]^ Hence, the creation of innovative biomaterials and approaches to manage the microenvironment of diabetic ulcers presents significant promise to realize intelligent wound management.

The risk of bacterial infection inherently challenges the management of chronic wounds due to the disruption of the protective barrier of skin, which may lead to significant systemic inflammatory reactions, sepsis, and other serious issues.^[^ ^8^ ^]^ The escalating challenge of bacterial resistance to frequently utilized antibiotics, coupled with the development of biofilms that result in treatment failures, is increasingly alarming. On the other hand, another major culprit for the delayed healing of chronic diabetic wounds is the inflammatory response caused by wound injury.^[^ ^9^ ^]^ If the immune system fails to effectively remove the bacteria present in the wound within an appropriate timeframe, it risks transitioning into chronic inflammation. This condition is characterized by damaged blood vessels, which can cause a lack of O_2_, an increase in lactic acid, and eventually create an acidic and low‐oxygen environment at the wound site.^[^ ^10^ ^]^ Furthermore, high blood glucose not only increases the possible of wound infection but also leads to protein glycosylation and stimulates the generation of pro‐inflammatory cytokine, resulting in oxidative damage and delayed wound healing.^[^ ^11^ ^]^ The accumulation of ROS in chronic wounds exhibits two separate characteristics: simply lowering ROS to normal physiological levels does not suffice for antimicrobial action, while excessively high concentrations of ROS can result in oxidative stress and tissue damage.^[^ ^12^ ^]^ Consequently, an approach that facilitates the cleansing of wounds from bacteria and manages inflammation at the injury location is the most effective method for enhancing the healing process.

The use of near‐infrared (NIR) technology has increasingly gained popularity because of its benefits, including being noninvasive, offering high spatial resolution, and allowing for precise time management.^[^ ^13^ ^]^ By enhancing the concentration of ROS and elevating local temperatures, PDT and PTT effectively destroy bacterial structures, ultimately causing bacterial death and breaking down biofilms, thereby preventing the emergence of drug resistance.^[^ ^9^, ^14^ ^]^ Meanwhile, mild photothermal stimulation promotes the healing of diabetic wounds by accelerating drug absorption and enhancing endothelial cell function via improved blood circulation. These results suggest that the use of NIR therapy could be highly beneficial in treating wounds with bacterial complications. Still, relying solely on phototherapy has significant drawbacks when controlling inflammation in wound healing.^[^ ^9^, ^15^ ^]^ For instance, the penetration depth of NIR light is limited, and high energy density or long‐term NIR irradiation may cause local overheating and activate mitochondrial cytochrome C oxidase to produce ROS to aggravate the inflammatory response.^[^ ^16^ ^]^ On the other hand, ES reveals greater effectiveness in the field of biomedicine, owing to its therapeutic advantages in deeper tissues, the ability to modify parameters instantaneously, and its positive safety profile.^[^ ^17^ ^]^ Research indicates that utilizing ES in therapeutic practices can greatly enhance wound healing and offers innovative possibilities for wearable technology aimed at wound management.^[^ ^18^ ^]^ It not only facilitates swift movement and growth of cells, collagen formation, and the development of new blood vessels, but it also offers considerable benefits in managing inflammation.^[^ ^19^ ^]^ For example, Bao and co‐workers designed a low‐impedance adhesive hydrogel electrode based on poly (3,4‐ethylenedioxythiophene):polystyrene sulfonate to continuously monitor physiological signals, promote faster wound healing, enhance the formation of new blood vessels, and support skin recovery.^[^ ^20^ ^]^ Another instance reveals that a bioelectronic patch featuring a double‐network MXene (Ti_3_C_2_)‐based hydrogel can function as an air cathode for magnesium batteries, maintaining a reliable current output and utilizing its unique electrochemical properties to control inflammation, thereby promoting faster wound healing.^[^ ^21^ ^]^ Hence, by taking full advantage of the merits of both therapies, the integration of infrared therapy with ES presents considerable potential for the advanced management of wounds throughout all stages.

Hydrogel have become the ideal biomedical material for wound treatment owing to its unique 3D porous network, good swelling properties, and adjustable structure, which not only maintains a moist microenvironment at the wound site, but also allows for loaded nanomaterials and drug responsive release.^[^ ^22^ ^]^ Meanwhile, conductive gel showed excellent performance in wound management. It is suggested the application of ES via conductive gel has considerable promise in promoting wound recovery and regulating inflammatory responses. However, the intricate nature of the diabetic wound environment imposes constraints on hydrogels, which lack the ability to adjust dynamically to their surroundings, despite the presence of various wound‐responsive agents. Therefore, it is crucial to prioritize a flexible delivery system that considers the complex nature of the wound environment, manages Glu and ROS levels in the affected region, averts bacterial infections while reducing further tissue harm from oxidative stress, aids in inflammation control through ES, and guarantee a steady supply of O_2_.

**Scheme 1:**
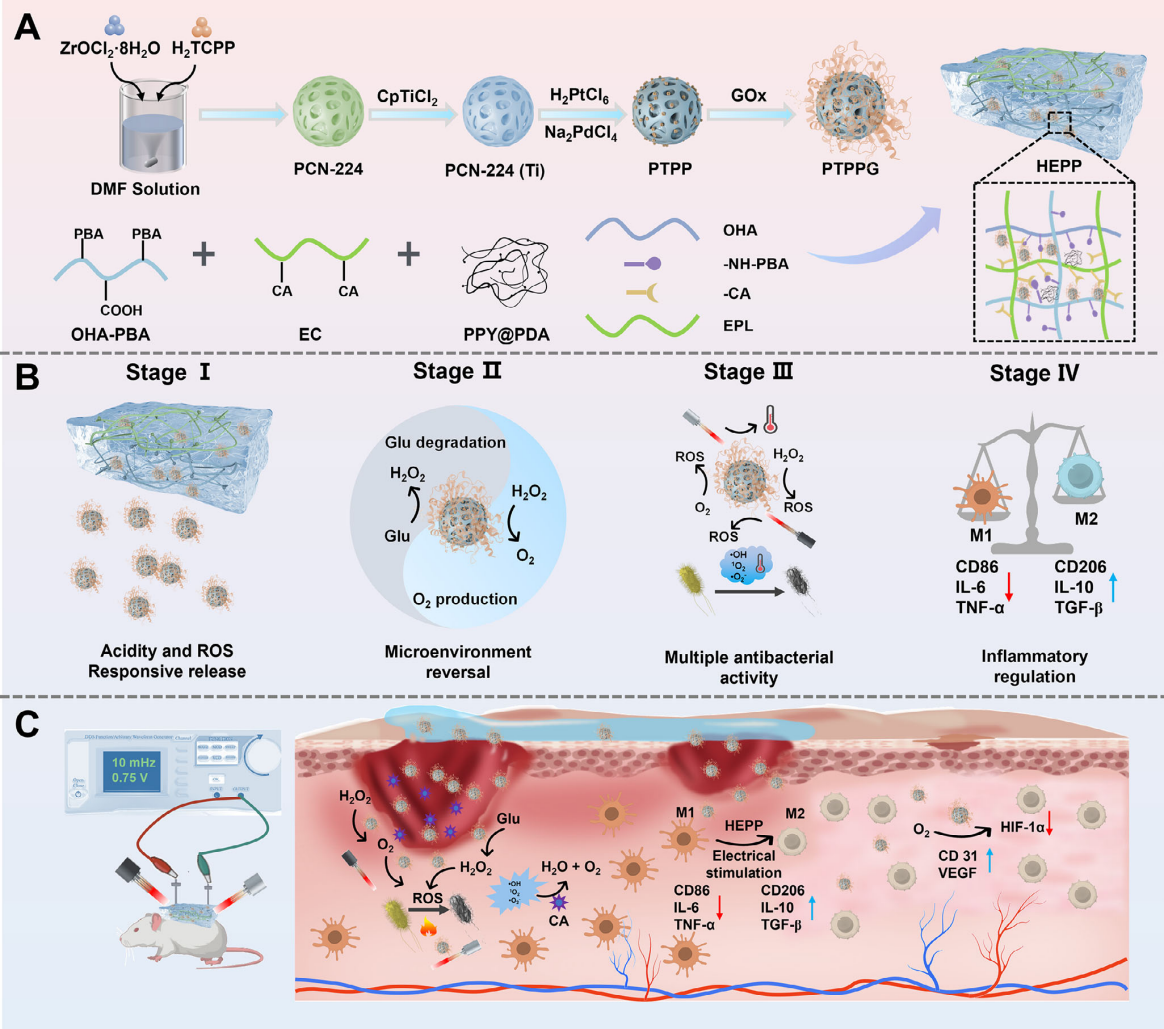
An intelligent conductive immunomodulatory hydrogel for diabetes wound healing. A) A diagram showing the fabrication process of the HEPP. Unless otherwise specified, all HEPP refer to gels with PDA@PPY doping level of 10%. B) HEPP enables responsive release intelligently, microenvironment reversal, antibacterial and inflammatory regulation. C) The immunomodulatory function of the HEPP dressing assisted by ES in the process of diabetic wound recovery.

To accomplish that, we describe here a cutting‐edge hydrogel composed of a framework of hyaluronic acid (HA) modified with phenylboronic acid (PBA) and ε‐polylysine (EPL) linked to caffeic acid, designed to release substances in response to changes in ROS and pH levels, as well as exhibit reactions to NIR and ES. Utilizing Ti‐doped and Pt–Pd nanoparticles along with glucose oxidase (GOx) incorporated into PCN‐244 (PTPPG), we engineered smart hydrogels designed to combat infections and regulate inflammation, thereby facilitating wound healing in diabetic patients. As the pH level within the infected wound dropped, PTPPG functioned as a multifunctional nano‐enzymatic system, delivering a synergistic antimicrobial effect while simultaneously altering the hyperglycemic and hypoxic conditions present at the injury site, owing to its improved PDT effects, diverse enzymatic activities (such as peroxidase (POD), oxidase (OXD), and GOx), along with its photothermal properties. Concurrently, the increased levels of ROS produced by the PTPPG cascade reaction further facilitated the release of nanozymes from the hydrogel. Furthermore, the antioxidant properties of the polyphenol groups on the surface of caffeic acid (CA) allowed for the rapid clearance of excess ROS once physiological conditions were met, thereby preventing secondary damage at the wound site from the elevated ROS environment. By incorporating dopamine@polypyrrole (PDA@PPY) into the hydrogel matrix, the electrical conductivity was markedly increased. The combination of ES and HEPP effectively downregulated the gene expression of TNF‐α, nuclear factor‐k‐gene binding (NF‐*κ*B), and IL‐6, facilitating the transition of macrophages from the pro‐inflammatory phenotype (M1) to the anti‐inflammatory phenotype (M2). As a result, the utilization of NIR‐induced hyperthermia, along with ES, altered the wound microenvironment and offered anti‐inflammatory effects. Studies revealed that HEPP possesses the ability to integrate antibacterial, anti‐inflammatory, and vascular healing capabilities, facilitating a reversal of the microenvironment. This combination enhances infection control and anti‐inflammatory interventions, leading to the creation of a smart wound dressing that is responsive to four distinct triggers (**Scheme** **1**). This investigation foresees that the formulation of advanced conductive hydrogel, characterized by their ability to respond to multiple environmental stimuli and enhance anti‐inflammatory responses via ES therapy, will offer a promising strategy for the treatment of chronic inflammatory disorders.

## 2 Result and Discussion

### 2.1 Synthesis and Characterizations of PTPPG Nanoparticles

**Figure 1:**
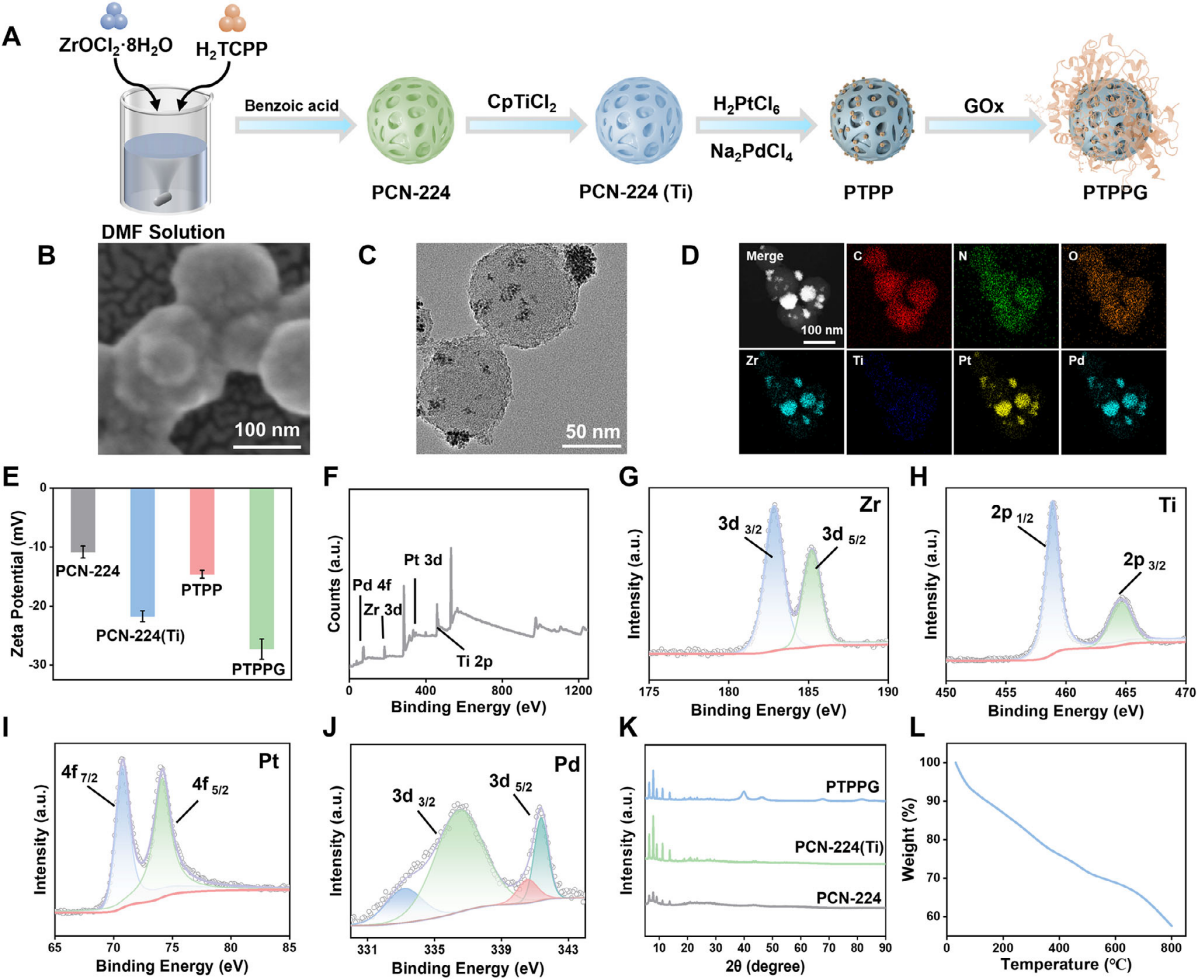
A) The flow chart of PTPPG synthesis. B) SEM, C) TEM and D) EDS analysis of PTPPG. E) Zeta potentials of PCN‐224, PCN‐224(Ti), PTPP and PTPPG. F) XPS survey spectrum of PTPPG. G) XPS spectra of Zr 3d region, H) Ti 2p region, I) Pt 4f region, and J) Pd 3d region. K) XRD patterns of PCN‐224, PCN‐224(Ti) and PTPPG, respectively. L) Thermal gravimetric analysis profile of PTPPG.

The multifunctional nanozymes (PTPPG) were successfully prepared using a multistep process, as illustrated in **Figure** **1**A, the detailed methodology is provided in the experiment section. Briefly, PCN‐224(Ti) nanoparticles with enhanced PDT were synthesized by doping elemental Ti into PCN‐224 via a solvothermal method. Subsequently, Pt–Pd nanoparticles were deposited in situ on the surface of PCN‐224(Ti) using ascorbic acid as a reducing agent. The successful creation of synthetic PTPPG exhibiting GOx, POD, catalase (CAT), and OXD activities were achieved by incorporating GOx into PCN‐224(Ti)@PtPd (PTPP) through surface adsorption. It is predicted that PTPPG will facilitate Glu degradation, exert antimicrobial activity, and generate O_2_ synergistically, thus altering the microenvironment of wounds associated with diabetes.

The structural changes that occurred during the synthesis of PTPPG were meticulously characterized. Initially, the structural characteristics of PCN‐224 were examined using scanning electron microscopy (SEM) and high‐resolution transmission electron microscopy (TEM). As illustrated in Figure S1A and B (Supplementary Information), the synthesized PCN‐224 exhibited a spherical morphology with an approximate diameter of 100 nm. After elemental doping with Ti, the size and morphology of the PCN‐224(Ti) remained largely unaltered, as shown by SEM and TEM images in Figure S2A and B (Supplementary Information). The successful doping of Ti was also confirmed by elemental mapping of TEM (Figure S2B, Supplementary Information) and the doping amount of Ti was determined to be ≈10.58% by inductively coupled plasma‐mass spectrometry (ICP‐MS). Subsequently, the SEM images in Figure S3A and B (Supplementary Information) demonstrated that the Pt‐Pd nanoparticles were uniformly distributed on the surface of PCN‐224(Ti). Furthermore, the energy‐dispersive spectra (EDS) obtained by TEM demonstrated the successful loading of platinum (Pt) and palladium (Pd) elements on the surface (Figure S3C, Supplementary Information). SEM images of PTPPG shown in Figure 1B indicated the improved surface smoothness of PTPPG compared to that of PTPP. TEM images (Figure 1C) also demonstrated the successful loading of Pt‐Pd nanoparticles. Elemental mapping analysis revealed a uniform distribution of the elements carbon (C), nitrogen (N), oxygen (O), zirconium (Zr), titanium (Ti), platinum (Pt), and palladium (Pd) (Figure 1D) on the PTPPG surface. A zeta potential analysis of PTPPG was conducted to demonstrate the successful loading of GOx (Figure 1E). The measured potential of PTPPG became more negative, as shown in the green column, indicating that the GOx had successfully adhered to the PTPP surface. Meanwhile, the surface electronegativity measured by the Zeta potentiometer remained relatively constant, suggesting that the chemical environment of the surface was stable to prevent any performance loss due to agglomeration or surface modifications (Figure 1E).

On this basis, the elemental composition and electronic structure of PTPPG were investigated using X‐ray photoelectron spectroscopy (XPS). The XPS spectrum (Figure 1F) indicated the presence of Zr, Ti, Pt, and Pd within PTPPG, and consistent with the elemental scanning results obtained from TEM. The high‐resolution spectrum of Zr (Figure 1G) was fitted with two characteristic peaks, which are attributed to Zr 3d_3/2_ (182.88 eV) and Zr 3d_5/2_ (185.18 eV), respectively.^[^ ^23^ ^]^ Similarly, the high‐resolution spectrum of Ti (Figure 1H) was convolved into two characteristic peaks, which are attributed to Ti2p_1/2_ (458.88 eV) and Ti 2p_3/2_ (464.68 eV), respectively.^[^ ^24^ ^]^ The high‐resolution spectrum of Pt (Figure 1I) was convolved into two characteristic peaks, which are attributed to Pt4f_7/2_ (70.78 eV) and Pt 4f_5/2_ (74.08 eV), respectively.^[^ ^25^ ^]^ The Pd spectrum reveals four distinct peaks (Figure 1J), with those at 333.18 eV and 340.58 eV corresponding to Pd⁰, while the peaks at 336.58 eV and 341.39 eV are linked to Pd^2^⁺.^[^ ^26^ ^]^ To analyze the nanoparticles changes in crystal structure after Ti doping and Pt‐Pd nanoparticle loading, X‐ray powder diffraction (XRD) analysis was conducted (Figure 1K). It was observed that the characteristic peaks of the PCN‐224 are all presented in PCN‐224(Ti), and PTPPG. The XRD pattern of PTPPG demonstrated that the Pt‐Pd nanoparticles exhibited a typical face‐centered cubic (fcc) structure (blue curve in Figure 1K), with four peaks observed at 39.9°, 46.1°, 67.6°, and 81.7°. These peaks corresponded to the (111), (200), (220), and (311) crystal faces of Pt (JCPDF 04–0802) and Pd (JCPDF 46–1043), confirming the successful synthesis of Pt‐Pd nanoparticles.^[^ ^27^ ^]^ The XRD analysis additionally confirms that PCN‐224 preserves its structural integrity during the cation exchange process, indicating that the doping does not disrupt the periodic channels of MOF, thereby ensuring effective ROS diffusion (Figure 1K). Figure 1L shows that the mass fraction of PTPPG only decreased by ≈7.8% within the temperature range of 0–100 °C, indicating good thermal stability under photothermal conditions. Furthermore, nitrogen (N_2_) physisorption measurements were performed to examine the porosity of the PTPP nanoparticles. The specific surface area of PTPP was measured to be 367.487 m^2^ g^−1^ using the multipoint Brunauer–Emmett–Teller (BET) method. Interestingly, the N_2_ physisorption isotherm displays the characteristics of type II isotherms (Figure S4A, Supplementary Information). The analysis of pore size distribution obtained from the quenched solid density functional theory equilibrium model reveals a considerable amount of both micropores and mesopores within the nanoparticles (Figure S4B, Supplementary Information).^[^ ^28^ ^]^

### 2.2 Multienzymatic Activity and Light Responsiveness of PTPPG

**Figure 2:**
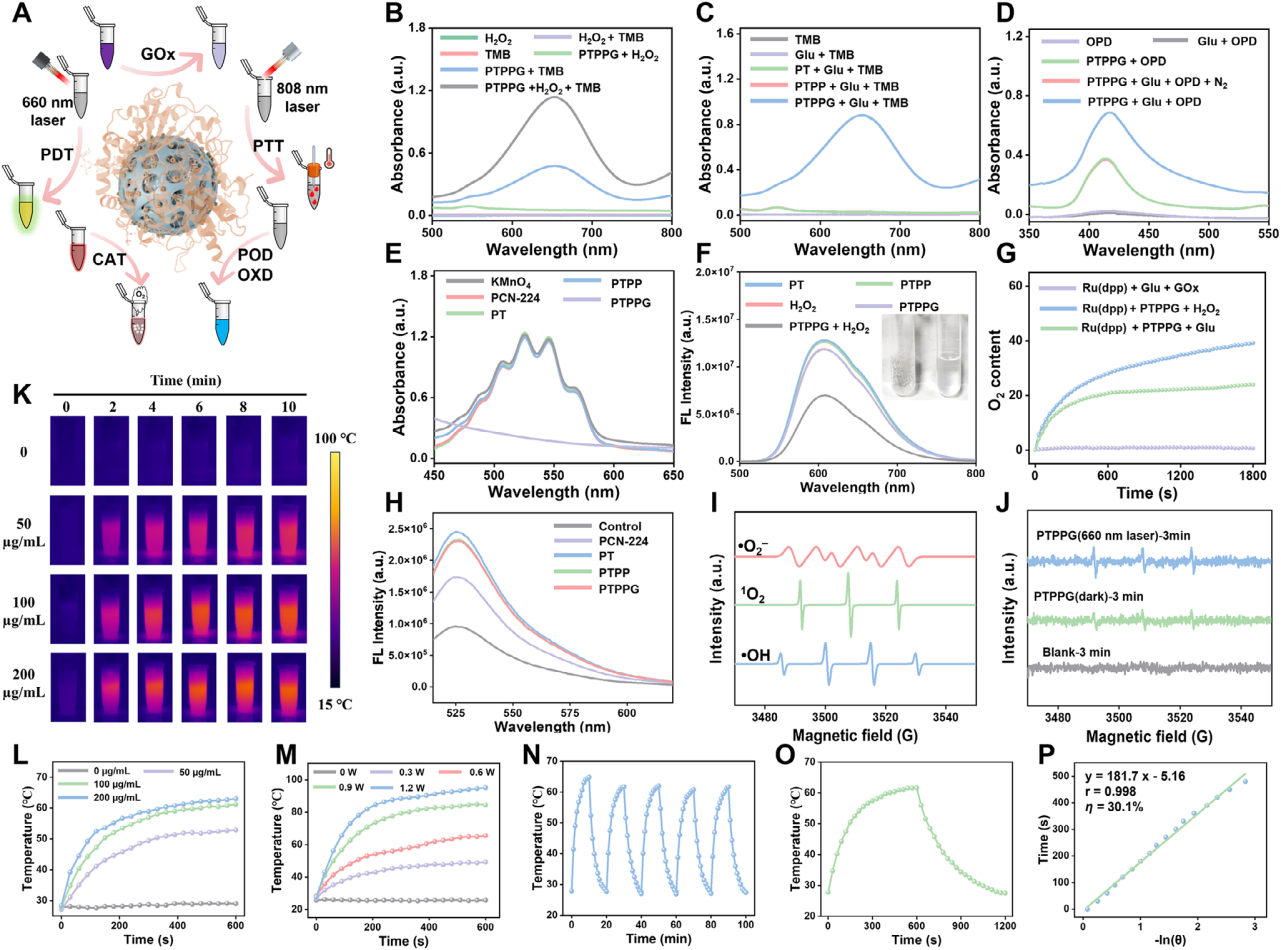
A) The schematic description of multienzymatic activity and light responsiveness of PTPPG. B) The oxidase and peroxidase activity of PTPPG verified by coincubation of PTPPG (5 µg mL^−1^), H_2_O_2_ (5 mM) and TMB (5 µM) in acetate buffer solution (pH 4). Glu oxidase activity at pH of 4 (C) and 7.4 (D) evaluated using acetate buffer solution containing PTPPG and Glu in the presence of TMB/OPD, respectively. Glucose oxidase (E) and catalase activities (F) of PTPPG were verified by coincubation of PTPPG with Glu using KMnO_4_ (10 µg mL^−1^) and Ru(ddp) (250 µM), respectively. G) Continuously monitoring of oxygen generation induced by PTPPG in the presence of H_2_O_2_, Glu with or without GOx, respectively, using Ru(ddp) as the reporting agent. H) Photodynamic enhancement observed by incubating PTPPG with SOSG. I) EPR spectra of •OH, ^1^O_2_ and •O^2–^ obtained with PTPPG incubated with H_2_O_2_, respectively. J) EPR spectra of PTPPG with or without 660 nm laser irradiation (1W, 3 min). K) Photothermal images of PTPPG at different concentrations irradiated by a laser at 808 nm with a power density of 0.9 W. Curves depicting temperature increases at varying PTPPG concentrations (L) and power densities (M) under 808 nm NIR exposure. N) The photostability evaluation of PTPPG exposed to 808 nm laser irradiation (0.9 W) for five cycles. O) The temperature variations of 100 µg mL^−1^ PTPPG during and after laser exposure (808 nm, 0.9 W). P) Linear approximation between −ln(*θ*) and time for PTPPG. Unless otherwise specified, all HEPP refer to gels with PDA@PPY doping level of 10%.

To demonstrate whether PTPPG can become a promising solution to regulate the wound microenvironment and enhance antimicrobial activity, the multiple enzymatic activities, such as GOx, CAT, POD, OXD and light responsiveness, of PTPPG were evaluated in vitro, as shown in **Figure** **2**A. 3,3′,5,5′‐Tetramethylbenzidine (TMB) was used as a substrate to assess the enzymatic activities under acidic conditions. Initially, the enzymatic activities of PTPP were verified by assessing the POD and OXD activities of PTPP (Figure S5, Supplementary Information). Subsequently, Glu was utilized as the reaction substrate to verify the feasibility of the cascade reaction using PTPPG as the mediator under acidic conditions (Figure S6, Supplementary Information). As shown in Figure 2B, PTPPG promoted the conversion of TMB into a blue oxidation state (oxTMB) in the presence of O_2_ or hydrogen peroxide (H_2_O_2_). Meanwhile, Considering the pH of the wound site could dynamically change due to varying conditions of wound infection, TMB and phenylenediamine (OPD) were used as chromogenic substrates under acidic (pH 4) and neutral or weakly basic (pH 7.4) conditions, respectively, to explore the feasibility of initiating a cascade reaction using PTPPG under the complicated pH dynamics in wound sites. As shown in Figure 2C,D, PTPPG degraded Glu at the wound site under both acidic and neutral conditions. The oxidation of TMB (oxTMB) in a solution containing Glu can solely be facilitated by PTPPG (Figure 2C). Figure 2D verifies that PTPPG catalyzes the oxidation of the substrate (OPD) through Glu catabolism at pH of 7.4, as evidenced by a change of absorbance at 415 nm (The UV absorption peaks at 415 nm for green and red curves in Figure 2D is due to the characteristic UV absorption peak of tetrakis(4‐carboxyphenyl) porphyrin (TCPP) in PTPPG). The production of H_2_O_2_ during the cascade reaction was confirmed using potassium permanganate (KMnO_4_) as a H_2_O_2_ detection reagent.^[^ ^29^ ^]^ As shown in Figure 2E, the absorbance of KMnO_4_ (0.5 mM) decreased by 91.3% in the PTPPG‐treated group in a solution containing 5 mM Glu as the substrate at a pH of 4. The absorbance of KMnO_4_ at 525 nm exhibited a decreasing trend with the increasing concentration of PTPPG (0–10 µg mL^−1^) and Glu (0–5 mM) (Figure S7A and B, Supplementary Information), successfully verifying that GOx on PTPPG with high enzymatic activities catalyzed the Glu oxidation in solution phase at pH of 4.

We further investigated the CAT activity of PTPPG using tris(4,7‐diphenyl‐1,10‐phenanthroline)ruthenium (II) dichloride (Ru(ddp)) as the report reagent^[^ ^30^ ^]^ As a fluorescent probe for O_2_, Ru(ddp) emits red fluorescence in hypoxic environments. As shown in Figure 2F, the presence both of PTPPG and H_2_O_2_ quenched the fluorescence signal of Ru(ddp). Meanwhile, the change in O_2_ concentration over 30 min was monitored by fluorescence methods (Figure 2G and Figure S8, Supplementary Information), and the change in Ru(ddp) fluorescence signal at 613 nm in a solution containing Ru (ddp), Glu and PTPPG verified PTPPG degraded Glu and produced O_2_ as the final product, thereby alleviating wound hypoxia and regulating the wound microenvironment. The radicals generated by PCN‐224 during light irradiation was evaluated using Singlet Oxygen Sensor Green (SOSG) as a singlet oxygen (^1^O_2_) fluorescent probe. The TCPP content in PTPPG was calculated to be 18.67% using the linear equation of TCPP and PTPPG obtained from Figures S9 and S10 (Supplementary Information). As illustrated in Figure 2H and Figure S11 (Supplementary Information), the photodynamic performance of Ti‐doped PCN‐224 (PT) was augmented ≈1.5 times following doping with the Ti element, demonstrating doping with the Ti element enhanced the efficacy of PDT. Additionally, the generated ^1^O_2_ increased with the rise of laser power density at 660 nm and PTPPG concentration (Figure S12, Supplementary Information). The generation of hydroxyl radicals (•OH), ^1^O_2_, and superoxide radicals (•O^2–^) was confirmed through electron paramagnetic resonance (EPR) spectroscopy during the reaction process to demonstrate PTPPG exerted multienzyme activity, as illustrated in Figure 2I. Furthermore, to substantiate that ^1^O_2_ is not solely derived from OXD activity, EPR spectroscopic peak signals of 1.94 × 10^−7^ and 2. 6 × 10^−7^ were obtained with and without 660 nm laser irradiation after the reaction for 3 min, respectively (Figure 2J), which illustrate that the generation of ^1^O_2_ radicals was collectively induced by NIR (660 nm) irradiation and OXD activity of PTPPG. Subsequently, the mechanism of the enhanced photodynamic properties of PT was further explored. The incorporation of Ti into PCN‐224 leads to a reduction in the photoluminescence (PL) of the TCPP ligand, as shown in Figure S13A (Supplementary Information), which indicates the increased charge transfer from the ligand to the metal and a decrease in the bandgap.^[^ ^31^ ^]^ The presence of a distinct vibrational mode at 664 cm^−1^, as shown in Figure S13B (Supplementary Information), indicates the coordination of Ti‐O, confirming that titanium has replaced zirconium at the original Zr‐node sites.^[^ ^24^ ^]^ This alteration in structure promotes the separation of charges at the interface, resulting in a 1.5 times enhancement in the production of singlet oxygen (^1^O_2_), as shown in Figure 2H.^[^ ^32^ ^]^ Furthermore, as suggested by earlier reports, Zr‐Ti‐oxo clusters enhance the movement of photoelectrons from TCPP to metal nodes more effectively than Zr‐oxo clusters, which increases the production of ROS.^[^ ^24^, ^33^ ^]^

We then thoroughly investigated the NIR photothermal properties of PTPPG. Firstly, different concentrations of PTPPG were subjected to a laser irradiation with a wavelength of 808 nm at 0.9 W. An infrared camera was employed to obtain real‐time thermal images of the PTPPG (Figure 2K). As illustrated in Figure 2L, the solution temperature increased with increasing PTPPG concentrations. Furthermore, the impact of laser power was examined using an 808 nm laser with power ranging from 0 to 1.2 W, with thermal images recorded at 2‐minute intervals (Figure S14, Supplementary Information). Moreover, compared with the temperature change in the first heating and cooling cycle, the temperature fluctuation is negligible in the following four cycles, illustrating that the PTPPG showed good photothermal stability and can be repeatedly used for PTT (Figure 2N). The final photothermal conversion efficiency was determined to be 30.1% for a solution containing 100 µg mL^−1^ PTPPG and irradiated at 0.9 W (Figure 2O,P). We further evaluated whether laser irradiation at 660 or 808 nm will affect the enzymatic activities of GOx loaded on PTPPG. The characteristic visible peak of potassium permanganate was obtained, as illustrated in Figure S15 (Supplementary Information). The lasers at 660 and 808 nm exhibited minimal impact on GOx activity after a 5‐minute treatment period. Based on the photothermal activity of PTPPG, an investigation was conducted to evaluate the effect of temperature in the cascade reaction of PTPPG. As illustrated in Figures S16 and S17 (Supplementary Information), the kinetic profiles of the reactions at pH 4 and 7.4 were markedly enhanced at 50 °C compared to those at room temperature, respectively, indicating that the photothermal‐induced temperature accelerated the enzymatic cascade reaction. Taken together, the in vitro experimental results suggest that photothermal‐enhanced microenvironmental regulation could be a promising approach for treating wounds caused by hyperglycemia and O_2_ deprivation.

### 2.3 Synthesis and Characterizations of HEPP

**Figure 3:**
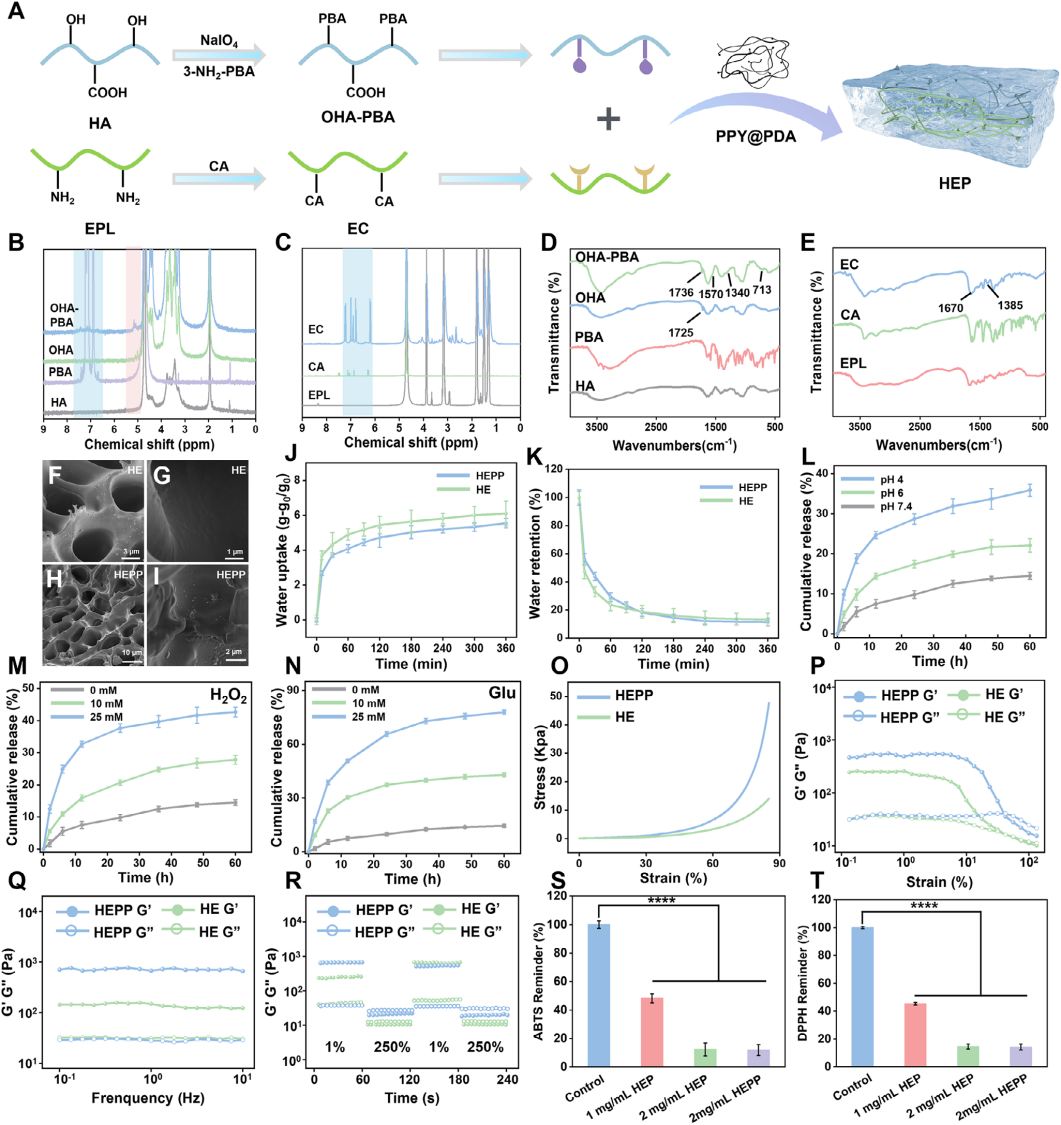
A) The flow chart of HEPP synthesis. The ^1^H NMR spectra of OHA‐PBA (B) and EC (C). FT‐IR spectra of OHA‐PBA (D) and EC (E). Sections (F)–(I) are SEM images of the HE and HEPP obtained at different magnification levels. Section (J) and (K) are water uptake and retention curves obtained from HEPP and HE, respectively. Release rate of PTPPG at different pH values (L), H_2_O_2_ (M) and Glu concentrations (N). O) The adhesive strength of HE and HEPP. P) Analysis of strain‐responsive rheological behavior of HE and HEPP at a frequency of 0.1 Hz, respectively. Q) Frequency‐responsive rheological characteristics of HE and HEPP under a 1% strain condition, respectively. R) Step‐strain tests conducted on GHM hydrogels at low (1%) and high (250%) strains with a set frequency of 0.1 Hz. Scavenging of DPPH (S) and ABTS (T) by HEPP. Unless otherwise specified, all HEPP refer to gels with PDA@PPY doping amount of 10%. Error bars represent the mean ± standard deviation for a sample size of 3. *p* values were calculated via one‐way ANOVA test. *p < 0.05, **p < 0.01, ***p < 0.001, ****p < 0.0001.

It is well known chronic nonhealing wounds were originate from the gradually decreased pH followed by bacterial infection and inflammation caused by diabetes, resulting in the accumulation of ROS at the wound site. Hence, it is essential to develop an intelligent hydrogel that reacts to particular circumstances, such as pH and ROS, presented at the wound location. To accomplish that, a smart conductive hydrogel was developed through the combination of dynamic Schiff base linkages, boronic ester bonds and the incorporation of highly conductive PDA@PPY.^[^ ^34^ ^]^ Besides that, a pH‐responsive monomer (OHA‐PBA) was synthesized by reacting the aldehyde group on oxidized hyaluronic acid (OHA), obtained from the oxidation of periodate‐treated HA (Figure S18, Supplementary Information), with the amino group on 3‐aminophenylboronic acid (3‐NH_2_‐PBA), as illustrated in **Figure** **3**A. In the ^1^H‐NMR analysis of OHA and OHA‐PBA, the peak observed at 1.94 ppm was attributed to the CO–CH_3_ group presented in HA, with a series of peaks between 3.24–4.47 ppm corresponding to the compound peak of HA, OHA and OHA‐PBA.^[^ ^35^ ^]^ The ^1^H‐NMR spectra for OHA and OHA‐PBA showed distinct peaks at 4.98–5.2 ppm, which are characteristic of hemiacetal protons, as represented in Figure 3B and Figure S19A (Supplementary Information).^[^ ^35^ ^]^ This finding indicates that HA is subject to oxidation, leading to the formation of aldehydes. The oxidation level of the aldehyde was determined to be 10.5% by analyzing the integrated areas of the protons at 4.98–5.2 ppm for the aldehyde and at 1.94 ppm for the methyl (–CH_3_) group, as indicated by the ^1^H‐NMR spectra of OHA.^[^ ^36^ ^]^ Additionally, the signals observed between 6.8–7.2 ppm for OHA‐PBA align with the distinctive peaks associated with the benzene ring of PBA, as illustrated in Figure 3B and S19B (Supplementary Information).^[^ ^37^ ^]^ As illustrated in the Fourier Transform Infrared spectra (Figure 3D), the absorptions at 1725 and 1736 cm^−1^ for OHA and OHA‐PBA, respectively, are associated with the stretching vibrations of the aldehyde group (C═O), which substantiate the effective oxidation of HA. Furthermore, the absorptions at 1340 and 713 cm^−1^ were attributed to the B–O stretching and C–H bending vibrations of phenylboronic acid, respectively, while the absorption at 1570 cm^−1^ was attributed to the imine bond, indicating that a Schiff base had been formed between OHA and 3‐NH_2_‐PBA. The successful loading of 3‐NH_2_‐PBA onto OHA was also indicated by changes in the UV characteristic curves (Figure S20A, Supplementary Information).^[^ ^38^ ^]^ These results demonstrate the successful synthesis of OHA‐PBA.

Subsequently, CA, known for its antioxidant capabilities, was coupled with EPL through an amidation reaction (Figure S21, Supplementary Information) to construct precursors (EC) with antioxidant properties. As illustrated in the ^1^H‐NMR spectrum in Figure 3C, the characteristic peaks associated with EPL and CA were detected in EC, indicating the successful branching of CA onto EPL. Furthermore, an increase in peak intensity at 1670 cm^−1^ in EC polymer, as observed in the Fourier transform infrared (FT‐IR) spectroscopy in Figure 3E was attributed to the stretching vibration of the amide bond. Additionally, the O‐H bending vibration of EC observed at 1385 cm^−1^ provides further evidence of the successful branching of the EC polymer. The UV spectra of EC exhibited comparable absorption peaks at 298 and 325 nm, similar to those observed for CA (Figure S20B, Supplementary Information). This further substantiates the successful binding of CA to EPL without oxidation of the phenolic hydroxyl group. After that, OHA‐PBA was cross‐linked with the EC polymer to form borate bonds, thereby producing HE hydrogels that respond to changes in pH and ROS. As illustrated in Figure S22 (Supplementary Information), when the HE was treated with 100 mM H₂O₂ at pH of 4, the hydrogels exhibited a notable increase in mobility compared to the untreated hydrogels, indicating their responsiveness to pH and ROS. On this basis, the synthesized PDA@PPY nanowires (Figure S23, Supplementary Information) were physically doped into the hydrogel to enhance the conductivity of HEP. The doping processes applied to PTPPG led to the formation of HEPP. The morphology of the HE and HEPP was characterized by SEM. Figure 3F,H illustrate the three‐dimensional network structure of the HE and HEPP, respectively. It is noteworthy that higher magnification SEM images revealed that the interior of HE exhibited a smooth texture (Figure 3G), while the interior of HEPP displayed a rough surface due to the presence of both PDA@PPY and PTPPG (Figure 3I).

Subsequently, water uptake and retention properties of HE and HEPP were evaluated. As illustrated in Figure 3J, the water uptake patterns of HE and HEPP exhibited notable similarities. Initially, there was a rapid uptake of water for the first 30 min, which then gradually stabilized over the subsequent 120 min. After reaching the steady state, the water uptake rate of HE was slightly higher than that of HEPP, indicating that the addition of PDA@PPY and PTPPG showed a less pronounced effect on water uptake. To ascertain the water retention capacity of the hydrogels, both HE and HEPP were dried in a fume hood at 37 °C until no further change in weight was observed. A similar water retention capacity was observed, as shown in Figure 3K. The results indicate that HEPP could play a significant role in the effective absorption of wound exudate and the promotion of diabetic wound healing.

To evaluation how the local conditions at the wound site affect the release of PTPPG, an investigation was conducted to examine how the release of PTPPG influence the pH levels. The results showed that GOx loaded on PTPP could reduce pH by decomposing Glu (Figure S24, Supplementary Information). Furthermore, Figure 3L demonstrates that lower pH levels led to an increase in the release of PTPPG. This is mainly due to the protonation of the imine group of the Schiff base in the acidic environment, which weakens the strength of the C═N bond and promotes the hydrolysis of the Schiff base bond in the gel, resulting in increased porosity of the gel and accelerated release of PTPPG.^[^ ^39^ ^]^ Similarly, it was discovered that the release rate of PTPPG increased as the levels of H_2_O_2_ were elevated (Figure 3M), owing to the increased amount of ROS generated. A solution containing 25 Mm Glu showed the highest release rate of PTPPG (Figure 3N), likely because the process of Glu degradation induced by PTPPG not only decreases pH but also elevates ROS levels, creating a combined effect that boosts nanoparticle release. As illustrated in Figure S25 (Supplementary Information), HEPP presented in a 10 mM Tris‐HCl buffered solution (pH 7.4) in the presence and absence of 50 mM Glu solution showed a remarkable difference in the release of PTPPG. These results successfully demonstrated the pH and ROS responsiveness of HEPP and further evidence that HEPP dressings can effectively reverse the diabetic wound microenvironment, thereby facilitating optimal therapeutic outcomes.

Hydrogel dressings are required to exhibit robust mechanical elasticity and adaptability to effectively address the dynamic and complex characteristics of wound microenvironments. Hence, the rheological properties of HE and HEPP underwent a comprehensive evaluation, as illustrated in Figure 3O–R. The compressive properties of the hydrogels were evaluated using an electronic universal testing machine (Figure 3O). As anticipated, the compression modulus of HEPP was found to be 2.4 times greater than that of HE. This increase can be attributed to the enhanced cross‐linking points of the hydrogel resulting from the incorporation of PDA@PPY and PTPPG. Moreover, strain scanning tests were performed to determine the linear viscoelastic region of the hydrogel (Figure 3P). At ≈85% strain, the HEPP exhibited gel points, which subsequently transformed into a colloidal state beyond this strain threshold. Moreover, the behavior of the hydrogel was examined at varying frequencies (ranging from 0.1 to 10 Hz). As illustrated in Figure 3Q, the energy storage modulus (*G*′) of the hydrogel consistently exceeded the loss modulus (*G*″), suggesting that both HEPP and HE exhibit elastic properties at all measured frequencies.^[^ ^40^ ^]^ Following that, a substantial strain of 250% was applied to disrupt the hydrogel's crosslinked network, and then a mild strain of 1% was employed to evaluate its capacity to recuperate (Figure 3R). Despite a notable decline in *G*″ at 250% strain, indicative of network disruption, a swift recuperation of *G*′ and *G*″ to their initial states was discerned upon return to 1% strain, underscoring the pronounced self‐healing attributes of the hydrogels. The stretching property of hydrogels facilitates its contact with irregular wound areas, while self‐healing hydrogels can withstand external mechanical forces and prolong the service life of hydrogel wound dressings. As illustrated in Figure S26 (Supplementary Information), the HE hydrogels exhibited favorable stretchability, adhesion, and hydrogel recovery properties. In addition, to confirm the tensile characteristics of the gels more thoroughly, we utilized an electronic universal testing machine to evaluate the tensile properties of gels with differing compositions. The tensile characteristics of the HEPP, as shown in Figure S27A (Supplementary Information), are outstanding, with an impressive elongation at break reaching 1799.2%. In contrast to gels infused with PTPPG, the notable enhancement in tensile characteristics of gels attributed to the incorporation of PDA@PPY can be explained through several factors. These include i) the formation of pH‐responsive boronate ester bonds between catechol moieties of dopamine and the gel matrix; ii) the reinforcement at the interface via dual interactions of π–π stacking and hydrogen bonding between of polypyrrole and phenolic hydroxyl groups in hydrogel precursor of EC; and iii) the creation of physical crosslinks from catechol‐amine interactions within the ε‐polylysine structure, facilitated by the oxidative self‐polymerization of PDA.^[^ ^41^ ^]^ Meanwhile, the HEPP was placed on the finger to mimic the variations and suitability in practical scenarios (Figure S27B, Supplementary Information). Furthermore, the hydrogel needs to possess sufficient adhesive properties to guarantee that it remains attached to the skin throughout the treatment process. The strength of adhesion for the hydrogel was assessed via a simulation experiment involving pig skin. The adhesive properties were evaluated using an electronic universal testing machine, as illustrated in Figure S28A (Supplementary Information), following the application of the hydrogel onto the intermediate layer of two pigskin samples. In comparison to the adhesive properties of fibrin glue, both HE and HEPP exhibited superior adhesion strength, ensuring effective bonding to the wound surface (Figure S28B, Supplementary Information). The mechanism by which the hydrogel adheres to skin tissue is believed to involve the catechol moiety of caffeic acid and the ester bond formed with boric acid in the gel, which interacts with proteins like collagen and glycoproteins at the wound site.^[^ ^42^ ^]^ Additionally, Figure S29 (Supplementary Information) revealed that HEPP exhibits strong sticking properties to pigskin and various mouse organs, including the heart, liver, spleen, lungs, and kidneys, underscoring its potential to closely attach to wounds in practical settings, thereby aiding in the immune regulation at these sites. These findings substantiate the distinctive mechanical characteristics of HEPP, thereby underscoring its potential to serve as an immunomodulatory agent at diabetic wound sites. In addition, the incorporation of caffeic acid as a linker unit in the formulation of HEPP not only ensures the responsiveness of hydrogel but also endows it with antioxidant properties. As illustrated in Figure 3S,T, the scavenging efficacy of free radicals 1,1‐diphenyl‐2‐picrylhydrazyl (DPPH) and ABTS by HEPP reached 90% in the in vitro test, thereby substantiating the hydrogel's antioxidant capacity.

Subsequently, the conductivity and photothermal properties of HEPP were evaluated at varying doping levels of PDA@PPY (0%, 5%, 10%, 20%).^[^ ^43^ As depicted in Figure S30 (Supplementary Information), the conductivity of HEPP experienced a considerable increase when more PDA@PPY was mixed into the HE. Real‐time thermograms were obtained using an infrared camera under 808 nm laser irradiation (Figure S31, Supplementary Information). The photothermal conversion efficiency of the HEP hydrogel was determined to be 36.3% with 10% doping levels of PDA@PPY at 0.9 W (Figure S32, Supplementary Information). Similarly, PTPPG (1 mg mL^−1^) were doped onto HEP hydrogel (varying doping levels of PDA@PPY) to form 0%, 5%, 10%, and 20% doped HEPP, and real‐time thermal images were recorded (Figure S33, Supplementary Information). The photothermal conversion efficiency of HEPP increased to 38.8% under the same conditions (Figure S34, Supplementary Information). The HEPP, which have been constructed with integrated photo/electro/pH/ROS responsiveness, demonstrate considerable potential for use in the field of biomedical materials, where they could be employed for the intelligent response and treatment of wounds.

### 2.4 Antibacterial Activity of HEPP In Vitro

**Figure 4:**
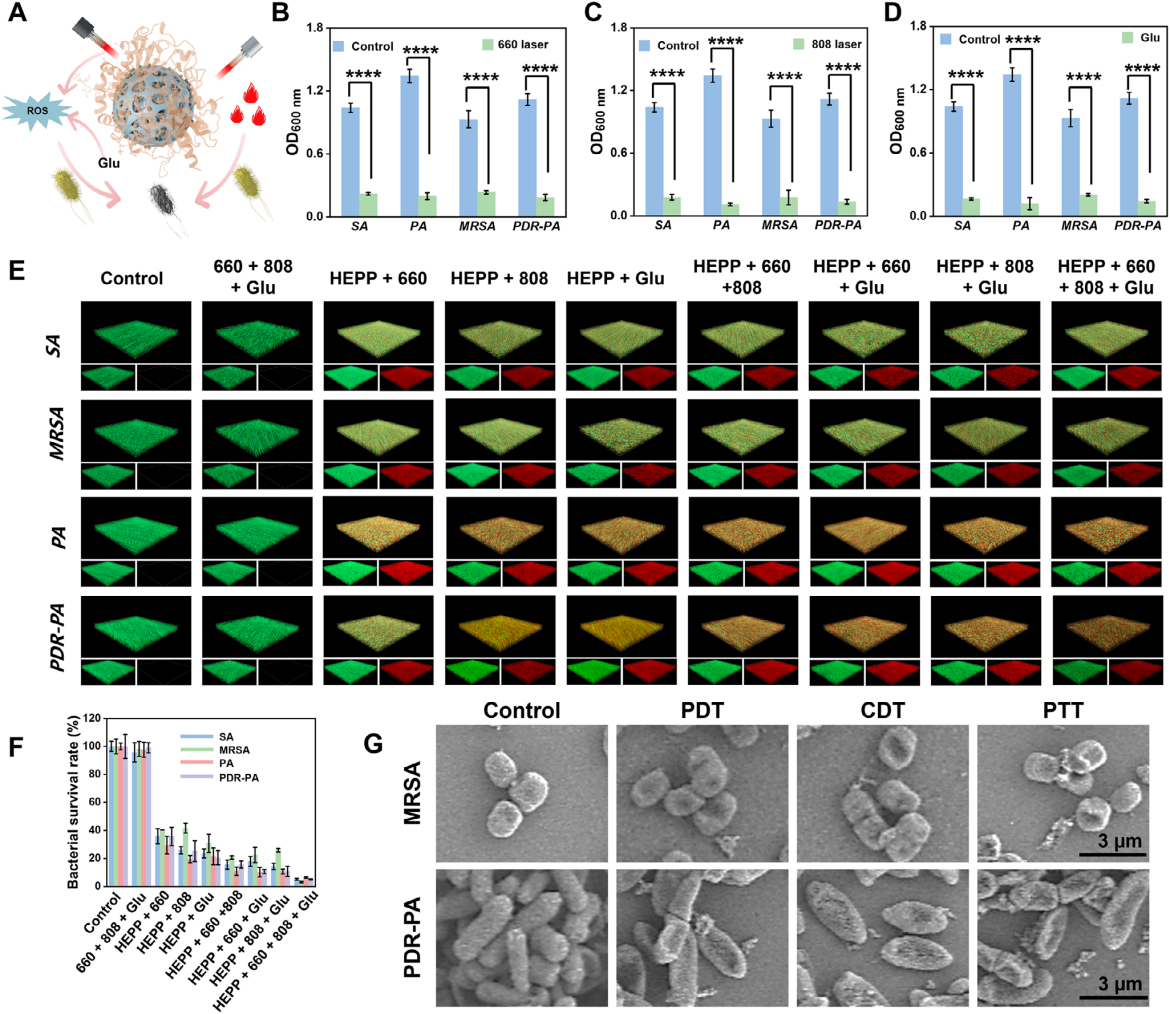
A) An illustrative diagram depicting multiple mechanisms of antibacterial activity. The OD 600 nm values of Gram‐negative bacteria and Gram‐positive bacteria treated or untreated with 660 nm (B), 808 nm (C) laser and Glu (D) for 5 min, respectively.  Fluorescent images (E) and corresponding quantitative statistical analysis (F) of the live/dead stained bacteria (SA, MRSA, PA and PDR‐PA) in PBS (pH 7.4) after treatment with different conditions. G) SEM images of bacteria subjected to different treatments using HEPP. Error bars represent the mean ± standard deviation for a sample size of 3. *p* values were calculated via one‐way ANOVA test. *p < 0.05, **p < 0.01, ***p < 0.001, ****p < 0.0001.

Wounds in diabetic patients are more prone to microorganism infections, but macrophages and neutrophils are capable of eliminating invading microorganisms within their phagosomes by producing elevated levels of ROS. The localized decrease in acidity at the infection site, triggered by the presence of bacteria, leads to the disruption of Schiff base linkages at various pH levels. The released PTPPG facilitate the decomposition of Glu, which subsequently produces H_2_O_2_ and results in the breakage of the borate bond. This process facilitates the accumulation of PTPPG at the wound site, facilitating the self‐cascade antimicrobial actions of ROS in wounds associated with diabetes. Given the evidence outlined, it can be inferred that the HEPP shows a cooperative antimicrobial effect that is triggered by light (**Figure** **4**A). To demonstrate that, the Gram‐positive strains *Staphylococcus aureus* (*SA*) and *methicillin‐resistant Staphylococcus aureus* (*MRSA*), as well as the Gram‐negative strains *Pseudomonas aeruginosa* (*PA*) and *polydrug‐resistant Pseudomonas aeruginosa* (*PDR*‐*PA*), were selected as representative strains. The initial step was to investigate the antimicrobial properties of the HEPP under 660 nm laser irradiation. As illustrated in Figure S35 (Supplementary Information), the optimal photodynamic antimicrobial conditions (1 W, 5 min) were determined by optimizing the power intensities of the 660 nm laser, the PDA@PPY loading rate of HEPP, and the irradiation time. Compared to the control group, the absorbance of the bacteria strains at 600 nm was markedly diminished in the groups treated under 660 nm laser irradiation, and the antimicrobial efficacy against the four bacteria exceeded 75% (Figure 4B). Subsequently, the impact of Ti doping on the photodynamic antibacterial performance was investigated. The antibacterial efficacy of TCPP was compared with that of PCN‐224, and it was found that Ti doping significantly enhanced the photodynamic performance (Figure S36, Supplementary Information). Similarly, the photothermal antibacterial effect was optimized in a manner analogous to that described above (Figure S37, Supplementary Information).

As illustrated in Figure 4C, the absorbance of bacteria at 600 nm decreased by ≈93% following 808 nm laser irradiation at 0.9 W for 5 min. In order to mimic the antibacterial response induced by ROS at the wound area, 20 mM of Glu was utilized. As the PTPPG loading in the HEPP increased, caused a concentration‐dependent decline at the OD600 nm (Figure S38, Supplementary Information). Thus, the potential for self‐cascading antibacterial activity by nano‐enzymes at the wound site was validated through a comparison of the bacterial viability at the OD600 nm (Figure 4D). Moreover, the integrated photodynamic, photothermal, and chemical kinetic antimicrobial characteristics of HEPP were examined using bacterial colonization (Figure S39, Supplementary Information) with bacterial live/dead fluorescent staining (Figure 4E). The bacterial colony counts and quantitative fluorescence analysis (Figure 4F) demonstrated that the combination of the three therapies showed the most substantial antimicrobial impact. Furthermore, as illustrated in Figure 4G, SEM images revealed that bacteria of *MRSA* and *PDR‐PA* that were subjected to PDT/PTT/CDT exhibited a loss of normal membrane structure and evidence of rupture. Collectively, these findings suggest that HEPP possesses remarkable antimicrobial characteristics and holds significant potential for preventing bacterial infections within the wounds.

### 2.5 Microenvironmental and Inflammatory Regulation via HEPP Assisted by ES

**Figure 5:**
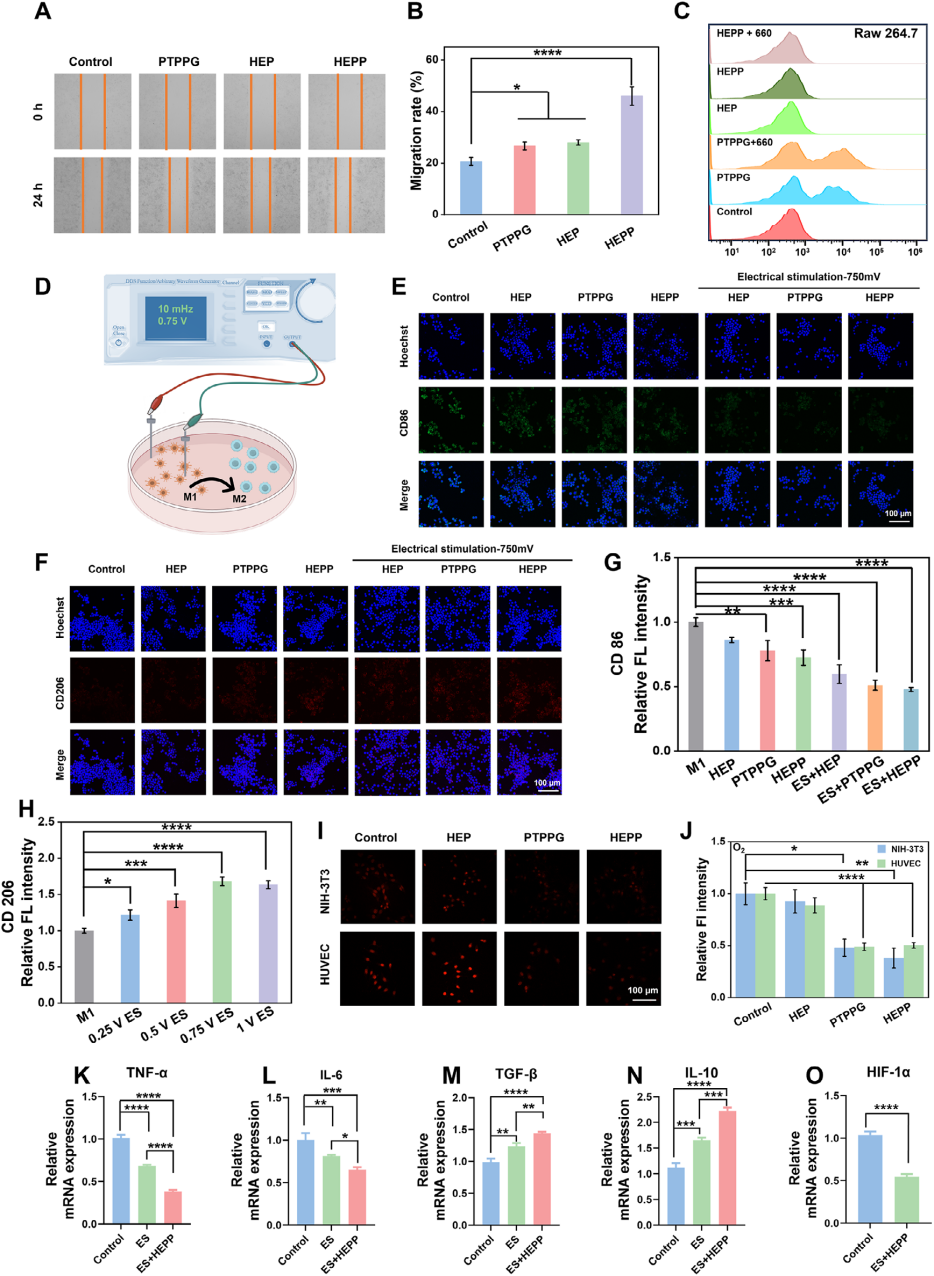
A) Scratches micrographs of HUVEC and B) corresponding quantitative histograms of HUVEC migration ratios after various treatments. C) Flow cytometric results of DCFH‐DA fluorescence in RAW264.7 cells after being treated under different conditions. D) Schematic diagram of ES treatment of macrophages to regulate inflammatory phenotype. Immunofluorescence staining of CD86 (E) and CD206 (F) expression in RAW264.7 cells under different treatment conditions. Assessment of relative fluorescence intensities of CD86 (G) and CD206 (H) in RAW264.7 cells. Fluorescence images (I) and relative fluorescence intensities (J) of dissolved O_2_ evaluated by Ru(dpp) using NIH‐3T3 and HUVEC cells, respectively, after being treated with different materials. (K)–(O) qRT‐PCR analysis of mRNA expressions corresponding to TNF‐α, IL‐6, TGF‐β, IL‐10, and HIF‐1α within RAW264.7 cells after different treatments, respectively. Error bars represent the mean ± standard deviation for a sample size of 3. *p* values were calculated via one‐way ANOVA test. *p < 0.05, **p < 0.01, ***p < 0.001, ****p < 0.0001. Unless otherwise specified, all HEPP refer to gels with PDA@PPY doping amount of 10%.

To assess the biocompatibility of PTPPG, HEP, and HEPP, the toxicity of these materials to Mouse embryonic fibroblast cells (NIH‐3T3), mouse leukemia cells of monocyte macrophage (RAW264.7), and Human Umbilical Vein Endothelial Cells (HUVEC) were quantified through co‐incubation them with the hydrogels using CCK‐8 assay (Figures S40–S42, Supplementary Information). Cell viabilities were found to be greater than 90% in PTPPG, HEP, and HEPP‐treated groups, indicating that the HEPP exhibited negligible toxicity under physiological conditions. Subsequently, the cytotoxicity of these nanomaterials mentioned above was evaluated using live/dead cell staining technique. Following a 24‐hour incubation period, the NIH‐3T3, RAW264.7, and HUVEC cells were stained using a live/dead cell viability kit (Calcein‐AM/PI). The live cells were stained green due to the presence of Calcein‐AM, while the dead cells were stained red due to the binding of propidium iodide (PI). The fluorescence images from the three treatment groups demonstrated green fluorescence intensities were similar to those observed in the control group (Figures S43–S45, Supplementary Information), indicating that the hydrogels exhibited favorable biocompatibility. Moreover, a scratch test was performed on the hydrogels to evaluate their ability to facilitate fibroblast migration in vitro (**Figure** **5**A). Figure 5B shows that the PTPPG group (26.7%), the HEP group (28.0%), and the HEPP group (46.0%) exhibited superior scratch healing capabilities compared to the control group (20.6%) after 24‐h incubation. The significantly enhanced wound healing at the 24‐h mark using HEPP demonstrated that cell migration was not significantly hindered either before or after the incorporation of the hydrogel, thus further corroborating the exceptional biocompatibility of the HEPP.

After bacteria strains were killed by elevated ROS generated by PTPPG released from HEPP, an excess of ROS can lead to excessive release of inflammatory cytokines, resulting in heightened inflammation of wounds. Consequently, elimination of the excess ROS generated at the wound site can significantly accelerate the healing process. Following that, the cellular antioxidant properties of HEPP were investigated by incubating HEPP with cells. As illustrated in Figures S46–S48 (Supplementary Information), the addition of PTPPG in the presence of 660 nm laser irradiation led to a significant increase in ROS concentrations. However, following a 4 h treatment, a notable reduction in cellular ROS levels was observed compared to the control group. Moreover, the influence of various treatments on intracellular ROS was assessed through flow cytometry. As illustrated in Figure 5C, the cascade reaction of nanoparticles and photodynamic treatment resulted in a marked elevation of ROS levels in RAW264.7 cells. Hydrogel incubation significantly lowered the levels of ROS in the cells, effectively averting potential negative consequences associated with elevated ROS levels. Figure S49 (Supplementary Information) also show a comparable pattern in ROS regulation with NIH‐3T3 and HUVEC cells. These findings substantiate the capacity of HEPP to scavenge ROS, thereby providing compelling evidence of its antioxidant properties.

We further investigated the capabilities of these conductive HEPP in amelioration of the inflammatory microenvironment of wounds by depleting Glu and scavenging ROS, thereby promoting the shift of macrophages from M1 to M2 phenotype during ES. As illustrated in Figure 5D, the inflammatory changes were also investigated at cellular level with or without ES, with M1 phenotype serving as a control. To accomplish that, a positive control was established by the stimulation of an M1 phenotype using lipopolysaccharide (LPS). After that, macrophages were subjected to ES using alternating current (0–1000 mV, 250 mV interval). To ascertain the polarization state of RAW264.7 macrophages, immunofluorescence staining was employed to visualize CD86 (a marker for M1) and CD206 (a marker for M2). As illustrated in Figures S50A and S51A (Supplementary Information), the expression of CD86 was most pronounced in the context of LPS‐induced inflammation. In the electrically stimulated treatment group, a decrease in CD86 expression and an increase in CD206 expression were observed compared to the control group and the highest value of fluorescence intensities for CD206 was obtained in the treatment groups receiving ES at voltages of 750 mV and 1000 mV (Figures S50B and S51B, Supplementary Information). The results presented in Figure 5E–H illustrates that the shift from M1 to M2 phenotype was most pronounced when cells were treated with HEPP and electric stimulation simultaneously, owing to the largely eliminated CD86 fluorescence signal and increased CD206 fluorescence signal shown in Figure 5G,H, respectively. Moreover, as demonstrated in Figures S52 and S53 (Supplementary Information), the pro‐inflammatory factors TNF‐α and IL‐6 exhibited a significant decrease in expression following the use of ES in combination with HEPP. Conversely, the interleukin‐10 (IL‐10) showed a notable increase in expression (Figure S54, Supplementary Information), providing further evidence for the synergistic inflammatory modulation effect of HEPP and ES.

To validate the ability of HEPP to alleviate wound hypoxia, an intracellular hypoxia indicator, Ru(dpp), was employed to visualize cellular oxygenation in human umbilical vein endothelial cells (HUVEC) and NIH‐3T3 cells. In this experiment, HUVECs and NIH‐3T3 cells were initially subjected to hypoxic conditions and subsequently incubated with Ru(dpp) and treated with various materials. As illustrated in Figure 5I,J, both HUVECs and NIH‐3T3 cells exhibited considerable red fluorescence in the absence of materials treatment, indicating a relatively low level of cellular oxygenation following hypoxia treatment. In contrast, the intensity of cellular fluorescence was significantly diminished in the experimental group after treatment with PTPPG and HEPP, respectively, indicating adequate cellular oxygenation. To gain further insight into the mechanism of ES on cytokine‐induced macrophage polarization, quantitative reverse transcription‐polymerase chain reaction (q‐PCR) was employed to investigate alterations in messenger RNA (mRNA) expression (Table S1, Supplementary Information). The results demonstrated a synergistic decreased expression in TNF‐α and IL‐6, accompanied by a synergistic elevation in IL‐10 and transforming growth factor‐β (TGF‐β), as illustrated in Figure 5K–N. Additionally, the hypoxia‐inducible factor (HIF‐1α) exhibited downregulation after the treatment with ES and HEPP, indicating the alleviation of the hypoxic microenvironment. Additionally, Figure S55 (Supplementary Information) demonstrated that ES was effective in increasing the expression of CD31, a marker that signifies vascular endothelium and indicates angiogenesis.

### 2.6 RNA Sequencing Analysis of ES‐Assisted Inflammatory Regulation Using HEPP

**Figure 6:**
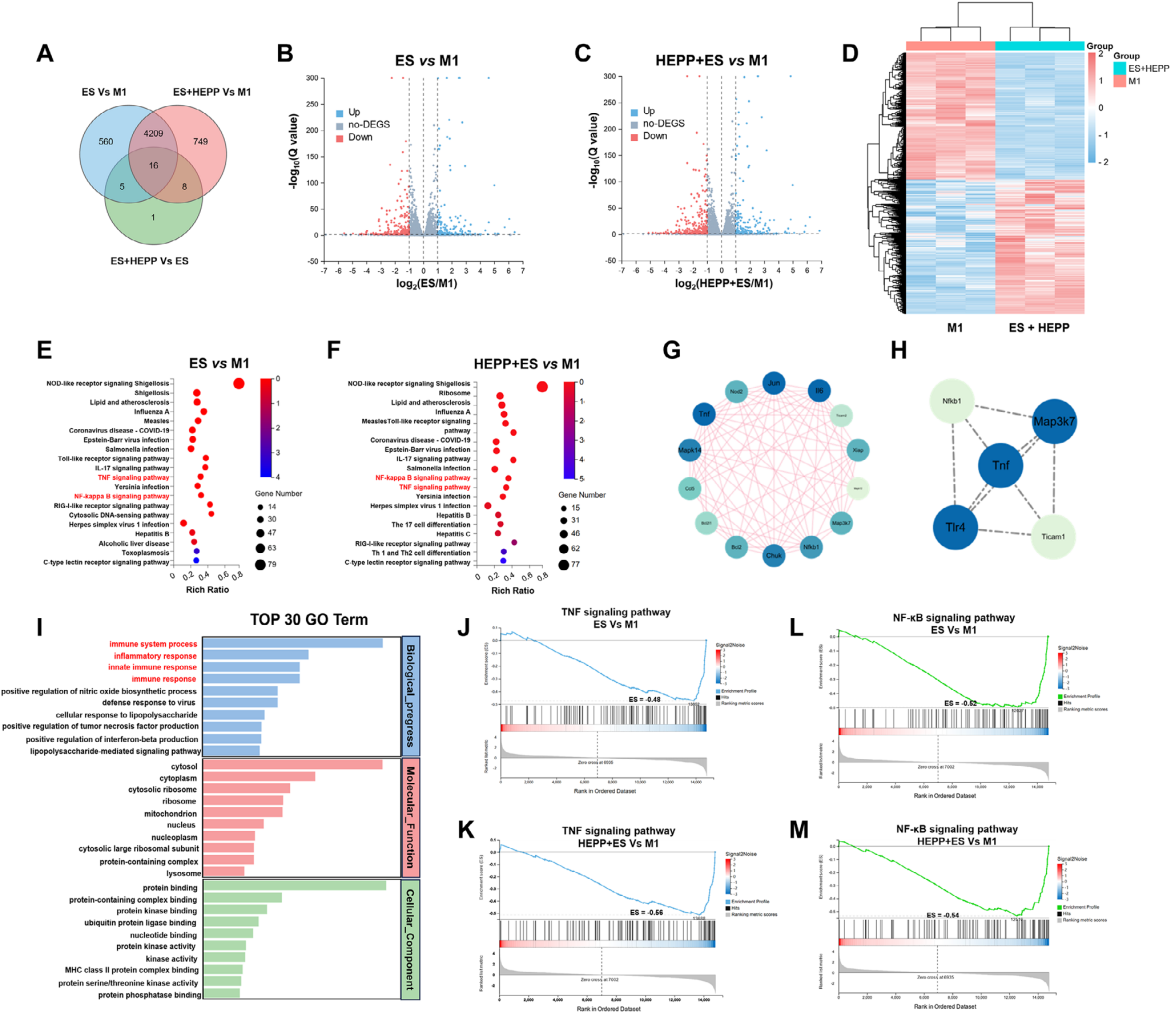
A) A Venn diagram illustrating the differential gene counts among the M1, ES, and HEPP+ES groups. Volcano plots illustrate differentially expressed genes in ES versus M1 group (B) and HEPP+ES versus M1 group (C), respectively (gray signifies nonsignificant genes; blue indicates upregulated genes; red stands for downregulated genes). D) Heatmap representation of the differentially expressed genes between M1 group and ES+HEPP group. Identification of top 20 signaling pathways using KEGG analysis in ES versus M1 (E) and HEPP+ES versus M1 group (F), respectively, enriched by genes expressed differently. PPI Networks enriched by genes expressed differently in TNF and NF‐*κ*B1 signaling pathways, contrasting ES group versus M1 group (G) and HEPP+ES group versus M1 group (H). I) GO enrichment analysis showing top 30 pathways involved. (J)–(M) GSEA analysis highlighting two key inflammatory pathways: TNF and NF‐*κ*B. Unless otherwise specified, all HEPP refer to gels with PDA@PPY doping amount of 10%.

To investigate the role of HEPP and ES in regulating inflammation, we aim to identify key genes involved during the treatment process. An RNA sequencing analysis was conducted to analyze RAW264.7 cells from the groups that were subjected to treatments with HEPP, ES, and a combination of both therapies. The transcriptional profiles of RAW264.7 cells that underwent M1 polarization (M1 group), received only ES treatment (ES group), and were treated with both HEPP and ES (HEPP+ES group) were compared using differential expression assessment, gene ontology (GO) enrichment analysis, Kyoto Encyclopedia of Genes and Genomes (KEGG) analysis. Principal component analysis revealed significant differences in gene expression among M1, ES, and HEPP + ES groups. The Venn diagrams showed that a total of 4238 genes with altered expression were shared among the M1, ES, and HEPP + ES groups. The ES group displayed a total of 560 unique genes, whereas the HEPP + ES group revealed 749 unique genes (**Figure** **6**A). As illustrated in Figure 6B, the volcano plot depicts the distribution of differential genes that exhibited notable changes in expression between the M1 and ES groups.^[^ ^44^ ^]^ After administering the ES treatment, a total of 115 significant genes exhibit varying levels of expression, with 52 genes showing a considerable increase and 63 genes reflecting a notable decrease. After the treatment with HEPP together with ES, a total of 159 key differential genes were found, with 57 genes exhibiting a significant increase in expression levels, whereas 102 genes experienced a considerable decrease (Figure 6C). The results demonstrated that the gene expression profiles of both the ES and HEPP + ES groups were markedly different from those obtained from the M1 group. Figure 6D presents a heatmap that illustrates the clustering of genes with differing expression levels, emphasizing the variations in specific gene activities between the M1 group and the HEPP + ES group. In this visualization, the red lines signify a rise in gene activity, whereas the blue lines indicate a decrease. To identify the signaling pathways associated with the immune response after treatment, KEGG analysis was conducted on the differential expressed genes in both ES group and the HEPP + ES group, respectively. This analysis aimed to ascertain the differentially expressed genes and reveal the potential pathways associated with inflammation. The results are presented in Figure 6E,F. Analysis of gene expression differences indicated a notable variation in the TNF and NF‐*κ*B signaling pathways, which were linked to inflammation in both the ES and HEPP+ES groups. In this context, the differential genes of the two groups underwent separate protein interaction analyses. As illustrated in Figure 6G, the TNF and IL‐6 genes were found to be dominant in the ES group compared to the M1 group. The findings indicate that these two genes play a crucial role in controlling inflammatory response. Similarly, a comparison of the HEPP + ES treated group with M1 group (Figure 6H) revealed that TNF was the dominant gene, with NF‐*κ*B1 gene regulation observed in both groups. Moreover, a GO enrichment analysis revealed that the discrepancies in gene expression between the M1 and HEPP + ES groups were closely linked to processes associated with the immune system response and inflammatory stimuli. To gain further insight into the potential synergistic effects of ES and HEPP, a gene set enrichment analysis (GSEA) was employed. The results demonstrated that the TNF pathway showed significant downregulation in both sets of comparisons (ES versus M1 and HEPP+ES versus M1). The degree of downregulation was more pronounced in HEPP+ES versus M1 (ES = − 0.56), suggesting that the TNF pathway experienced greater suppression in the HEPP+ES group, as illustrated in Figure 6J,K. Moreover, the NF‐*κ*B signaling pathway was also observed to be downregulated in both the ES group and the HEPP+ ES group (Figure 6L,M). The findings align with cellular‐level observations, showing that the HEPP + ES treatment group effectively suppressed the release of pro‐inflammatory cytokine IL‐6 through the downregulation of the signaling pathway involved in Th17 cell differentiation (Figure 5L and Figure S53, Supplementary Information). Consequently, the combination of protein–protein interaction (PPI) analysis and GSEA in the HEPP + ES group demonstrated that the treatment of HEPP together with ES primarily modulated inflammation by downregulating TNF and IL‐6 genes. Moreover, distinct analysis of the HEPP+ES and ES groups revealed that the combined effect of HEPP and ES was predominantly manifested in the downregulation of pivotal TNF genes. The gene sequencing results were found to be in high agreement with the q‐PCR results (Figure 5L and Figure S53, Supplementary Information). ES primarily reduced inflammation by inhibiting the TNF signaling pathway and IL‐6 production. The integration of HEPP with ES therapy demonstrated a collaborative effect, blocking the TNF signaling pathway and further reducing inflammation, which promoted the transition of macrophages from the M1 to M2 phenotype, thus speeding up the wound healing process.^[^ ^45^ ^]^

### 2.7 Therapeutic Effect of HEPP on Wounds

**Figure 7:**
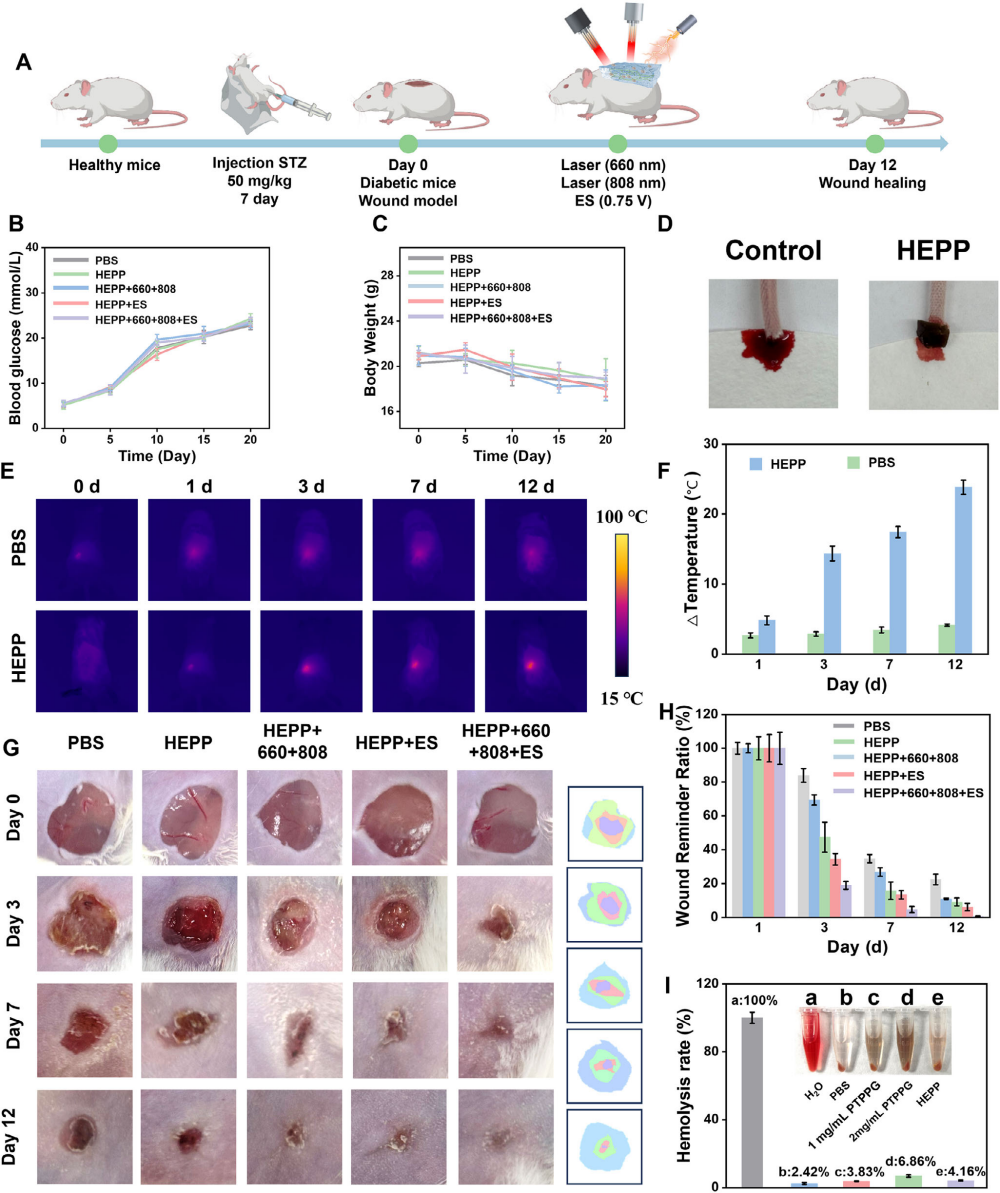
A) Diagram illustrating the creation of a mouse model for diabetic wound management. The blood Glu level (B) and body weight level (C) during the modeling and treatment process. D) The hemostatic properties of HEPP. E) Responsive photothermal imaging of the release of PTPPG during HEPP treatment. F) The temperature changes of the wound treated with and without HEPP. G) The images illustrating the progression of diabetic wound in mice that received different treatments. H) Changes in relative wound area in different groups. I) Hemolysis experiments of different materials.

We further applied HEPP in a diabetic mouse wound model to evaluate their efficacy in preventing bacterial infections, managing inflammation, and enhancing the wound‐healing process. A model of diabetes in mice was developed through the administration of streptozotocin, followed by the creation of circular wounds on their backs, which were subsequently treated using various methods (**Figure** **7**A). Mice were categorized into four treatment groups based on different experimental conditions: one group was treated with HEPP (HEPP group), another with HEPP in the presence of laser irradiation at wavelengths of 660 and 808 nm (HEPP + 660 + 808 group), a third group received HEPP together with ES (HEPP+ES group), and the last group was treated with HEPP, laser irradiation, and ES together (HEPP + 660 + 808 + ES group). During the modeling period, blood Glu levels were closely monitored, and diabetes was considered successfully modeled when fasting blood Glu levels reached 16.7 mM (Figure 7B).^[^ ^46^ ^]^ Meanwhile, continuous monitoring of body weight during modeling and treatment revealed a slight weight loss in the mice, which was associated with the effects of diabetes (Figure 7C). As illustrated in Figure 7D, HEPP showed promising hemostatic properties, demonstrating its potential use as a wound dressing. Following that, an investigation was conducted on the release of PTPPG at the wound location through exposure to an 808 nm laser, enabling the tracking of temperature fluctuations to assess the in vivo effectiveness of photothermal properties of PTPPG. As illustrated in Figure S56 (Supplementary Information), variations in temperature due to different concentrations of PTPPG convincingly demonstrated the viability of this approach. Furthermore, the gradual release of PTPPG from HEPP was confirmed by monitoring the thermal variations at the wound site for 12 days (Figure 7E). The group receiving HEPP treatment displayed a significant temperature variation when compared to the group treated with PBS over the span of 1–12 days, confirming the gradual release of PTPPG during the treatment process (Figure 7F). Mice receiving various treatment approaches showed faster wound recovery compared to those given PBS (Figure 7G,H). After 7 days, wounds treated with the HEPP still exhibited significant open area (26.8% of the wound area), comparable to those treated with PBS (34.7% of the wound area), suggesting that HEPP treatment alone had limited effectiveness in wound healing. In contrast, a notable decrease in the wound size was noted in other treatment groups. After 7 days, the HEPP + 660+ 808 + ES group showed a reduction in wound size to 4.6%, with nearly complete healing achieved by days 12, suggesting that the synergistic effect of multimodal treatment utilizing HEPP enhanced the healing process. The criteria for biocompatibility, as outlined by the International Organization for Standardization and the American Society for Materials and Testing, specify that materials with a hemolysis rate of 5% or less are compliant. As depicted in Figure 7I, the findings from the hemolytic experiment indicate that the hemolysis rates for 1 mg mL^−1^ PTPPG and HEPP were under 5%, satisfying international hemolysis test standards and demonstrating the favorable biocompatibility of HEPP.

**Figure 8:**
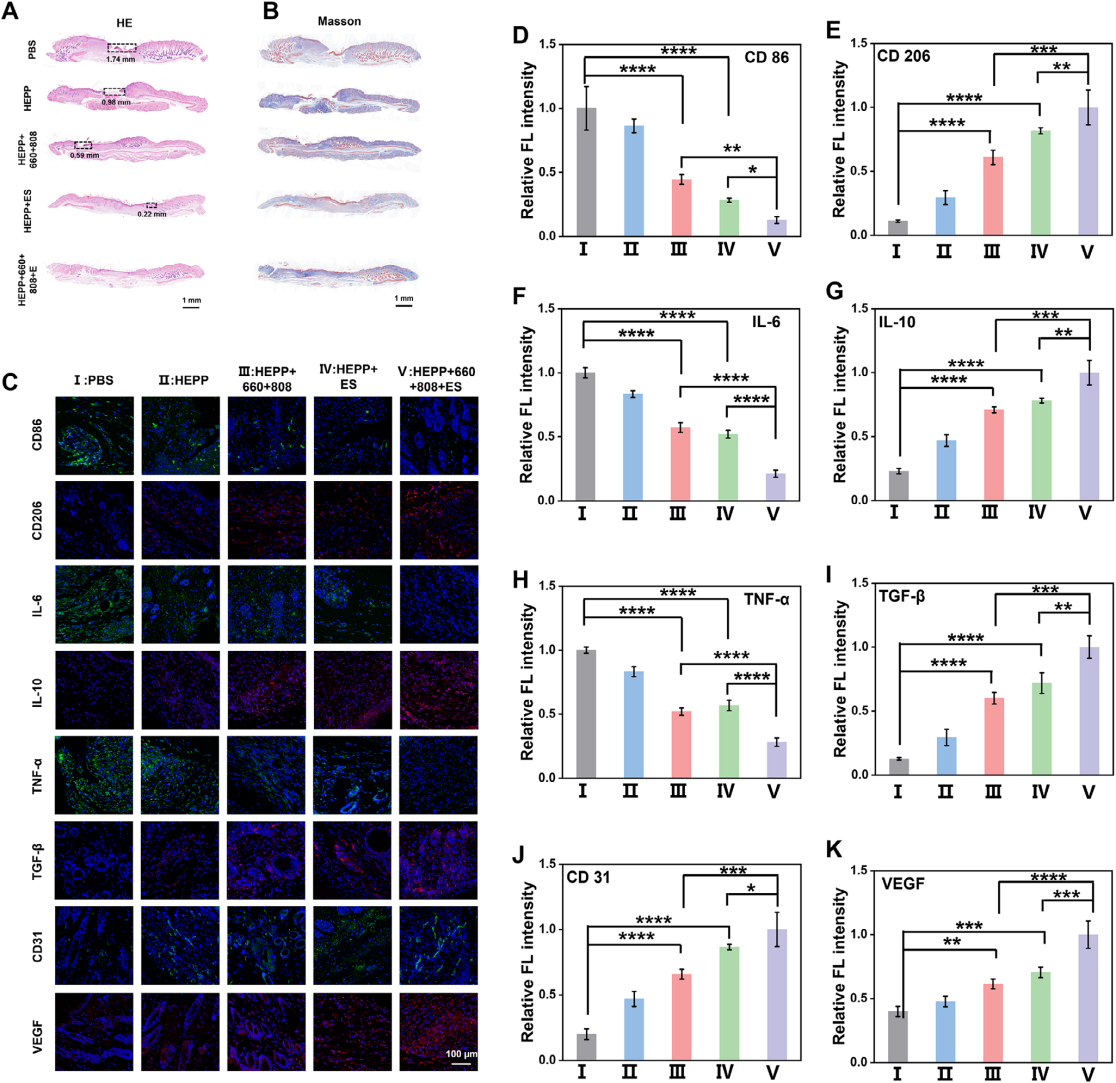
Histopathological examination using H&E staining (A) and Masson staining (B). C) Analysis of immunofluorescence for CD86, CD206, IL‐6, IL‐10, TNF‐α, TGF‐β, CD31, and VEGF in tissue sections obtained from the diabet wound site. D–K) Quantitative analysis of mean fluorescence intensities of CD86, CD206, IL‐6, IL‐10, TNF‐α, TGF‐β, CD31 and VEGF after various treatments (I–V represent PBS, HEPP, HEPP + 660 + 808, HEPP + ES, HEPP + 660 + 808 + ES treatment groups, respectively). Error bars represent the mean ± standard deviation for a sample size of 3. *p* values were calculated via one‐way ANOVA test. *p < 0.05, **p < 0.01, ***p < 0.001, ****p < 0.0001. Unless otherwise specified, all HEPP refer to gels with PDA@PPY doping amount of 10%.

In light of the encouraging results observed in diabetic mice associated with wound healing, we conducted a comprehensive investigation into the underlying mechanisms using histochemical staining and immunofluorescence techniques. The initial step involved histological analysis of the wounds after 12 days using H&E staining (**Figures** **8**A and S57). As can be seen, the HEPP + 660 + 808 + ES group exhibited notable healing, characterized by the regeneration of skin tissue with intact epithelial and dermal layers. Furthermore, an increase in the formation of new blood vessels and hair follicles was observed. Subsequently, Masson staining was employed to evaluate collagen deposition, which plays a pivotal role in skin regeneration.^[^ ^47^ ^]^ As illustrated in Figure 8B and Figure S57 (Supplementary Information), the HEPP + 660 + 808 + ES group exhibited densely arranged and well‐organized collagen fibers, indicative of augmented collagen deposition. H&E staining was also conducted on the main organs, including the heart, liver, spleen, lung, and kidney (Figure S58, Supplementary Information). No obvious inflammations were observed in any of the tested organs, indicating the toxicity of HEPP was negligible. These results demonstrate that HEPP has a favorable biosafety profile and can be utilized safely and effectively as a therapeutic agent for future applications. Moreover, immunofluorescence staining for multiple inflammatory markers was conducted to substantiate that ES in conjunction with HEPP facilitated the regulation of inflammation in mouse wounds (Figure 8C). Analyzing the expression of the CD86 and CD206 revealed that the PBS group exhibited the highest level of CD86 (Figure 8D), whereas greater CD206 expression compared to the other treatment groups was observed in the HEPP + 660 + 808 + ES group (Figure 8E), indicating that the phenotype of macrophages in diabetic wounds has changed. Moreover, the levels of inflammatory factors, such as TNF‐α and IL‐6, were markedly diminished (Figure 8F,H), while the levels of anti‐inflammatory factors, including IL‐10 and TGF‐β, were significantly elevated after the different treatments (Figure 8G,I). These data reinforce the idea that the inflammation at the wound site has been greatly alleviated. Moreover, the investigation of neovascularization in the upper wound tissues of mice was conducted using VEGF and CD31 staining. As illustrated in Figure 8J,K, the HEPP + 660 + 808 + ES group exhibited a more pronounced proliferation of neovessels compared to the other groups. This change is crucial for supplying essential nutrients and O_2_ to metabolically active wounds, leading to a notable decrease in HIF‐1α levels (Figure S59, Supplementary Information), and facilitating the formation of granulation tissue, which is consistent with those obtained from dorsal skin wounds in mice.

In conclusion, the employment of combination therapy facilitates the transformation of macrophages from the M1 to the M2 state, driven by the micro‐current generated through ES.^[^ ^48^ ^]^ This approach not only aids in mitigating chronic inflammation but also demonstrates that, when compared with standard care practices, the use of ES and gel‐assisted therapy can enhance the healing rate of diabetic wounds significantly. Furthermore, studies suggest that ES can direct epithelial cells and fibroblasts toward the wound site, enhance the secretion of growth factors such as VEGF, and foster angiogenesis and collagen synthesis.^[^ ^49^ ^]^ To facilitate the transition of this hydrogel from research to clinical practice, it is essential to investigate its long‐term effects, establish treatment standards, and collaborate with medical practitioners to ensure a balance between its various functions and simplicity, thereby effectively reducing overall expenses.

## 3 Conclusion

To sum up, the successful creation of a smart conductive hydrogel infused with PTPPG nanozymes and conductive nanowires (PDA@PPY) has been achieved. The restoration of the wound microenvironment was realized through the autonomous degradation of Glu facilitated by PTPPG nanozymes, the increase in O_2_ generation, and the adaptable antioxidant regulatory functions of HEPP. In addition, combining HEPP with ES demonstrated a notable impact on the regulation of the immune response. The study of transcriptomic profiles revealed that the combination of ES and HEPP led to a reduction in TNF signaling pathway activity and a shift in macrophage phenotype from M1 to M2, aiding in the alleviation of inflammation. The findings from both in vitro and in vivo studies revealed that the HEPP successfully facilitated self‐regulation of the wound microenvironment while simultaneously preventing bacterial infections. Simultaneously, the application of ES alleviated oxidative stress and inflammation in the affected area, fostered the growth and formation of blood vessels and accelerated the wound recovery. This study presents an innovative conductive hydrogel that incorporates self‐generated programmed antimicrobials, a synergistic approach to reducing inflammation by modulating its microenvironment and ES, along with a sophisticated response to pH variations, ROS, NIR, and electrical stimuli, making it a promising solution for treating inflammatory issues in diabetic wound management in the future.

## 4 Experimental Section

### Materials

All chemical reagents were used directly without other further purification. Titanocene dichloride (Cp_2_TiCl_2_), zirconyl chloride octahydrate (ZrOCl_2_ · 8H_2_O), tetrakis(4‐carboxyphenyl) porphyrin (TCPP), benzoic acid, glucose oxidase (GOx), chloroplatinic acid (H_2_PtCl_6_), sodium tetrachloropalladate (Na_2_PdCl_4_), ascorbic acid (AA), caffeic acid (CA), 3‐aminophenylboronic acid (3‐NH_2_‐PBA), ε‐polylysine (EPL), 2′,7′‐dichlorofluorescin diacetate (DCFH‐DA), sodium periodate (NaIO_4_), *N*‐hydroxysuccinimide (NHS), *N*‐(3‐dimethylaminopropyl)‐*N*′‐ethylcarbodiimide hydrochloride (EDC**·**HCl), 2‐(*N*‐morpholino) ethanesulfonic acid (MES), azino‐bis (3‐ethylbenzthiazoline‐6‐sulfonic acid) (ABTS), and dopamine hydrochloride (DA) were purchased from Adamas (shanghai, China). Hyaluronic acid (HA) and Pyrrole were acquired from Alladin (shanghai, China). Ammonium persulfate was obtained from Greagent (Shanghai, China). 1,1‐Diphenyl‐2‐picrylhydrazyl radical (DPPH) kit was purchased from Shanghai yuanye Bio‐Technology (Shanghai, China). Penicillin‐streptomycin and Trypsin‐EDTA (0.25%) was supplied by Gibco (Grand Island, NY, USA). Dulbecco's modified Eagle's medium (DMEM) and Roswell Park Memorial Institute (1640) were purchased from Biosharp (Shanghai, China). CD86 Polyclonal antibody, CD206 Monoclonal antibody, VEGF Monoclonal antibody, CD31 Polyclonal antibody and IL‐10 Monoclonal antibody were obtained from Proteintech (Wuhan, China). IL‐6 Monoclonal antibody was purchased from Abmart (Shanghai, China). Singlet Oxygen Sensor Green (SOSG), Calcein acetoxymethyl ester/propidium iodide (Calcein‐AM/PI) kit, TNF‐α Polyclonal antibody, and Streptozotocin (STZ) were acquired from Beyotime Biotechnology (Shanghai, China). All reagents were obtained without further purifications, and ultrapure water with a resistivity of 18.2 MΩ**·**cm was used during the whole experiment.

### Instrument

The morphological features of specimens were observed using a Hitachi SU8010 scanning electron microscope (SEM, Japan) at an accelerating voltage of 5 kV. For lyophilized hydrogels, the lyophilized hydrogel surface was sprayed with gold (EM ACE600, Germany) to increase the electrical conductivity. Transmission electron microscope (TEM) images of nanoparticles were taken on a Jeol JEM‐1230 instrument (Japan) operating at an accelerating voltage of 100 kV. The content of the Ti was measured using an Avio 200 inductively coupled plasma optical emission spectrometer (ICP‐OES, PerkinElmer Co., USA). Electron paramagnetic resonance (EPR) measurements were conducted via a EMXplus model spectrometer (Bruker, Germany) using 5,5‐dimethyl‐1‐pyrroline‐*N*‐oxide (DMPO) and 2,2,6,6‐tetramethylpiperidinooxy (TEMPO) as the radical spin‐trapping agent. X‐ray diffraction (XRD) analysis was employed to elucidate the crystal structures of the synthesized catalysts, utilizing a DX‐2700BH instrument (Haoyuan Instrument, China) with Cu Kα radiation over a 2θ range from 10 to 90 degrees. Thermogravimetric analysis (TGA) was carried out under an N_2_ atmosphere using TGA 4000 thermogravimetric analyzer (PerkinElmer Co., USA) with a ramp rate of 10 °C min^−1^. The proton–nuclear magnetic resonance (^1^H NMR) spectra of the specimens were gathered via a Quantum‐I NMR device (China) running at a frequency of 400 MHz using deuterium oxide as the solvent. The surface functional group of materials was assessed by a Bruker Tensor II Fourier transform infrared (FT‐IR) instrument (Germany). All specimens were scanned in the 400–4000 cm^−1^ range with an interval of 2 cm^−1^. X‐ray photoelectron spectroscopy (XPS) of nanoparticles was measured using an ESCALAB 250XI spectrometer (Thermo Fisher, Waltham, USA) with the aid of Al Kα radiation. The rheological characteristics of the fabricated HEPP were studied at room temperature using a stress‐controlled rheometer (DHR‐2, TA, USA). Briefly speaking, the hydrogel precursor was injected into a circular plastic mold with a diameter of 25 mm and a height of 1 mm to produce specimens that matched the rheometer shear disc. In the first stage, a strain sweep was conducted on cured samples at 25 °C, followed by escalating the oscillatory strain from 0.1% to 1000% to ascertain the linear viscoelastic region. The second step involved frequency sweep tests at 25 °C to investigate the correlation between frequency (which increased from 0.1 to 10 Hz) and modulus (comprising both storage modulus *G*′ and loss modulus *G*″). Finally, dynamic stepped strain amplitude experiments were conducted to examine the hydrogel's self‐healing properties, with oscillatory strains alternating from 1% to 250%. The conductivity of hydrogels was detected by a ST2242 hand‐held alternating current four‐probe tester (Suzhou Jinge Electronic Technology Co., Ltd, China).

### Synthesis of PCN‐224

PCN‐224 was synthesized via the solvothermal method. In brief, 60 mg of ZrOCl_2_·8H_2_O, 20 mg of tetrakis (4‐carboxyphenyl) porphyrin (TCPP) and 560 mg of benzoic acid were dissolved in 20 mL DMF. After ultrasonication for 15 min, the mixture was transferred into a 50 mL flask to reflux at 90 °C for 5 h. The precipitate was collected after centrifugation at 15000 rpm for 15 min. After that, the precipitate was washed three times with DMF and ethanol. Finally, the material was dissolved in ultrapure water and stored at 4 °C refrigerator.

### Synthesis of PCN‐224(Ti)

PCN‐224(Ti) was synthesized via the cation exchange method. Briefly, 80 mg of PCN‐224 and 80 mg of TiCp_2_Cl_2_ were dissolved in 12 mL anhydrous DMF. The mixed solution underwent sonication for a few minutes to get a well‐dispersed suspension. The mixed solution was transferred into a 20 mL Teflon‐lined stainless‐steel autoclave and reacted at 120 °C for 48 h. After cooling, the products were collected by centrifugation after sequential washing with DMF and ethanol and dried at 80 °C oven for further usage.

### Synthesis of PTPP

PTPP nanoparticles were created using the in situ generation method. In simple terms, 140 µL H_2_PtCl_6_ (20 mM) and 60 µL Na_2_PdCl_4_ (20 mM) were added into a 1000 µL solution containing PCN‐224(Ti) (1 mg mL^−1^), followed by the addition of 40 µL ascorbic acid (AA, 20 mg mL^−1^) into the mixed solution. Subsequently, the mixed solution was immediately transferred to a water bath at 65 °C for 10 min. The black product was collected by centrifugation at 12 000 rpm for 5 min, followed by three rinses with ultrapure water, and then it was dispersed in ultrapure water.

### Synthesis of PTPPG

PTPP (10 mg) and 1‐(3‐dimethylaminopropyl)‐3‐ethylcarbodiimide (EDC, 5 mg) were dissolved in 10 mL of ultrapure water and stirred at 37 °C for 30 min. After that, glucose oxidase (GOx, 5 mg) was added and stirred overnight at 37 °C. The precipitation was collected by centrifugation, washed with ultrapure water three times and stored at 4 °C.

### Synthesis of Aldehyde Form of HA (OHA)

Briefly, 1.0 g of hyaluronic acid (HA) was dissolved in 200 mL of ultrapure water, then followed by the addition of 15 mL sodium periodate (5.3 mM) dropwise into the hyaluronic acid solution (5 mg mL^−1^). The reaction continued for 2 h and was terminated completely by the addition of 1m L ethylene glycol for another 1 h. After that, the resulting solution was dialyzed for 72 h (MWCO: 3500 Da) and freeze‐dried at –60 °C to obtain dry product.

### OHA Branched 3‐Aminophenyl Boronic Acid (OHA‐PBA)

A total of 0.9 g OHA (0.9 g) was dissolved in 40 mL of ultrapure water with the addition of 270 mg 3‐aminophenyl boronic acid (3‐NH_2_‐PBA). The mixed solution was stirred for 12 h at room temperature. After that, the resulting solution was dialyzed for 72 h (MWCO: 3500 Da) to remove unreacted monomers. Finally, the solid was obtained by freeze‐drying at –60 °C.

### ε‐Polylysine Branched Caffeic Acid (EC)

A total 1 g of ε‐polylysine (EPL) was dissolved in a MES buffered solution (10 mM, 50 mL, pH 5.0). After that, 1.1 g of EDC and 0.66 g of NHS were added to activate the carboxy group of caffeic acid for 30 min in an ice water bath. After that, 50 mL ε−polylysine solution (20 mg mL^−1^) was added and reacted at room temperature for 24 h under N_2_ atmosphere. Finally, the resulting solution was dialyzed for 48 h using a dialysis bag with a MWCO of 3500 Da. The as‐prepared solution was freeze‐dried at –60 °C to obtain the dry product.

### Synthesis of PDA@PPY NWs

Briefly, 0.48 mL of pyrrole monomer and 0.2 g of dopamine hydrochloride were dissolved in 100 mL Tris‐HCl buffered solution (pH 8.5, 100 mM) and cooled in an ice water bath. After that, 15 mL aqueous solution containing 2 g of ammonium persulfate was added and stirred at 8 °C for another 18 h. After washing with ultrapure water several times, the product was collected by centrifugation. Finally, the washed precipitate was lyophilized to obtain PDA@PPY powder.

### Synthesis of HEPP

First, 10 mL EC solution (5 mg mL^−1^) was added into a 17 mL solution containing 5 mg mL^−1^ OHA‐PBA, PTPPG (1 mg mL^−1^) and 1 mg mL^−1^ PDA@PPY and the mixture was quickly stirred to form HEPP. We obtained different hydrogels using the following recipes: HE was a gel formed by cross‐linking OHA‐PBA with EC monomer; 10% mass fraction of PDA@PPY was doped into HE to obtain HEP; the HEP gels (100 mg) doped with 0, 5, 10, and 20 mg of PDA@PPY include 10 mg PTPPG were named as 0% HEPP, 5% HEPP, 10% HEPP, and 20% HEPP, respectively. Unless otherwise specified, all HEPP refer to gels with PDA@PPY doping amount of 10%.

### Macrophage Phenotype Modulation Experiments

Different materials were inoculated onto the bottom of confocal culture dishes, which were sterilized with 75% ethanol and UV light. The 1 × 10^5^ cells of RAW264.7 macrophages were inoculated in a 24‐well plate with confocal climbing sheets and incubated at 37 °C overnight for cell proliferation. After that, M1 polarization of macrophages was stimulated within 24 h of incubation with LPS (200 ng mL^−1^). After 24 h of treatment with different materials or ways (HEP, PTPPG, HEPP, HEP + ES, PTPPG + ES, HEPP + ES), cells were washed with PBS three times, fixed with 4% paraformaldehyde, permeabilized using 0.5% Triton X‐100 and blocked with 3% BSA, successively. Then, rabbit polyclonal antibodies targeted CD86 and CD206 were incubated with RAW264.7 overnight, and then incubated with Alexa Fluor 488 goat anti‐rabbit IgG secondary antibody (1:200) and Alexa Fluor 594 goat anti‐rabbit IgG secondary antibody (1:200) for 60 min. Hoechst 33342 was used to stain the nucleus and cell imaging was achieved via laser scanning confocal microscopy. IL‐6, TNF‐α, IL‐10, CD31 were also stained through the same modification method. The number of CD206 and CD86 positive cells was counted by Image J.

### Electrical Stimulation Modulates Macrophage Phenotype

The RAW264.7 macrophages (1 × 10^5^ cells) were inoculated in a 12‐well plate with confocal climbing sheets and incubated at 37 °C overnight for cell proliferation and adhesion. After that, M1 polarization of macrophages was stimulated within 24 h of incubation with LPS (200 ng mL^−1^). After 24 h of treatment, the macrophages turned to M1, and then HEPP was added and incubated at 37 °C for 2 h. After that, the cells in the 12‐well plate were stimulated with 0.75 V alternating current for 5 min. Then the cells were incubated at 37 °C for 2 h and the fresh medium was added. After 24 h, the phenotype was determined by immunofluorescence staining.

### qRT‐PCR Experiment

To further explore the effects of HEPP stimulation and ES to inflammation, RAW264.7 cells were cultured in vitro and stimulated with LPS (200 mg mL^−1^) for 24 h under hypoxic conditions (1% O_2_, 5% CO_2_, and 94% N_2_). The cells were treated with PBS, ES and HEPP+ES, respectively, for 5 min and incubated for 24 h. After cells were collected, total RNA was isolated using the RNA‐easy Isolation reagent and reverse‐transcribed using a HiScript III RT SuperMix kit following the instructional manual. qRT‒PCR analysis was performed using ChamQ SYBR qPCR Master Mix and a Roche Real‐Time PCR Detection System. mRNA expression level and macrophage markers were detected to monitor changes in the inflammatory state of macrophages.

### Diabetic Wound Modeling

The SPF male BALB/c mice used in this experiment were purchased from Hunan Silaikejingda Experimental Animal Co., Ltd. All mice were anesthetized during animal model modeling and treatment. Healthy male BALB/c mice (6 weeks old, weighing about 20 g) were utilized to create a model of type I diabetes by low‐dose continuous multiple injections of streptozotocin (STZ), injected with 50 mg kg^−1^ for 7 consecutive days. After 12 h of fasting, the mice were administered an intraperitoneal injection of 10 mg mL^−1^ streptozotocin. Every 3 days, the tail was cut to take blood, and the blood glucose of the mice was detected by a blood glucose meter. The fasting blood glucose of the mice was ≥ 11.1 mM, and the nonfasting blood glucose was ≥16.7 mM, indicating that the diabetic mouse model was successfully constructed.

After 7 days, blood glucose levels were checked by random sampling from the tail vein. The diabetes model was deemed effectively set up when the randomly measured blood glucose level surpassed 16.7 mM. Upon completion of the experiment, all the diabetic rats were euthanized by an overdose injection of sodium pentobarbital.

The mice that successfully constructed the diabetic model were taken as the experimental subjects to construct the diabetic wound model. The mice were intraperitoneally injected with 200 µL of pentobarbital sodium solution at a concentration of 3 mg mL^−1^. After anesthesia, the razor and hair removal cream were used to remove the hair on the back of the mice. The back skin of mice was disinfected and a skin biopsy punch with a diameter of 8 mm was used to make wounds. Then sterile dressings were used to cover the wound site. Diabetic mice with constructed wounds were randomly divided into groups, with 5 mice in each group, to carry out follow‐up treatment. Ultimately, the assessment and imaging of the wound recovery process took place on 0, 3, 7, and 12 days.

### Hemostasis Experiments

The hemostatic properties of the constructed hydrogels were evaluated using a tail hemostasis model. The tail was cut at the lower third of the mice's tail with surgical scissors. After 15 s, the wounds were covered with HEPP and photographed. The blood loss was recorded.

### Wound Healing Mechanism Studies

Mice were euthanized at predetermined time points and dissected to obtain regenerated dorsal skin and major organs (heart, liver, spleen, lung, and kidney). The excised skin tissues and organs were placed in a 4% paraformaldehyde solution for fixation. After 24 h, the fixed tissue sections were stained with hematoxylin and eosin (H&E), Masson. Immunofluorescence staining of tumor necrosis factor‐alpha (TNF‐α), Interleukin‐6 (IL‐6), Interleukin‐10 (IL‐10), transforming growth factor‐β (TGF‐β), Hypoxia‐Inducible Factor‐1α (HIF‐1α), vascular endothelial growth factor (VEGF), platelet endothelial cell adhesion molecule‐1 (CD31), CD86 (M1 marker), and CD206 (M2 marker) was conducted to validate the changes in inflammation.

### Statistical Analysis

Statistics Analysis System 9.4 (SAS, SAS Institute Inc, USA) was employed for statistical analysis. Data were presented as mean ± SD from a minimum of three separate biological replicates unless otherwise specified in the figure caption. The analysis of the data involved employing a two‐sided Student's *t*‐test for comparisons between two groups. One‐way or two‐way analysis of variance (ANOVA) was used for multiple comparisons when comparing more than two groups. The difference was classified as significant if the *p*‐value was 0.05 or lower (*p < 0.05, **p < 0.01, ***p < 0.001, ****p < 0.0001).

### Ethics

In this study, BALB/c mice (male, 5 weeks) were used to establish diabetic wound models. All animal experiments adhered to the Animal Ethics and Welfare Guidelines of the Chinese Ministry of Public Health and were approved by Central South University Laboratory Animal Welfare Ethics Committee with the accreditation number: CSU‐2024‐0235.

## Conflict of Interest

The authors declare no conflict of interest.

## Supporting information



Supporting Information

## Data Availability

The data that support the findings of this study are available from the corresponding author upon reasonable request.
