# Supplementary material for: An Intelligent and Conductive Hydrogel with Multiresponsive and ROS Scavenging Properties for Infection Prevention and Anti‐Inflammatory Treatment Assisted by Electrical Stimulation for Diabetic Wound
Source: Adv Sci (Weinh). 2025 May 8;12(23):2500696. doi: 10.1002/advs.202500696 (PMC12199324; doi:10.1002/advs.202500696)
Supplement: Supplementary file 1 — Supporting Information [file ADVS-12-2500696-s001.docx]

Supplementary Information

**An Intelligent and Conductive Hydrogel with Multi-Responsive and ROS Scavenging Properties for Infection Prevention and Anti-inflammatory Treatment Assisted by Electrical Stimulation for Diabetic Wound**

*Tao Zhang, Zongwu Meng, Haoyu Yu, Ping Ding^*^and Tianhan Kai^*^*

T. Zhang, Z. Meng, H. Yu, P. Ding, T. Kai

Xiangya School of Public Health

Central South University

Changsha, Hunan 410013, China
E-mail: [pingshui@csu.edu.cn](mailto:pingshui@csu.edu.cn), [th_kai@csu.edu.cn](mailto:th_kai@csu.edu.cn)

T. Kai

Furong Laboratory

Central South University

Changsha, Hunan 410008, China

T. Zhang, Z. Meng, H. Yu, P. Ding, T. Kai

Hunan Provincial Key Laboratory of Clinical Epidemiology

Central South University

Changsha, Hunan 410078, China

**SUPPLEMENTAL MATERIALS AND METHODS**

**Peroxidase (POD) Activity**

3,3',5,5'-Tetramethylbenzidine (TMB) was used as a chromogenic agent to evaluate the POD activity of PTPPG under acidic conditions. 10 μL (1 mg/mL) PTPPG, 100 μL TMB solution (10 mM) and 100 μL H_2_O_2_ (100 mM), 1000 μL acetate buffered solution (10 mM, pH 4.5) were mixed, followed by the addition of a precise volume of water to ensure the overall volume equaled 2 mL. The POD activity was evaluated by collecting the absorbance at 652 nm by ultraviolet–visible (UV–vis) spectroscopy. All steps were performed under N_2_ or low oxygen conditions to prevent interference with oxidase activity of PTPPG. Under the same conditions, PTPPG concentration (final concentration 0~10 µg/mL) and H_2_O_2_ concentration (final concentration 0~5 mM) were adjusted to verify the dependence of peroxidase activity.

1,2-diaminobenzene (OPD) was used as a chromogenic agent to evaluate the POD activity of PTPPG under neutral or weakly alkaline conditions. 10 μL (1 mg/mL) PTPPG, 100 μL OPD solution (10 mM) and 100 μL H_2_O_2_ (100 mM), 1000 μL acetate buffer (10 mM, pH 7.4) were mixed. The volume was then brought up to 2 mL using ultrapure water. POD activity was evaluated by measuring the absorbance at 415 nm using UV–vis spectroscopy. All steps were performed under N_2_ or low oxygen conditions to prevent interference with oxidase activity of PTPPG. PTPPG concentration (final concentration 0~10 μg/mL) and H_2_O_2_ concentration (final concentration 0~5 mM) were adjusted to verify the dependence of the POD activity.

**Oxidase (OXD) Activity**

TMB was used as a chromogenic agent to evaluate the activity of OXD activity under acidic conditions. 10 μL (1 mg/mL) PTPPG, 100 μL TMB solution (10 mM), 1000 μL acetate buffer (10 mM, pH 4.5) were mixed and the final volume of 2 mL by adding 890 μL ultrapure water. The absorbance at 652 nm was measured using UV–vis spectroscopy to monitor the oxidation degree of TMB. Unless otherwise stated, the experiment was carried out under the atmosphere condition.

OPD was used to evaluate OXD activity under neutral or weakly alkaline conditions. 10 μL (1 mg/mL) PTPPG, 100 μL OPD solution (10 mM), and 1000 μL acetate buffer (10 mM, pH 7.4) were mixed, followed by the addition of a precise volume of water to ensure the overall volume equaled 2 mL. The absorbance at 415 nm was measured to determine the degree of OPD oxidation under the atmosphere condition.

**Catalase (CAT) activity**

The generation of O_2_ in vitro was detected by Ru(dpp), which would emit red fluorescence in a hypoxic environment. In a CAT assay, 10 μL (1 mg/mL) of PTPPG, 100 μL of H_2_O_2_ (10 mM), and 100 μL of Ru(dpp) (5 mM) were mixed and made up to a final volume of 2 mL with ultrapure water. The mixture was reacted at room temperature for 5 min under N_2_ atmosphere. The fluorescence at 620 nm was collected at the excitation wavelength of 488 nm to verify that PTPPG catalyzes the decomposition of H_2_O_2_ and produces oxygen. At the same time, the oxygen content in the solution before and after the reaction was calculated based on the change in fluorescence intensities. The calculation formula is as follows:

O_2_ Relative content = [(F_0_-F_t_)/F_0_] × 100%.

F_0_ represents the initial fluorescence intensity of the reaction, F_t_ represents the fluorescence intensity after the reaction.

**Glucose oxidase (GOx) activity of PTPPG**

To verify the successful loading of glucose oxidase (GOx) on PTPPG and monitor the pH changes in the real wound environment. TMB (pH 4.5) and OPD (pH 7.4) were used as chromogenic substrates to explore whether the enzymatic cascade reaction was feasible.

TMB validation: 10 μL PTPPG (1mg/mL), 100 μL TMB (10 mM), 100 μL glucose solution (100 mM) and 1000 μL acetate buffer (10 mM, pH 4.5) were mixed and the final volume of 2 mL by adding 790 μL ultrapure water. The mixture was incubated at 37 °C for 30 min. PTPPG concentration (final concentration 0~10 µg/mL) and glucose concentration (final concentration 0~5 mM) were adjusted to verify the concentration dependence of the enzymatic cascade reaction.

OPD verification: 10 μL PTPPG solution, 100 μL OPD solution (10 mM) and 100 μL glucose solution were added into a 1000 μL acetate buffered solution (10 mM, pH 7.4) and the final volume of 2 mL by adding 790 μL ultrapure water. The mixture solution was incubated at 37 °C for 30 min. PTPPG concentration (final concentration 0~10 μg/mL) and glucose concentration (final concentration 0~5 mM) were adjusted to verify the dependence of the enzymatic cascade reaction.

**Hydrogen peroxide (H_2_O_2_) generation by PTPPG**

To demonstrate that TMB and OPD coloration in the cascade reaction are originated by glucose decomposition. The generation of H_2_O_2_ in the cascade reaction is verified via the change of the UV-Vis spectrum of potassium permanganate (KMnO_4_). 10 μL PTPPG solution, 100 μL glucose solution and a certain volume of KMnO_4_ solution (4 mg/mL) were mixed and ensure the final volume is 2 mL by adding ultrapure water. The solution was centrifuged after reaction 30 min, and the absorbance of supernatants was measured. By varying the glucose and PTPPG concentration, the amount of H_2_O_2_ generated by PTPPG was also evaluated.

**Conductivity Test**

Hydrogels with different doping agents were created, each measuring a uniform size of 2 × 2 cm and thickness of 2 mm. The electrical conductance of the hydrogels was assessed through the four-probe testing technique. The measurement of the potential difference (V) between the electrodes inside was conducted by applying alternating current (I) to the electrodes located outside. The conductivities of the hydrogel were determined using the following equation:

Conductivity = I / 2πSV

Where S denotes the value of electrode spacing (m).

**In vitro photodynamic analysis**

To assess the PDT properties of the developed hydrogels, a singlet oxygen (^1^O_2_) assay was employed using Singlet Oxygen Sensor Green (SOSG) as the indicator agent. 10 μL of PCN-224, PCN-224(Ti), PTPP and PTPPG (1mg/mL) was diluted into a 2 mL solution containing SOSG (5 μM), respectively. The fluorescence intensity of the solution at 525 nm was measured after 660 nm laser irradiation (1W) for 5 min. The laser power and irradiation time were optimized to verify the material properties toward PDT.

**In vitro Photothermal analysis**

The photothermal properties of TPPPG, HEP, HEPP were evaluated via an infrared camera. The real-time temperatures and corresponding thermal images of a 500 µL solution containing 1mg/mL PTPPG, HEP, and 10% HEPP, respectively, were collected after the irradiation using an 808 nm laser with different power densities (0, 0.3, 0.6, 0.9 and 1.2 W) for 5 min. Photothermal conversion efficiencies were obtained by fitting the experimental photothermal cycles of the PTPPG hydrogels that were exposed to 808 nm laser irradiation (0.9 W), with a heating and cooling interval of 10 minutes per cycle. The photothermal stability of the PTPPG was assessed by repeatedly irradiating the sample for 4 cycles.

**Effects of PTT on POD activities of PTPPG**

To explore the effects of Photothermal on POD activities of PTPPG and enzymatic cascade reaction, the absorbance of oxTMB and oxOPD were monitored at 25 °C and 50 °C, respectively. The following experimental steps were as described above.

**Water uptake and retention**

The water uptake behavior of HEP and HEPP was assessed by the weighing method. Typically, when the hydrogels were lyophilized, it was weighed precisely (denoted as W_0_) and then submerged in a sample volume of buffer at 37 °C. The HEP and HEPP were retrieved from the buffer solution after a certain period. After removing extra liquid droplets from the hydrogel surface using moistened filter paper, the weight of the hydrogel was recorded as W_A_. This process was repeated until the hydrogels were saturated with water. The water uptake ratio was determined using the following formula: Water Uptake (g/g) = (W_A_ – W_0_)/W_0_.

The water retention capacities of HEP and HEPP hydrogels were also demonstrated by the weighing method. The gels were placed in a fume hood at 37 ℃ until water uptake equilibrium was obtained. The initial weight and the weight during the heat were denoted as W_0_ and W_B_, respectively. Water retention is determined by applying the following equation: Water retention (%) = W_B_/W_0_ × 100 %.

**pH and ROS responsiveness of HE**

The introduction of borated esters and Schiff base bonds into HE gels enabled the gel to be responsive to pH and ROS. The responsiveness of the gel was investigated by placing it in the Tris-HCl buffered solution (pH 4) with or without 100 mM H_2_O_2_. The gel fluidity change was recorded during the whole process.

**Release rate of PTPPG**

We quantify the release of PTPPG through the UV characteristic peak of TCPP in the solution. HEPP was placed in acetate buffer solution at pH 4.0, 6.0, and 7.4, respectively, to monitor the release of PTPPG within 60 hours in the presence and absence of different concentrations of H_2_O_2_ or glucose.

**ROS scavenging**

The ability of HEP and HEPP to scavenge free radicals was tested via 1,1-diphenyl-2-trinitrophenylhydrazine (DPPH) radical scavenging assay. Briefly, the samples were added into an ethanol solution containing 0.1 mM DPPH and kept at 37 °C in a dark environment for 30 minutes. The absorbance of each sample at 517 nm was then measured using a UV–vis spectrophotometer. The formula used to calculate the scavenging rate was:

Scavenging Rate = (1 – A_0_/A_C_) × 100%

Where A_C_ signifies the initial absorbance of the DPPH solution, and A_0_ signifies the absorbance of DPPH after a 30-minute interaction with the sample.

The capacity of the nano-materials to neutralize ROS was evaluated using the total antioxidant capacity assay kit [2,2’-azino-bis (3-ethylbenzthiazoline-6-sulfonic acid), ABTS]. The HEP and HEPP were added into a solution containing 0.1 mM ABTS, respectively, and reacted at 37 °C for 6 minutes. The absorbance was measured at 414 nm using a UV-Vis spectrophotometer. The formula for calculating the scavenging rate was:

Scavenging Rate = (1 – A_D_/A_E_) × 100%

Where A_0_ represents the initial absorbance of the ABTS working solution, and A_E_ represents the absorbance of the ABTS after a 5-minute interaction with the sample.

**In Vitro Anti-bactericidal activity**

Staphylococcus aureus (*SA*), Methicillin-resistant Staphylococcus aureus (*MRSA*), Pseudomonas aeruginosa (*PA*), and pan-drug resistant Pseudomonas aeruginosa (*PDR-PA*) were selected to evaluate the antibacterial activity of PTPPG. The desired bacteria were cultured in Luria-Bertani (LB) medium at 37 °C with continuous shaking, and the bacterial concentrations were determined via a turbidity meter.

1) antibacterial PDT:

The required bacteria were shaken in LB medium at 37 ℃. Diluted bacteria suspensions (50 μL, 2× 10^8^ CFU/mL) were pipetted into a 96-well plate, followed by the addition of 50 μL nanomaterials (PBS, TCPP, PCN-224, PT, PTPP, PTPPG). The bacterial suspension was incubated in a bacterial incubator at 37 °C for 12 hours after 660 nm laser irradiation for various times at different power densities and detected by a microplate reader at 600 nm.

2) PTT antibacterial:

The prepared bacteria suspensions (50 μL, final concentration of 1 × 10^8^ CFU/mL) were mixed with 10% HEPP and added into a 96-well plate, followed by the treatment with 808 nm laser irradiation for different time and different power densities, and incubated in a bacterial incubator at 37 °C for 12 hours. The optical density of the bacterial suspension at 600 nm was detected by a microplate reader. There were three parallel comparison groups in each experiment.

3) CAT antibacterial:

The diluted bacteria suspensions (50 μL, final concentration of 1 × 10^8^ CFU/mL) were pipetted to a 96-well plate and mixed with 50 μL 10% HEPP. After the suspension was treated with 2 mM H_2_O_2_, it was incubated in a bacterial incubator at 37 °C for 12 hours and detected by a microplate reader at 600 nm.

The conventional plate counting method was adopted to assess changes in the number of colonies after PTPPG treatment. 50 µL of diluted bacteria suspensions (final concentration is 5 × 10^5^ CFU/mL) were cultured with LB medium with or without nanomaterials. The mixtures were smeared onto LB agar plates and exposed under laser irradiation for different times to ensure appropriate light-dependent bactericide times. The proportion of bacteria that were eliminated was determined by enumerating the bacterial count at intervals of 0, 10, and 30 minutes, with the results being divided by the corresponding control values.

Firstly, four bacteria strains (*SA*, *MRSA*, *PA*, *PDR-PA*) were cultured in LB at 37 °C for 12 hours in a bacterial incubator. After washing with PBS three times and collecting by centrifugation, the bacteria were suspended in sterile PBS (pH 7.4) and adjusted to 1 × 10^8^ CFU/mL. After different treatments, bacterial live-dead dyes kit (Syto9/PI) were used for dyeing. Confocal laser Scanning microscope (CLSM) was used to determine whether the bacteria were alive (syto9, green fluorescence) or dead (PI, red fluorescence) after stained with Syto9 and PI for 15 min.

**SEM of bacterial and Live/Death staining test**

Firstly, diluted bacteria suspensions were cultured in LB overnight at 37 °C in an orbital incubator. After being cleaned twice by PBS and gathered through centrifugation, bacteria were suspended in sterilized PBS (pH 7.4) and adjusted to 5 × 10^5^ CFU/mL. To determine the bactericidal effect, 100 µL of the above bacterial suspension was treated with different materials or methods. After that, bacteria were cultured at 37 °C in a constant temperature incubator for another 2 hours. Then, the bacteria suspension was evenly coated on the agar plate to determine the bacteria concentration by counting the bacterial colony after 12 hours cultivation. Then, the morphology of bacteria after being treated with the hydrogel was observed by SEM. The bacteria in different group were fixed on silicon slices with 2.5% glutaraldehyde solution, rinsed with PBS, dehydrated with ethanol, and dried under vacuum, successively. Finally, the morphology of bacteria was observed by scanning electron microscopy.

**CCK-8 assay**

The cytotoxicity of PTPPG, HEP and HEPP were evaluated via the CCK-8 assay. In brief, Mouse embryonic fibroblasts cells (NIH-3T3), Human Umbilical Vein Endothelial Cells (HUVEC) and macrophages cells (RAW 264.7) were seeded into 96-well plates (with a seeding density of 1 × 10^5^ cells per well) and then incubated at 37 °C for 24 hours. The cell culture medium was removed from the 96-well plates, followed by the addition of sterilized materials (PTPPG, HEP and HEPP) and re-immersed in the culture medium for 24 h. After incubating for 24 hours, the medium in the 96-well plate was carefully removed and 150 µL of fresh cell culture medium comprising 10% CCK-8 was incorporated into each well. After continuous incubation for 2 hours, the absorbance of the solution at 450 nm was determined using a microplate reader.

**Cell live/dead staining test**

Briefly, NIH-3T3, HUVEC and RAW264.7 cells were inoculated in confocal discs at a density of 5 × 10^5^ cells per well and incubated for 12 hours. After that, PTPPG, PTPPG and HEPP samples were introduced into the plates and incubated for another 24 hours. Live/dead cells were stained using a Calcein-AM/PI staining kit according to the instructions of manufacturer. The fluorescence of the cells was monitored using a CLSM.

**Blood Compatibility**

The fresh blood of rats was collected and put into an anticoagulant tube containing heparin sodium. After centrifugation (2000 g, 10 min), it was washed three times with PBS to prepare a 4% red blood cell suspension. PBS treatment was used as a negative control, and 0.1% Triton X-100 was used as a positive control. The experimental group used PBS as a solvent. A 4% suspension of red blood cells was combined with an equal volume of the sample under examination and then incubated at a temperature of 37 ± 1 °C for 3 hours. Finally, the supernatant was collected by centrifuging at 2000 g for 10 minutes to determine the UV absorbance at 540 nm.

**Cell Scratching Assay**

Sterilized thin hydrogels were spread on the bottom of 6-well plates, and NIH-3T3 cells in a good growth state were inoculated on the surface of the hydrogels. Upon reaching 90% confluence, the surface of the hydrogel was disrupted with a 200 µL pipette tip, and PBS was utilized to clean the detached cells. After that, cells were incubated in a cell culture box for 24 hours. The cell morphology was photographed by a microscope.

**Cell oxygenation assay**

In vitro cell oxygenation was investigated via fluorescence microscopy based on a hypoxia-sensitive fluorescent probe Ru(dpp). RAW264.7 cells were initially inoculated into 24-well plates at a density of 1 × 10^5^ cells/mL. After 12 hours of incubation, the cells were incubated with Ru(dpp) (10 μg/mL in fresh DMEM medium) under hypoxic conditions (1% O_2_, 5% CO_2_ and 94% N_2_). Then, 100 μL DMEM medium containing nanomaterials was supplemented to replace the previous medium and inoculated for 4 hours. After that, the cell images were obtained using CLSM.

**Intracellular Antioxidant Assay**

2',7'-Dichlorodihydrofluorescein diacetate (DCFH-DA) was a ROS fluorescent probe to evaluate ROS levels within cells. NIH-3T3, RAW264.7, and HUVEC cells were populated into confocal culture dishes and cultivated for 24 hours. After discarding the growth medium, cells underwent a 5 hours exposure to a medium containing 100 mM H_2_O_2_ and were treated with different materials. Following the suggested dosage for DCFH-DA usage, the cellular structure was examined through CLSM. Besides, the efficacies of the synthesized nanomaterials and hydrogels in neutralizing intracellular ROS were evaluated through a flow cytometer. The details are as follows: NIH-3T3, RAW264.7, and HUVEC cells were populated in six-well plates and received analogous treatments as outlined in prior DCFH-DA fluorescence staining tests. After a 30 minutes DCFH-DA treatment, cells were rinsed three times with PBS, followed by digestion with 0.25% trypsin-EDTA. Subsequently, their fluorescence intensity was measured via a flow cytometer.


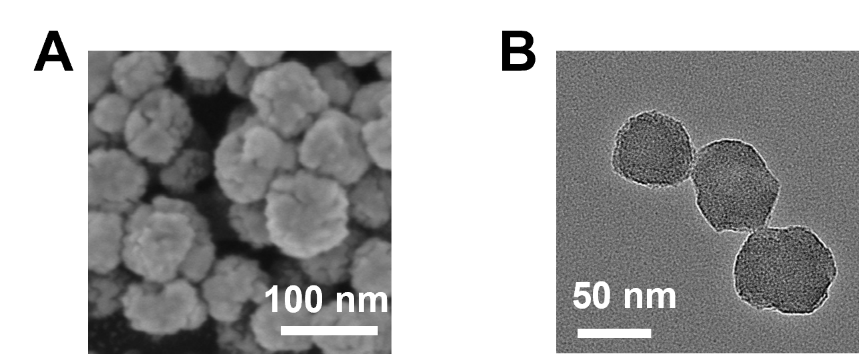


**Figure S1.** SEM (A) and TEM (B) images of PCN-224.


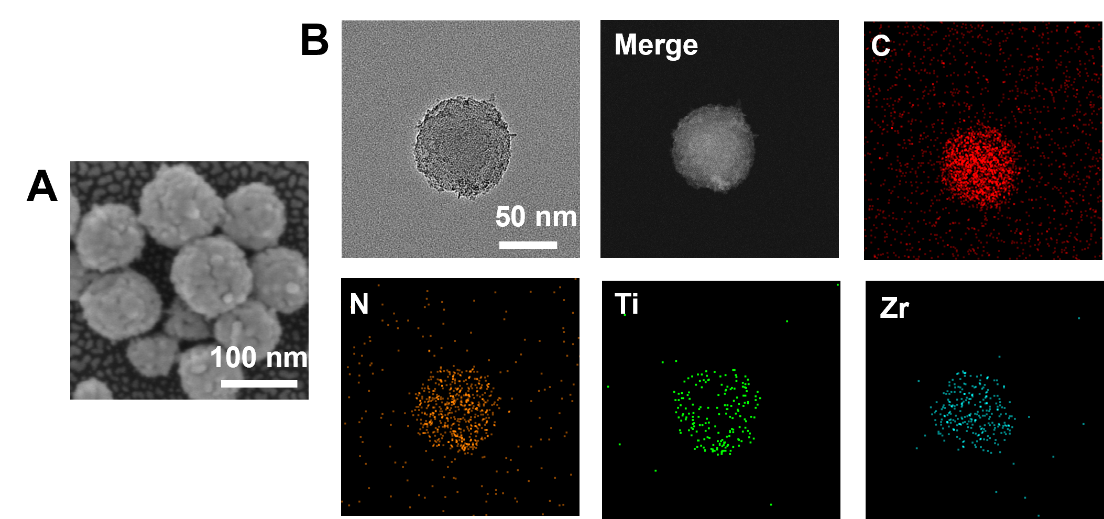


**Figure S2.** SEM (A), TEM and element mapping (B) images of PCN-224(Ti).


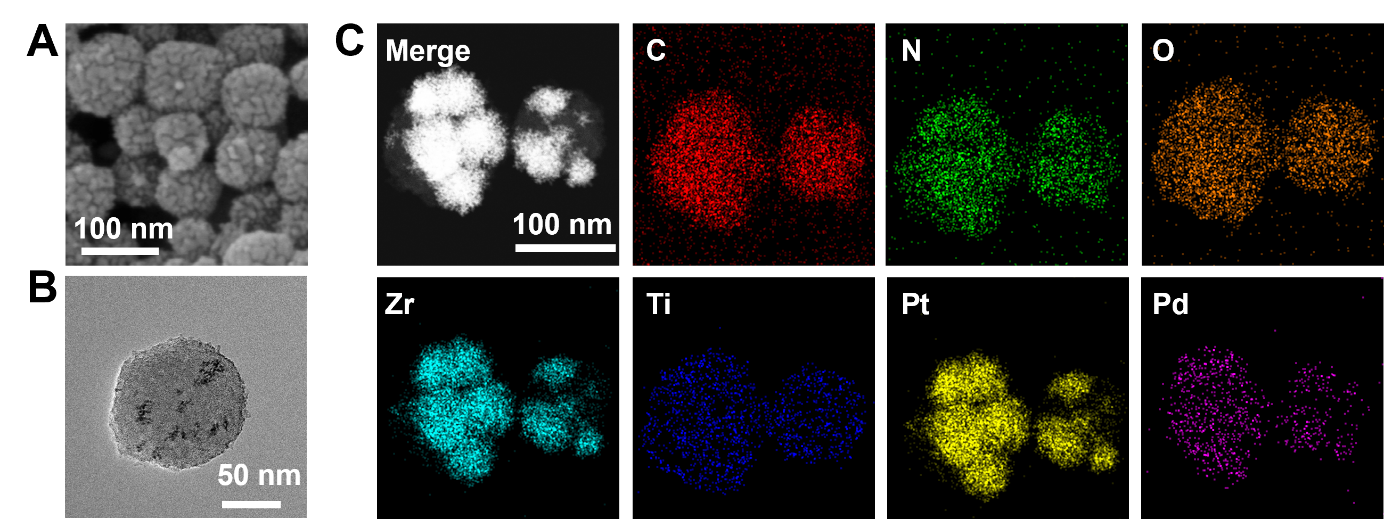


**Figure S3.** SEM (A), TEM (B) and element mapping (C) images of PTPP.


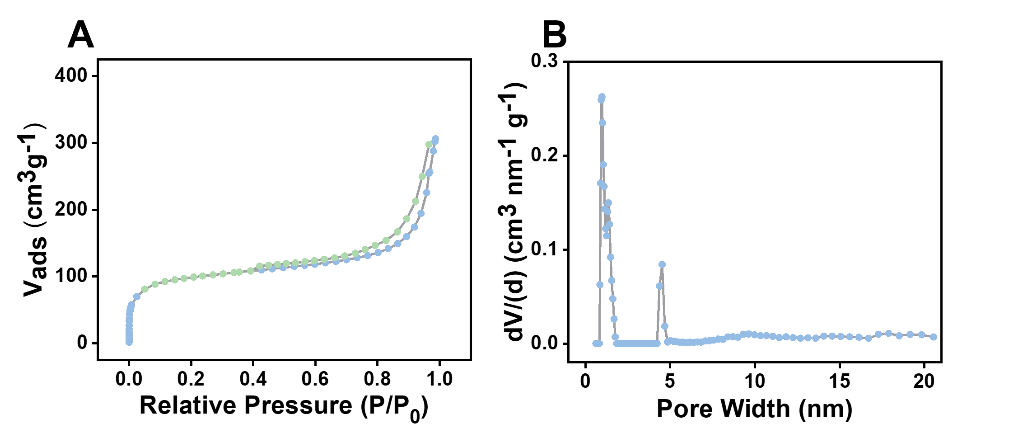


**Figure S4.** N_2_ adsorption-desorption isotherms (A) and the corresponding pore-size distribution of PTPPG (B).


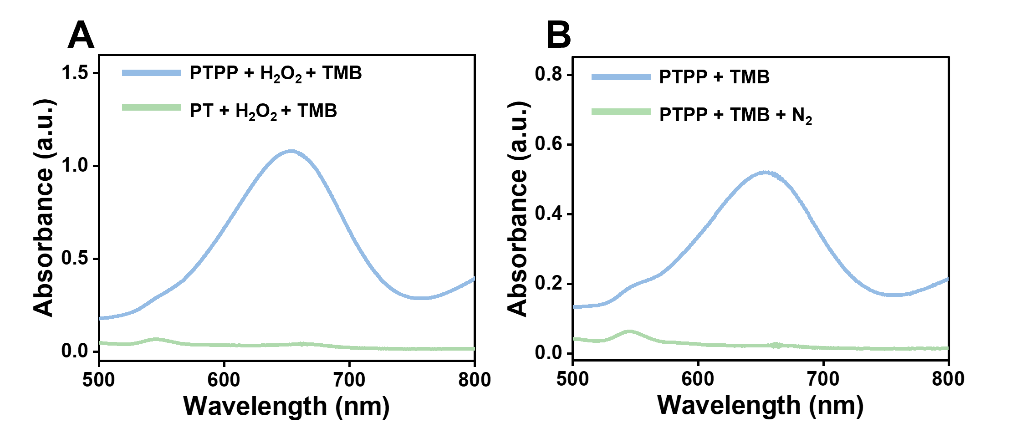


**Figure S5.** POD (A) and (B) OXD activities of 5 µg/mL PTPP verified by the addition of 500 μM TMB in the presence and absence of 5 mM H_2_O_2_ for 5 minutes, respectively.


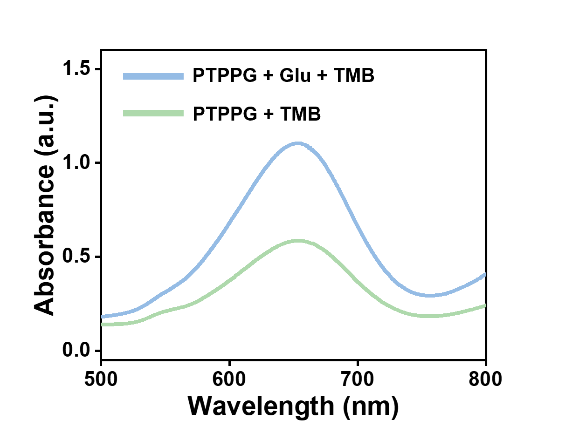


**Figure S6.** GOx and POD activity of PTPPG and the feasibility of the cascade reaction verified by TMB assay.


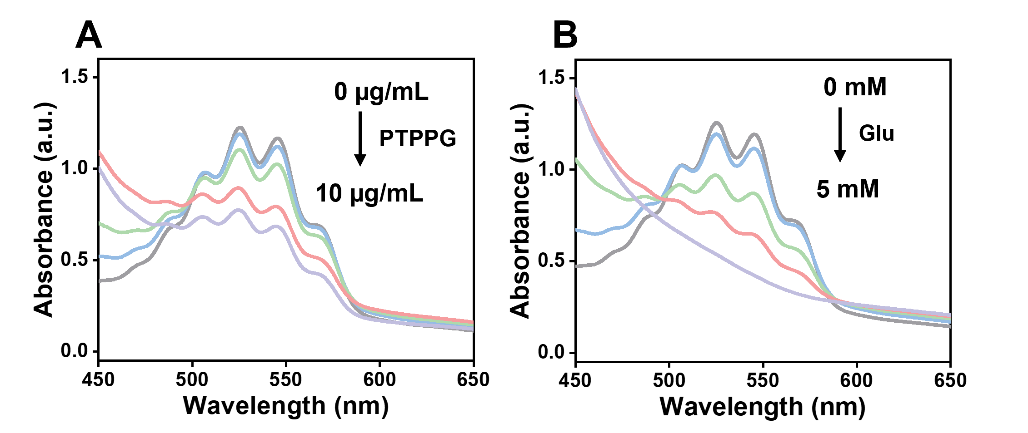


**Figure S7.** Demonstration of the generation of H_2_O_2_ in the cascade reaction using KMnO_4_ as the detection reagent (H_2_O_2_ can make KMnO_4_ solution fade.). (A) The dependence of H_2_O_2_ production on PTPPG concentrations. (B) The dependence of H_2_O_2_ production on Glu concentrations.


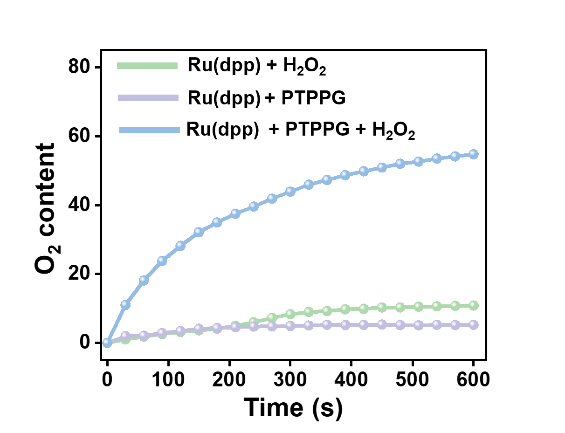


**Figure S8.** The dynamic observation of the decomposition of H_2_O_2_ into O_2_ by PTPPG.


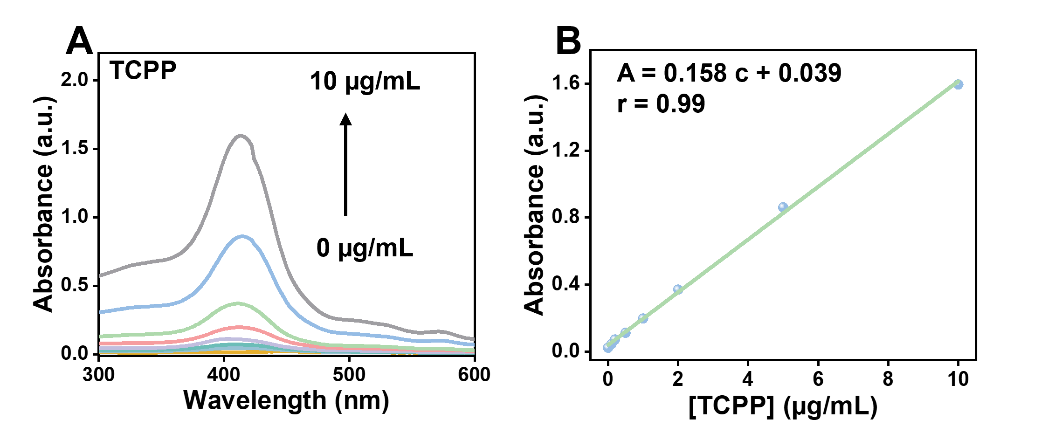


**Figure S9.** (A) UV-vis absorption spectra of different concentrations of TCPP and (B) the linear relationship between different concentrations of TCPP and the absorbance at 415 nm was obtained.


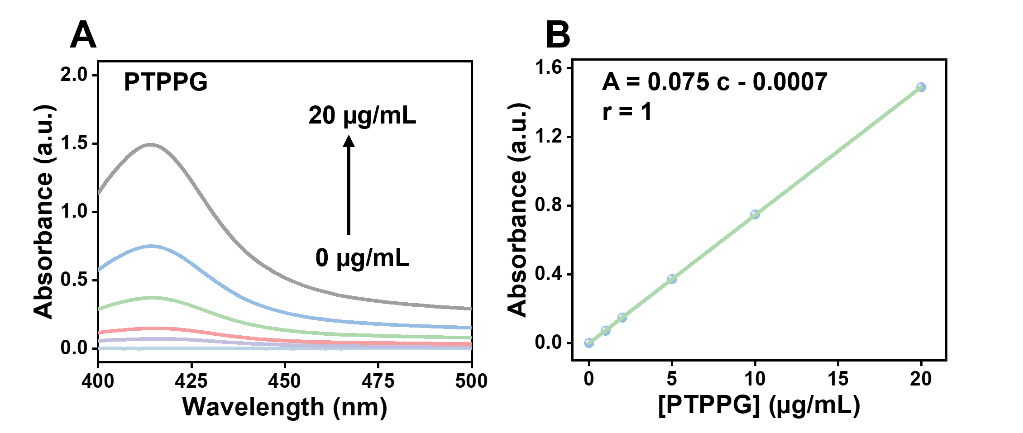


**Figure S10.** (A) UV-vis absorption spectra of different concentrations of PTPPG and (B) the linear relationship between different concentrations of PTPPG and the absorbance at 415 nm was obtained.


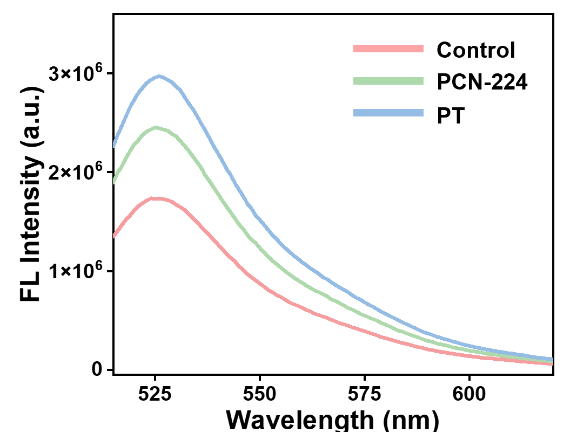


**Figure S11.** The photodynamic effects of TCPP (Control), PT and PCN-224 demonstrated using SOSG as a singlet oxygen fluorescent probe (5 µM).


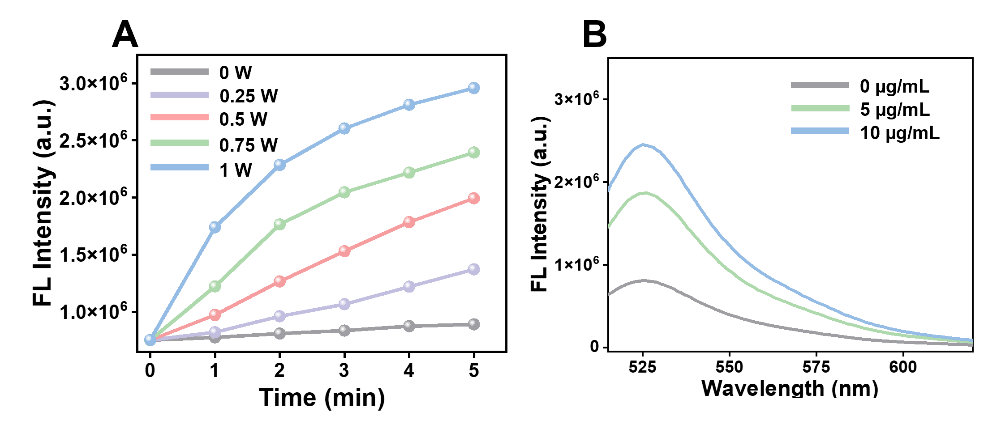


**Figure S12.** Photodynamic effect of PTPPG at varying power densities for various times (A) and PTPPG concentrations (B) verified using SOSG as a singlet oxygen fluorescent probe (5 µM).


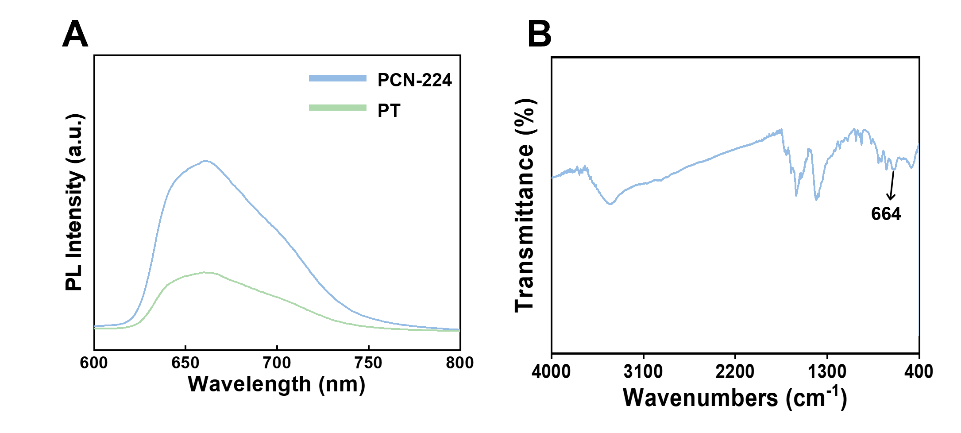


**Figure S13.** Photoluminescence of PCN-224 and PT nanoparticles (5 µg/mL) (A) and FT-IR spectrum of PT nanoparticles (B).


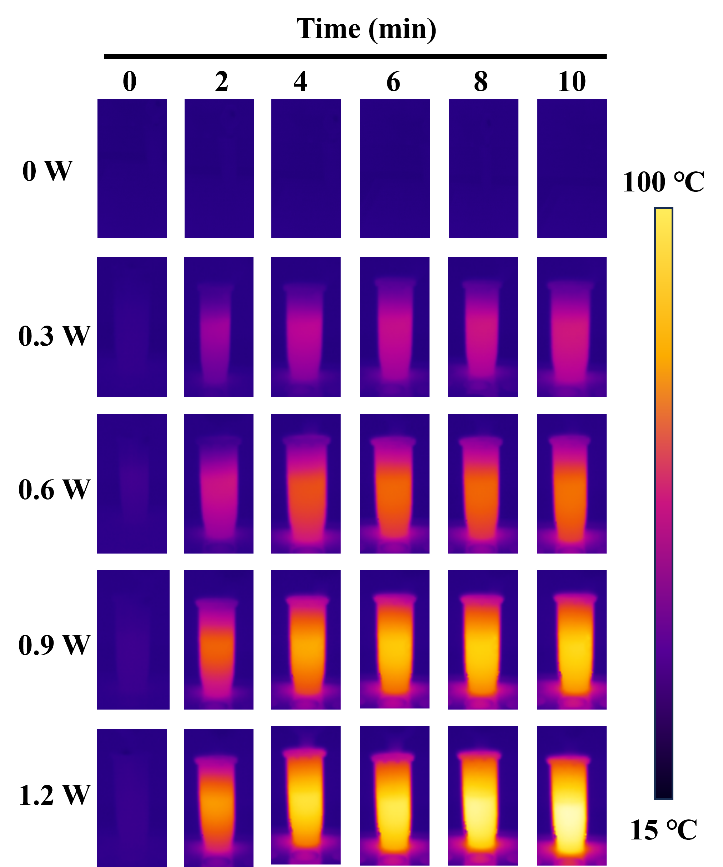


**Figure S14.** Photothermal images of PTPPG (100 µg/mL) irradiated by 808 nm laser at different power densities.


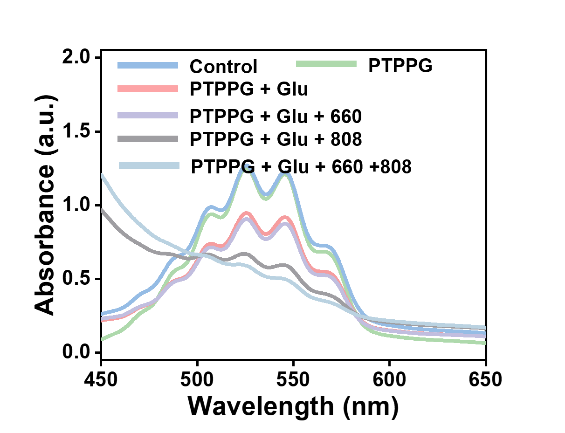


**Figure S15.** The effect of 660 nm and 808 nm laser irradiation on enzymatic activities of GOx loaded on PTPPG tested by KMnO_4_.


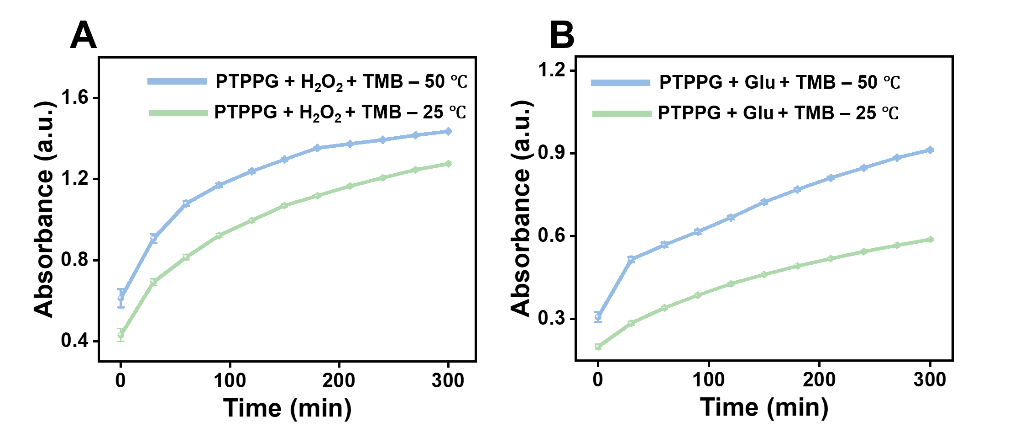


**Figure S16.** The effect of photothermal temperature on PTPPG enzymatic activity verified by a solution containing 500 µM TMB at pH of 4 in the presence of 5 mM H_2_O_2_ (A) and Glu (20 mM) (B) as the substrate, respectively.


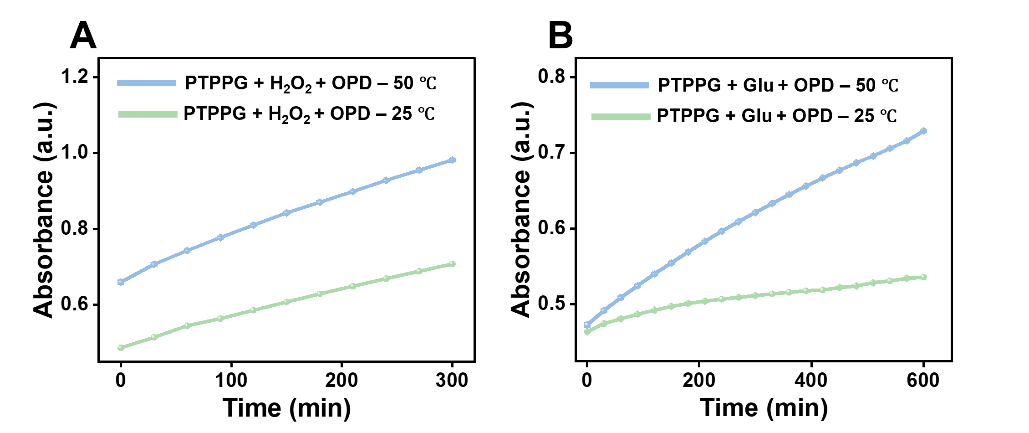


**Figure S17.** The effect of photothermal temperature on PTPPG enzymatic activity verified by a solution containing 500 µM OPD at pH of 7.4 in the presence of 5 mM H_2_O_2_ (A) and Glu (20 mM) (B) as the substrate, respectively.


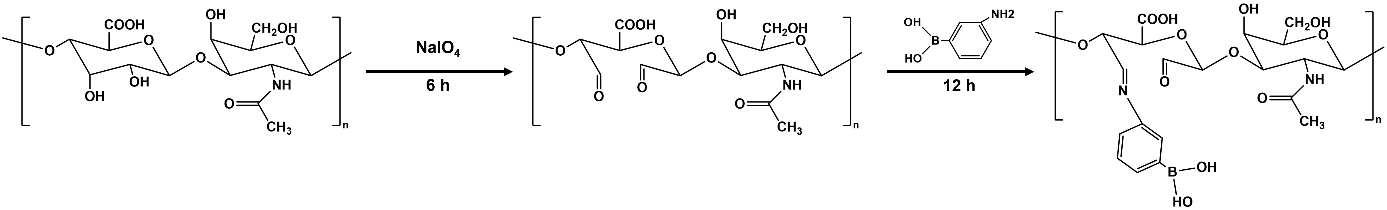


**Figure S18.** The synthetic route of OHA-PBA.


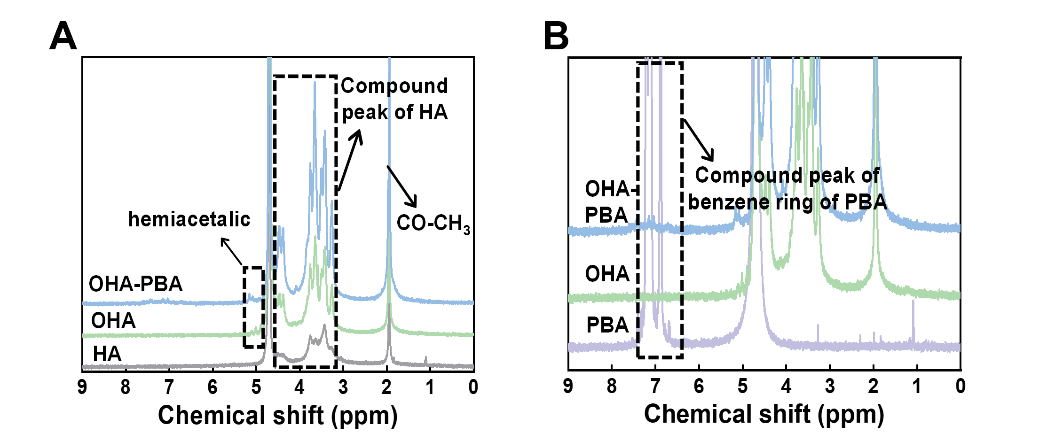


**Figure S19.** (A) ^1^H NMR spectra of characteristic and basic bonds of HA, OHA, and OHA-PBA, respectively. (B) ^1^H NMR spectra of characteristic and basic bonds of PBA, OHA and OHA-PBA, respectively.


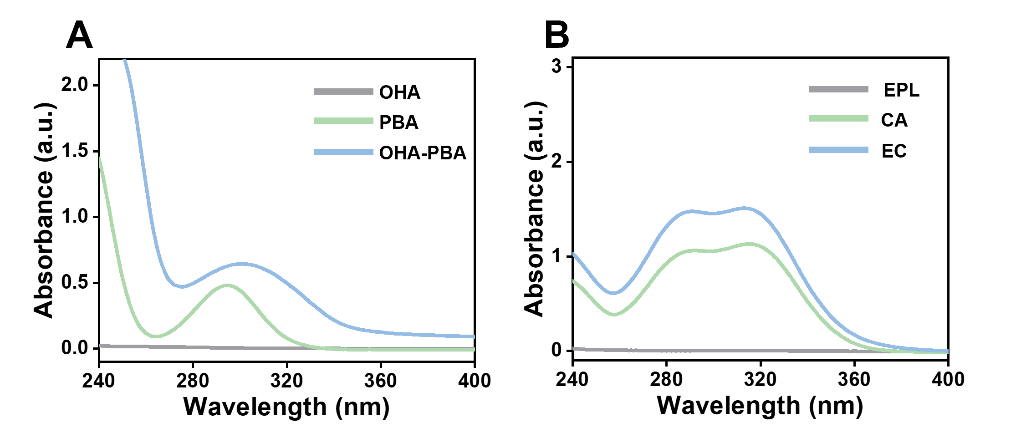


**Figure S20.** The successful synthesis of OHA-PBA (A) and EC (B) verified by UV-vis spectroscopy.

**Figure S21.** The synthetic route for EC.


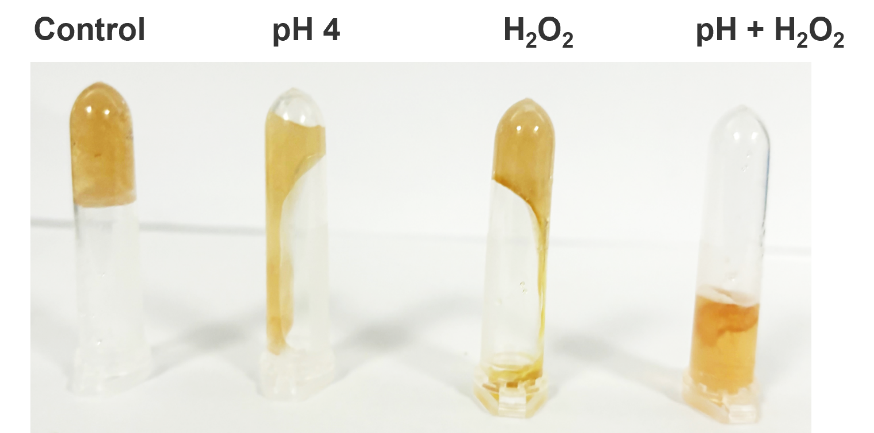


**Figure S22.**  The responsiveness of HE gels to pH and reactive oxygen species was evaluated in various conditions: a acetate buffered solution at pH 7.4, a acetate solution at pH 4 with or without the addition of 100mM H_2_O_2_, and a acetate solution containing 100mM H_2_O_2_ at pH 4.


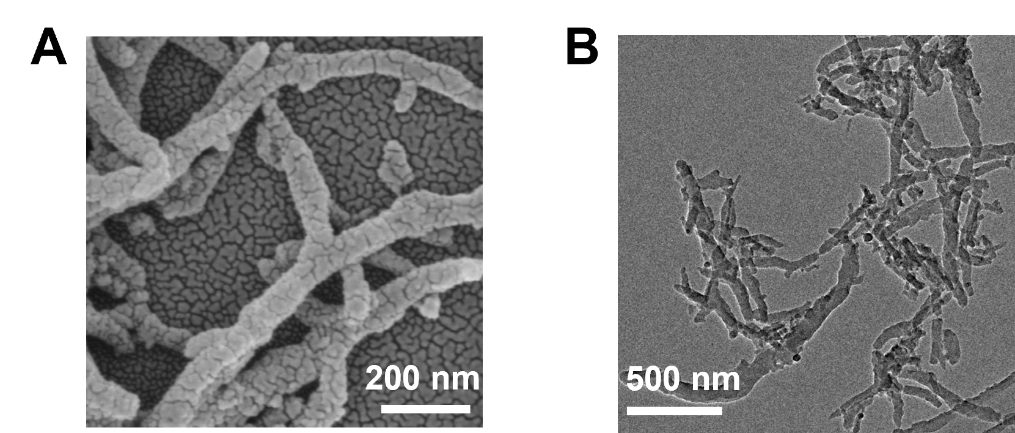


**Figure S23.**  SEM (A) and TEM images (B) of PDA@PPY NWs.


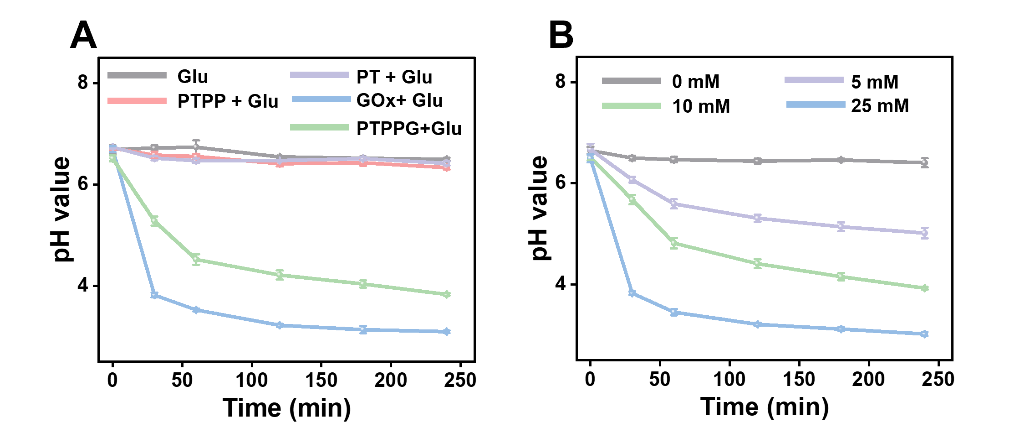


**Figure S24.** (A) The verification of pH change induced by Glu decomposition in the presence of PTPPG. (B) The change of pH in solutions containing different concentrations of Glu decomposed by PTPPG.


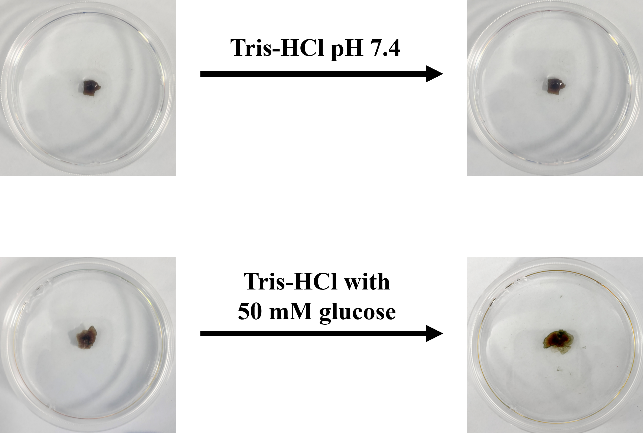


**Figure S25.** The release of PTPPG nanoparticles was verified in Tris-HCl solution with or without 50 mM glucose.


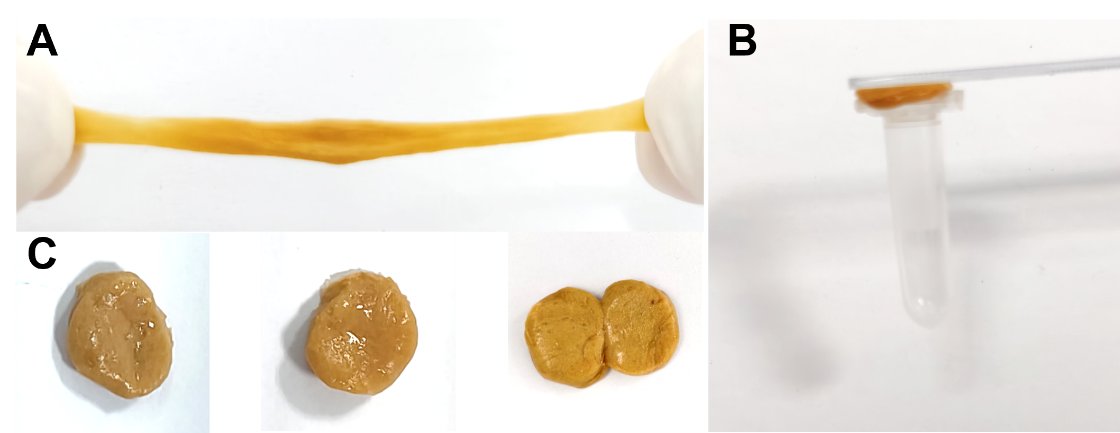


**Figure S26.** The stretchability (A), adhesion ability (B) and resilience (C) of HE.


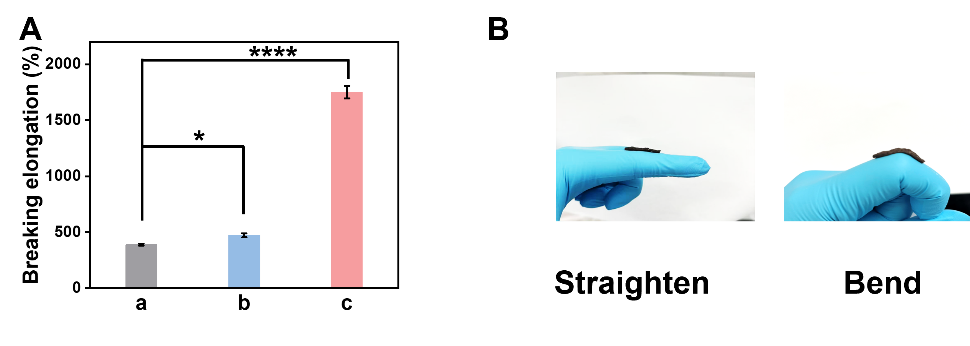


**Figure S27.** (A)The elongation at the break of different hydrogels with various components obtained via an electronic universal testing machine. (B) The tensile performance of the HEPP hydrogel at the joint. Error bars represent the mean ± standard deviation for a sample size of 3. *p < 0.05, **p < 0.01, ***p < 0.001, ****p < 0.0001. (a~c columns in section A represent HE hydrogel, HE hydrogel doped with PTPPG, HEPP gel, respectively).


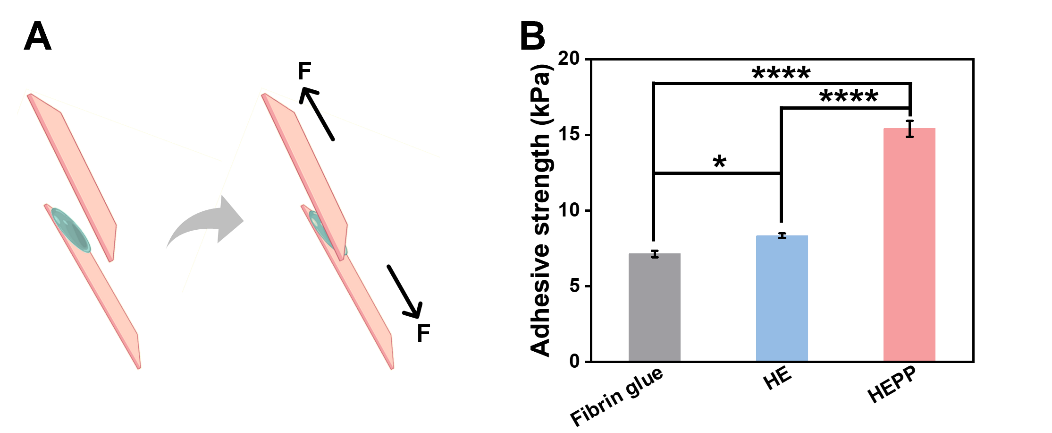


**Figure S28.** Evaluation of the adhesive capacity of hydrogels to the skin simulated by porcine skin. (A) Schematic of the adhesion property of hydrogels to the surface of skins. (B) The adhesive strength of the hydrogels to porcine skin using fibrin glue, HE and HEPP, respectively. Error bars represent the mean ± standard deviation for a sample size of 3. *p < 0.05, **p < 0.01, ***p < 0.001, ****p < 0.0001.


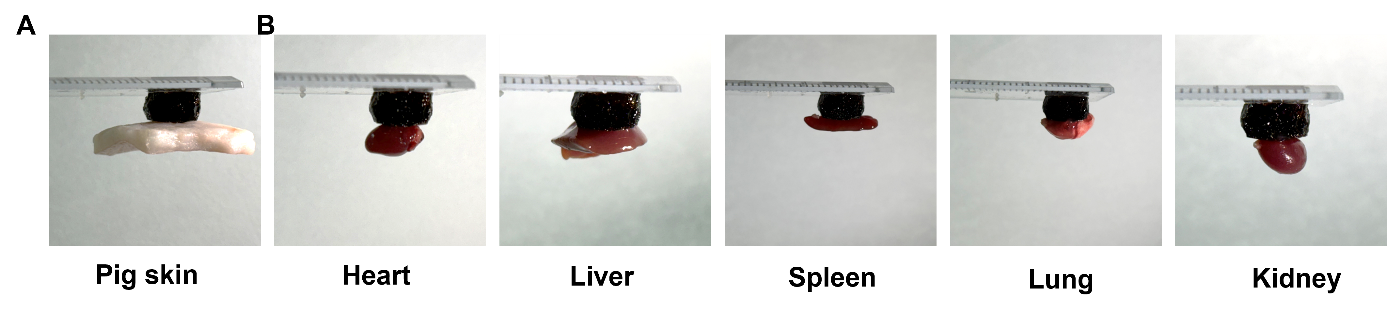
**Figure S29.** Adhesive efficacy of the HEPP dressing on pig skin (A) and various mouse organs (B).


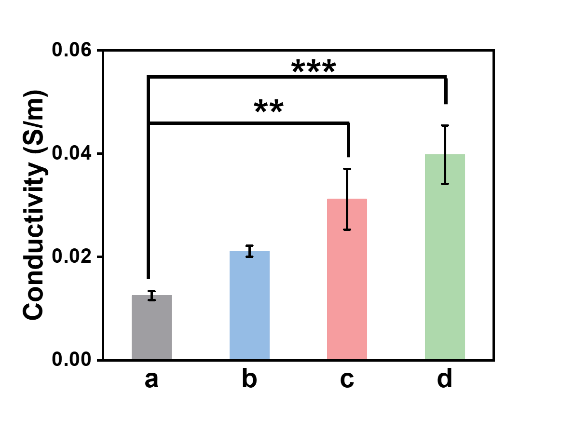


**Figure S30.** The conductivity of the HEPP doped with different levels of PDA@PPY. (a~d represent the conductivities of hydrogels with PDA@PPY doping levels of 0%, 5%, 10%, 20%, respectively). Error bars represent the mean ± standard deviation for a sample size of 3. *p < 0.05, **p < 0.01, ***p < 0.001, ****p < 0.0001.


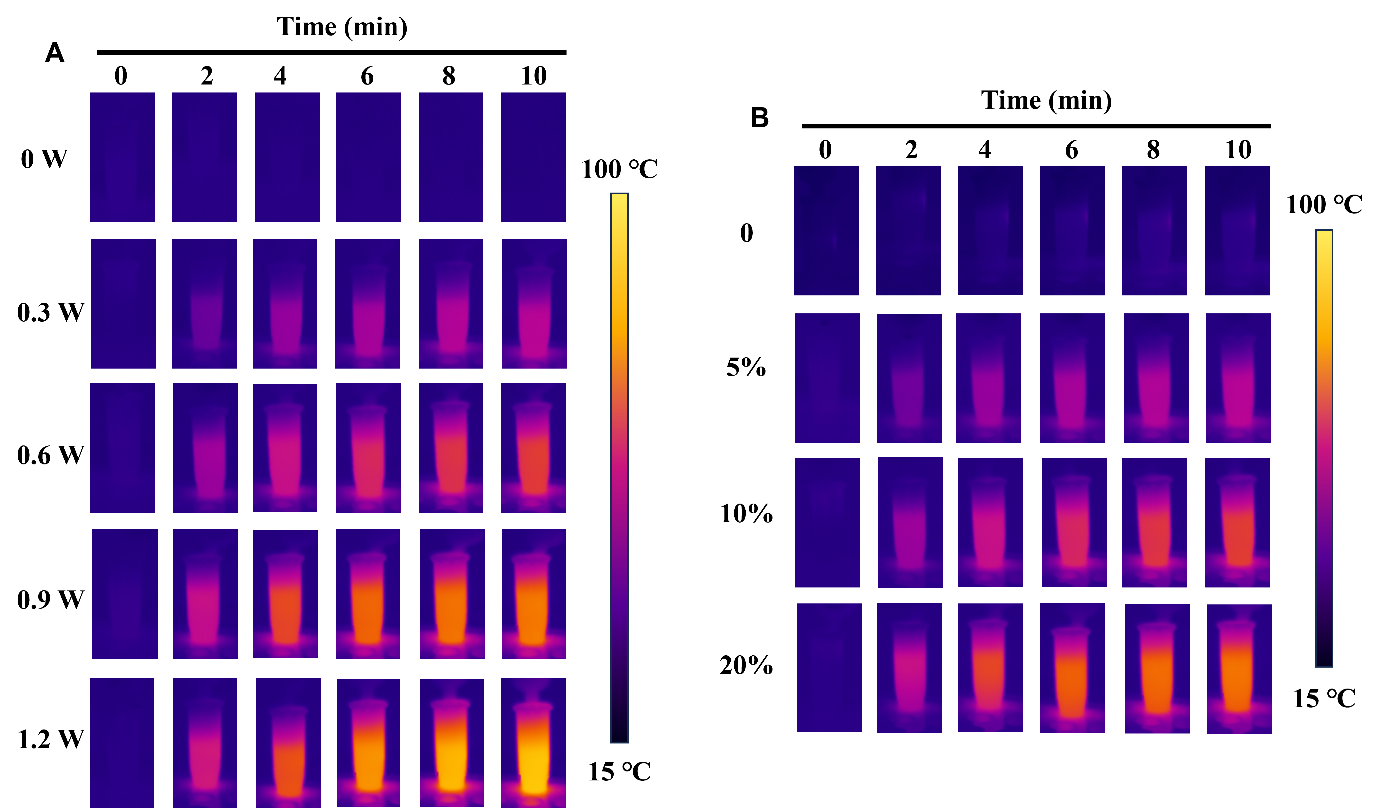


**Figure S31.** (A) Thermographic images of HEP (500 µL) under different power densities. (B) Thermographic images of HEP with different PDA@PPY levels.


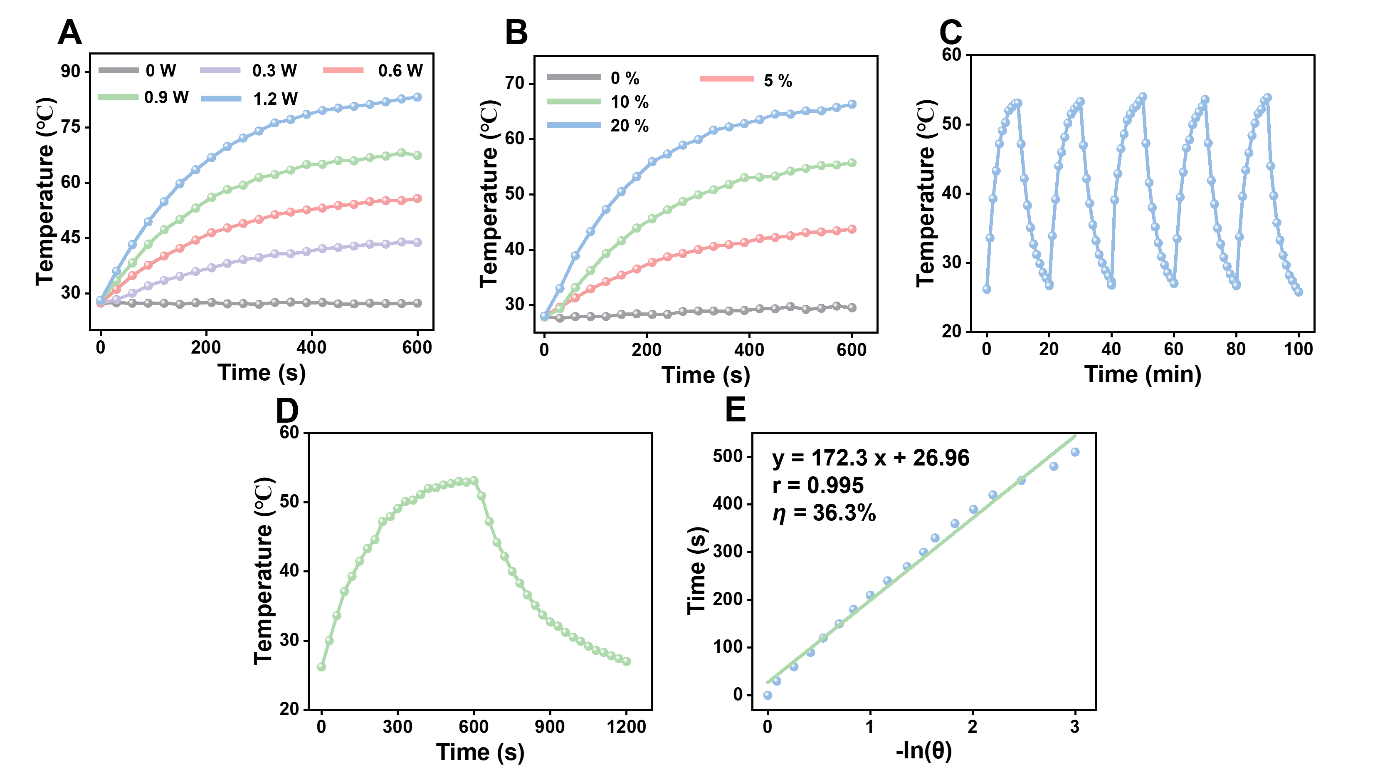


**Figure S32.** Curves depicting temperature increase of HEP (500 µL) at varying power intensities (A) and doping levels (B) under 808 nm NIR exposure. (C) The photostability evaluation of 10% HEP exposed to a laser at 808 nm (0.9 W). (D) Curves indicating the temperature variations of 10% HEP during and after the process of laser exposure (808 nm, 0.9 W). (E) Linear approximation between −ln(θ) and time for HEP.


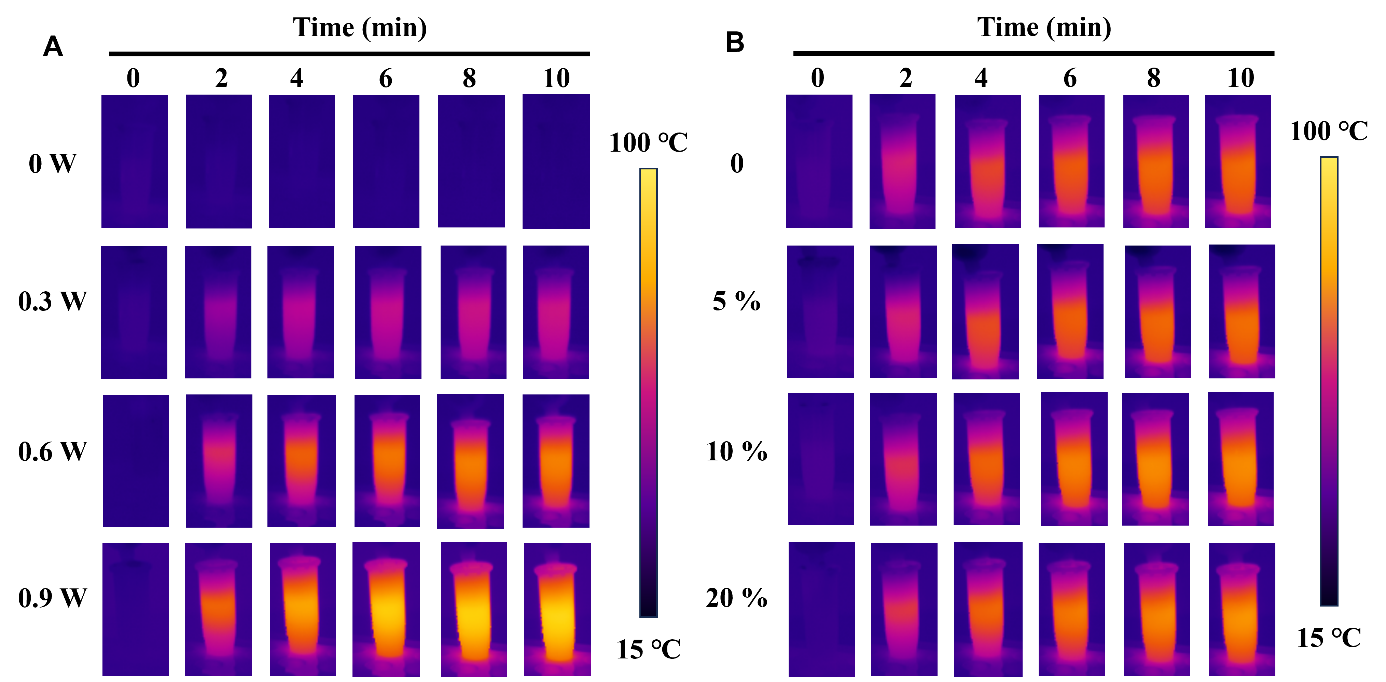


**Figure S33.** (A) Thermographic images of HEPP (500 µL) under different power intensities (A) and PDA@PPY loading levels (B).


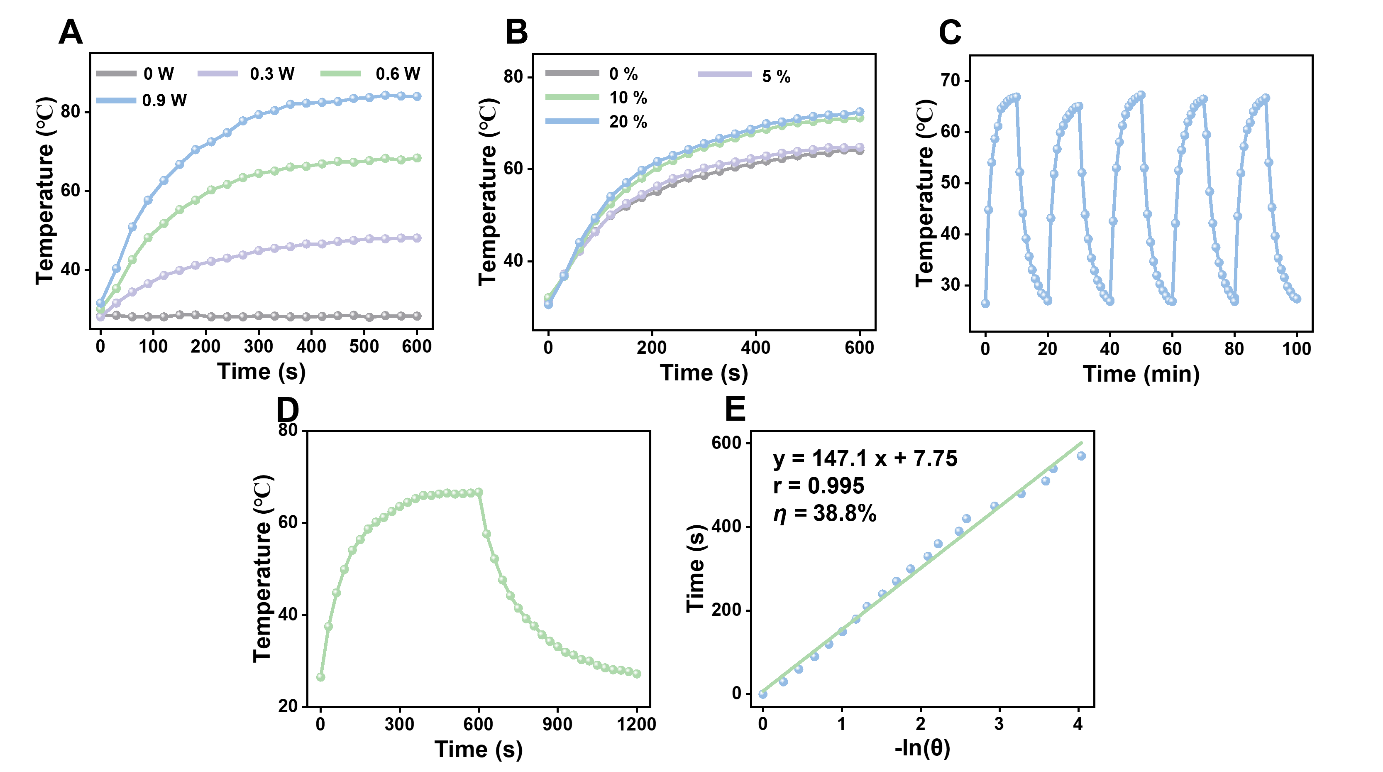


**Figure S34.** Curves depicting temperature increase of HEPP (500 µL) under 808 nm NIR exposure at varying power intensities (A) and PTPPG levels (B). (C) The photostability evaluation of HEPP exposed to a laser at 808 nm (0.9 W). (D) Temperature variations of 10% HEPP during and after laser exposure (808 nm, 0.9 W). (E) Linear approximation between −ln(θ) and time for HEPP.


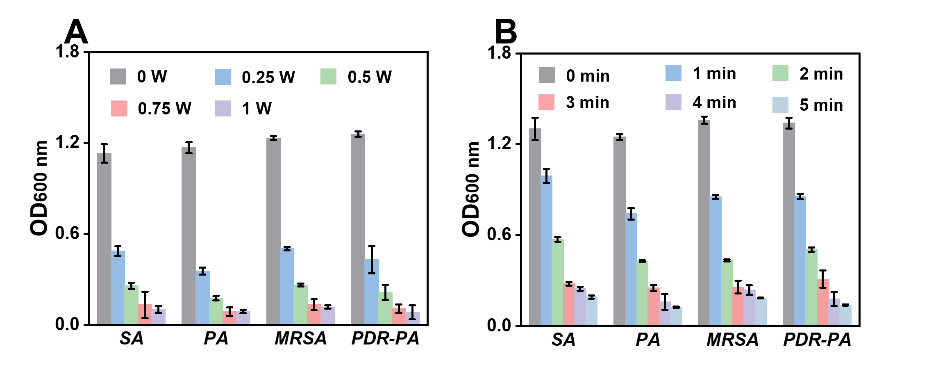


**Figure S35.** The OD 600 nm values after 12 h incubation of Gram-negative bacteria and Gram-positive bacteria treated with HEPP under 660 nm laser irradiation at different power intensities (A), and for different irradiation times at 660 nm (1 W) (B).


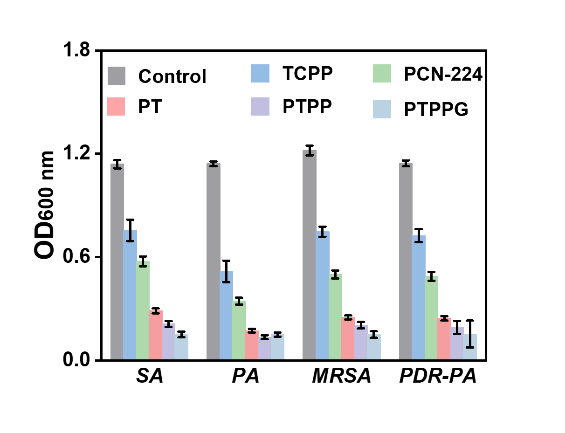


**Figure S36.** The OD 600 nm values after 12 h incubation of Gram-negative bacteria and Gram-positive bacteria treated by different materials with laser (660nm, 1 W).


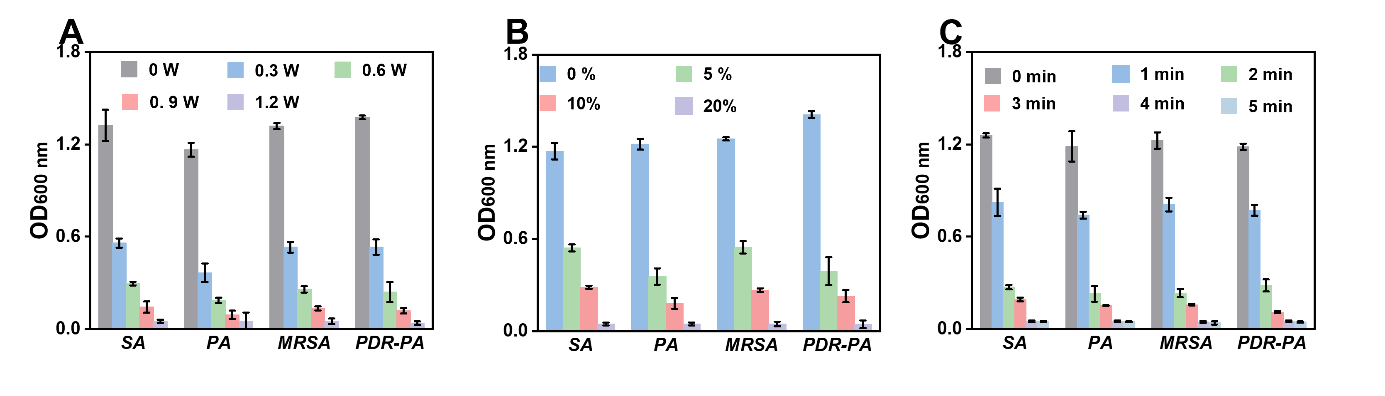


**Figure S37.** The OD 600 nm values after 12 h incubation of Gram-negative bacteria and Gram-positive bacteria treated with HEPP under different power intensities of 808 nm laser (A), PDA@PPY loading rates (B), and irradiation times at 808 nm (C).


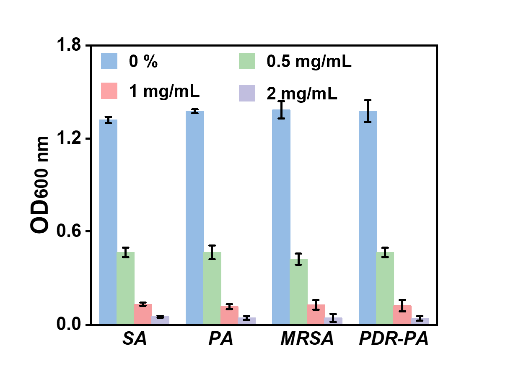


**Figure S38.** The OD 600 nm values after 12 h incubation of Gram-negative bacteria and Gram-positive bacteria treated by HEPP with different PTPPG loading rates.


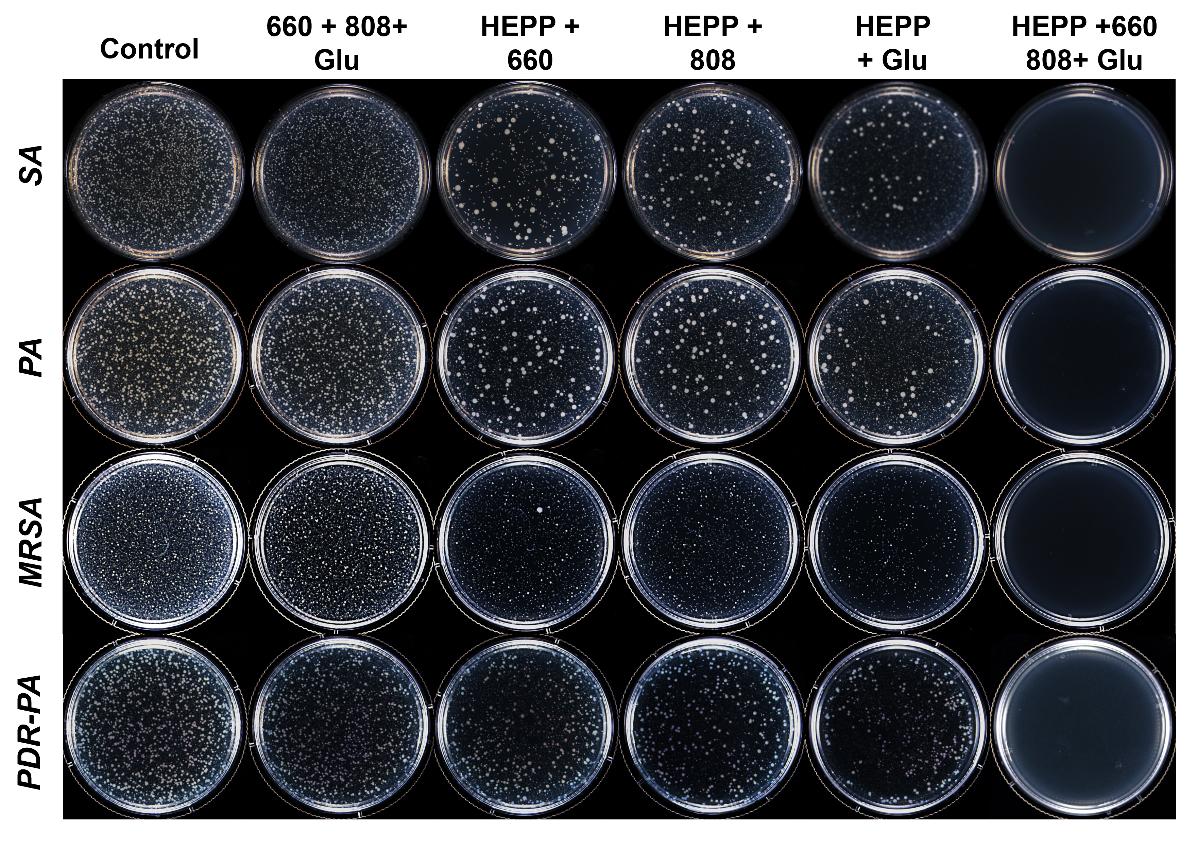


**Figure S39.** Images of colonies on agar plates after being treated with PDT, CDT and PTT separately or synergistically.


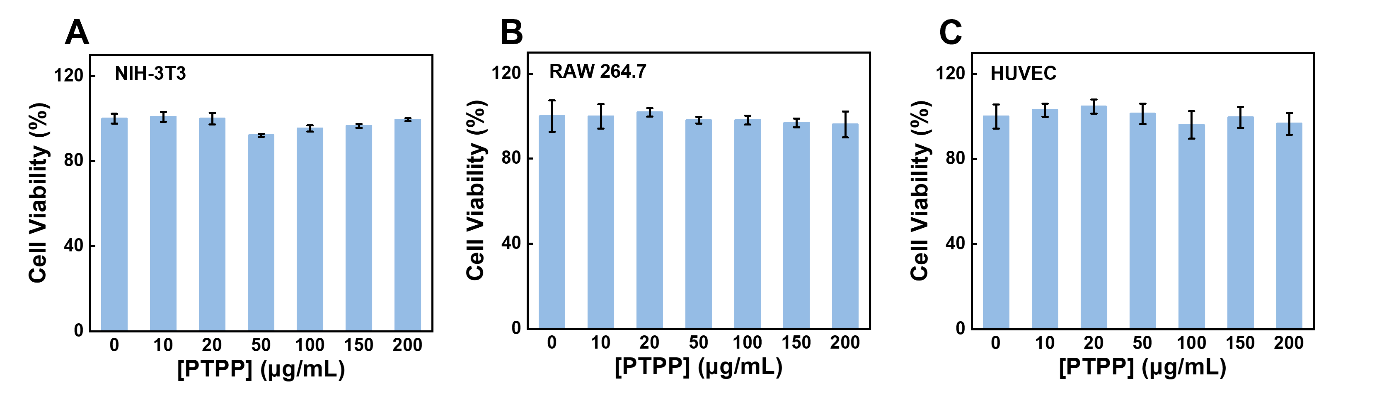


**Figure S40.** NIH-3T3 cells (A), RAW264.7 cells (B) and HUVEC cells (C) toxicities of PTPPG evaluated by CCK-8 kits.


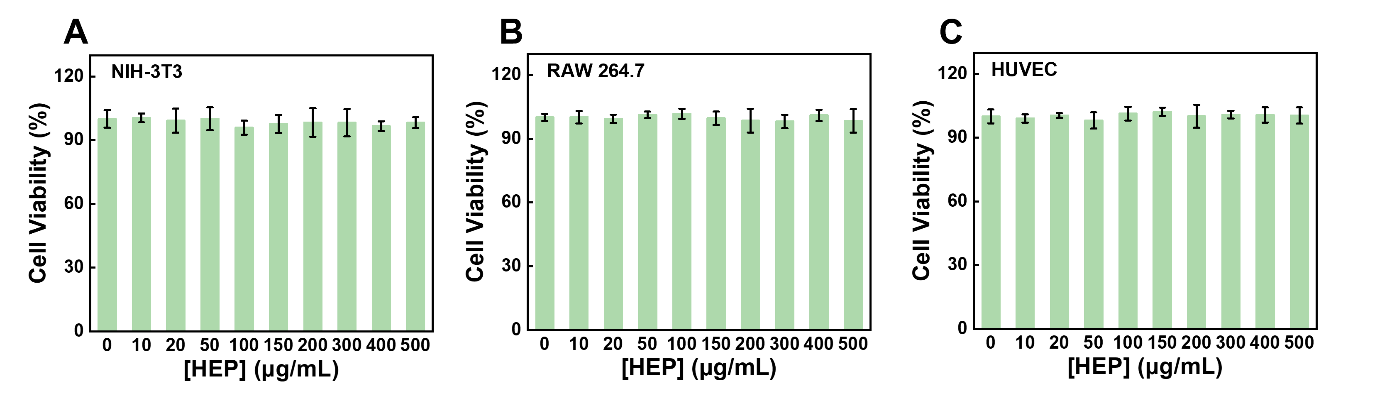


**Figure S41.** NIH-3T3 cells (A), RAW264.7 cells (B) and HUVEC cells (C) toxicities of HEP evaluated by CCK8 kits.


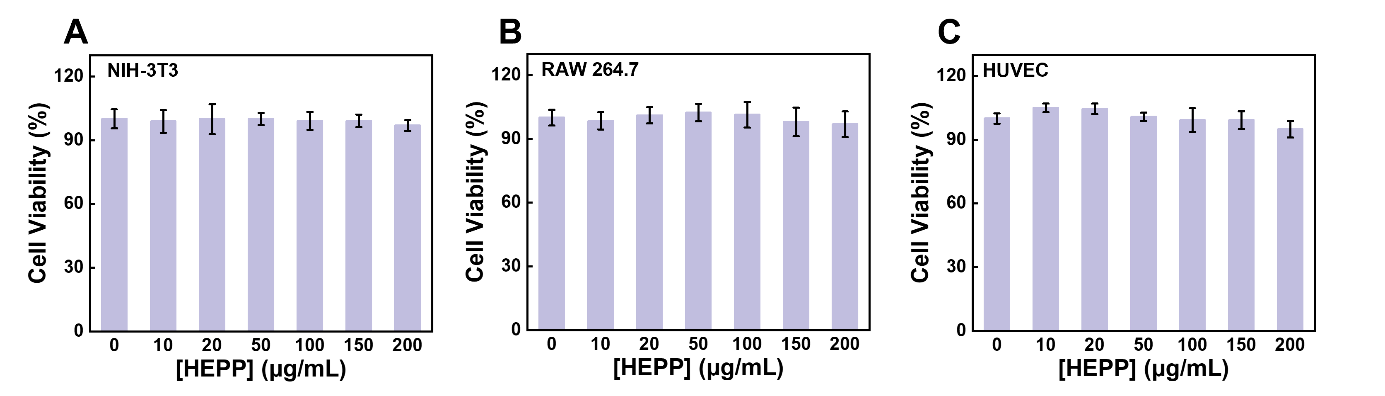


**Figure S42.** NIH-3T3 cells (A), RAW264.7 cells (B) and HUVEC cells (C) toxicities of HEPP evaluated by CCK8 kits.


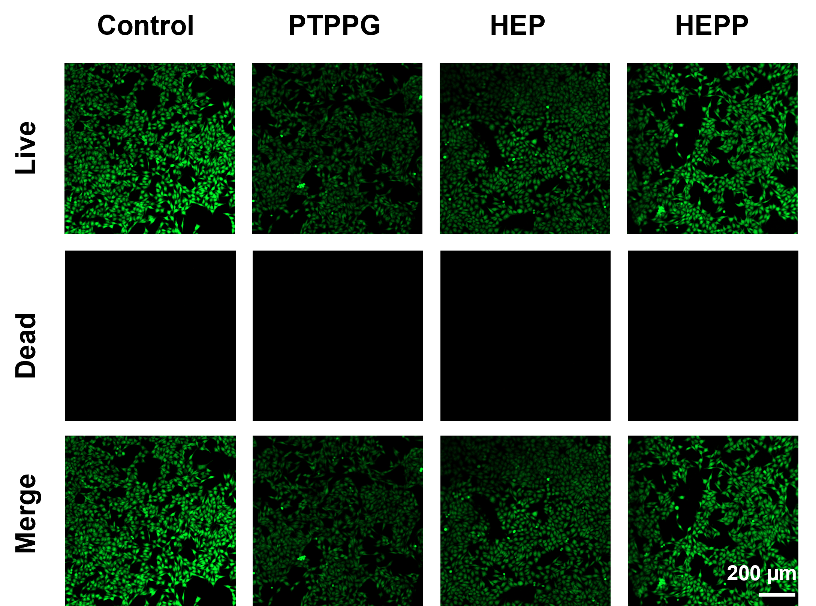


**Figure S43.** Live-dead fluorescence staining of NIH-3T3 cells treated with different materials.


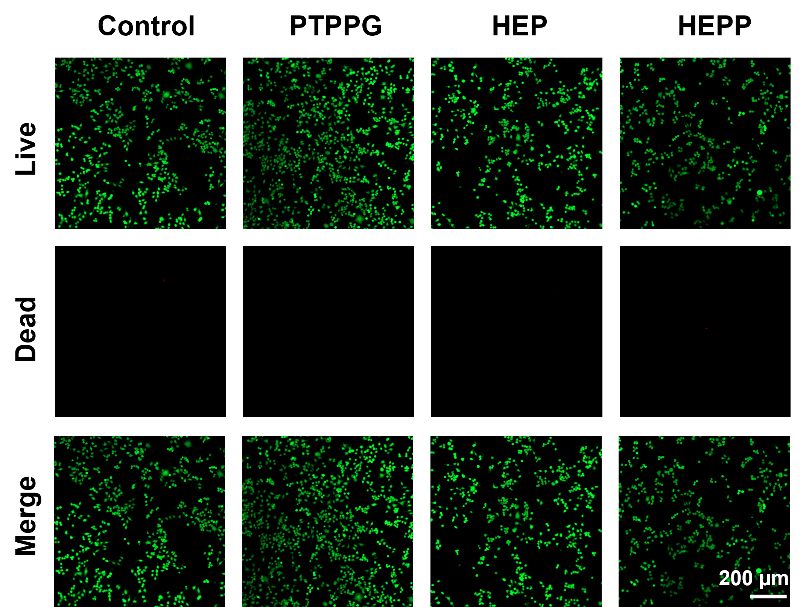


**Figure S44.** Live-dead fluorescence staining of RAW264.7 cells treated with different materials.


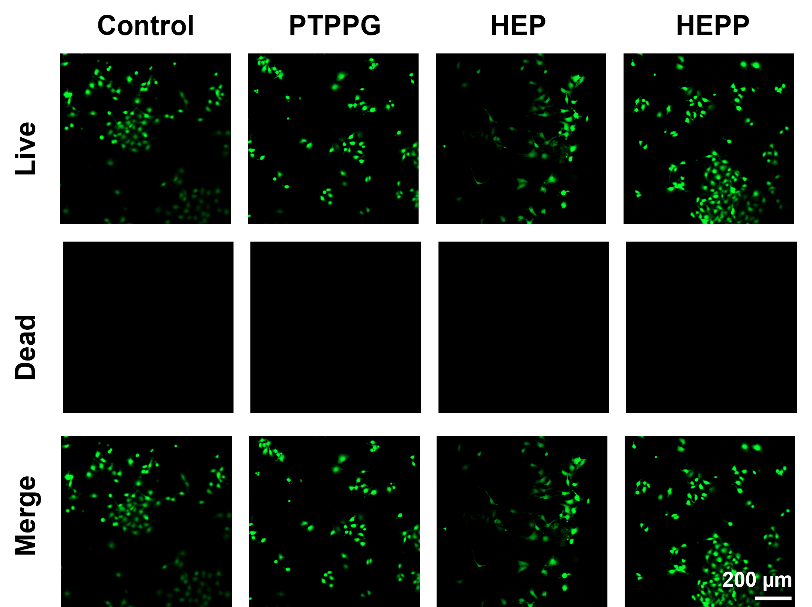


**Figure S45.** Live-dead fluorescence staining of HUVEC cells treated with different materials.


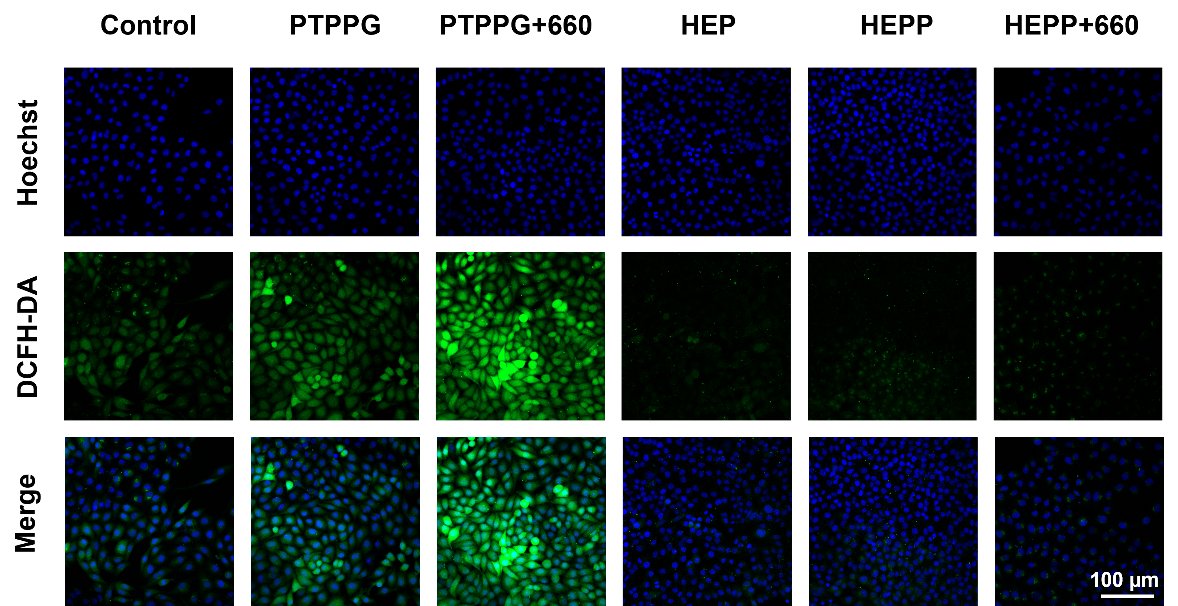


**Figure S46.** ROS fluorescence staining of NIH-3T3 cells treated with different materials.


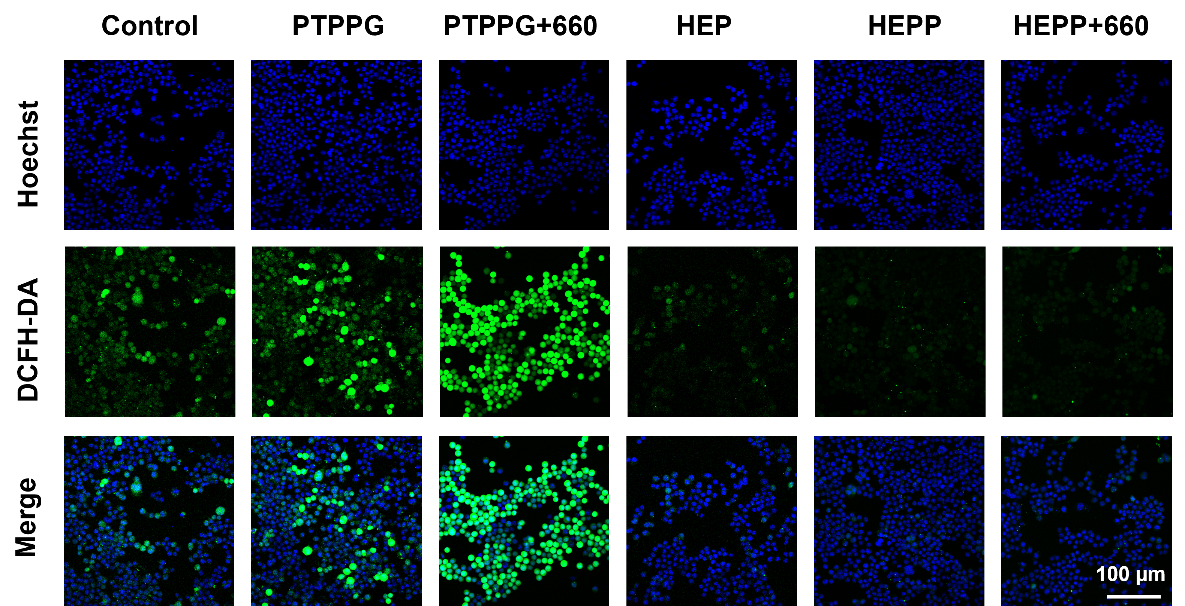


**Figure S47.** ROS fluorescence staining of RAW 264.7 cells treated with different materials.


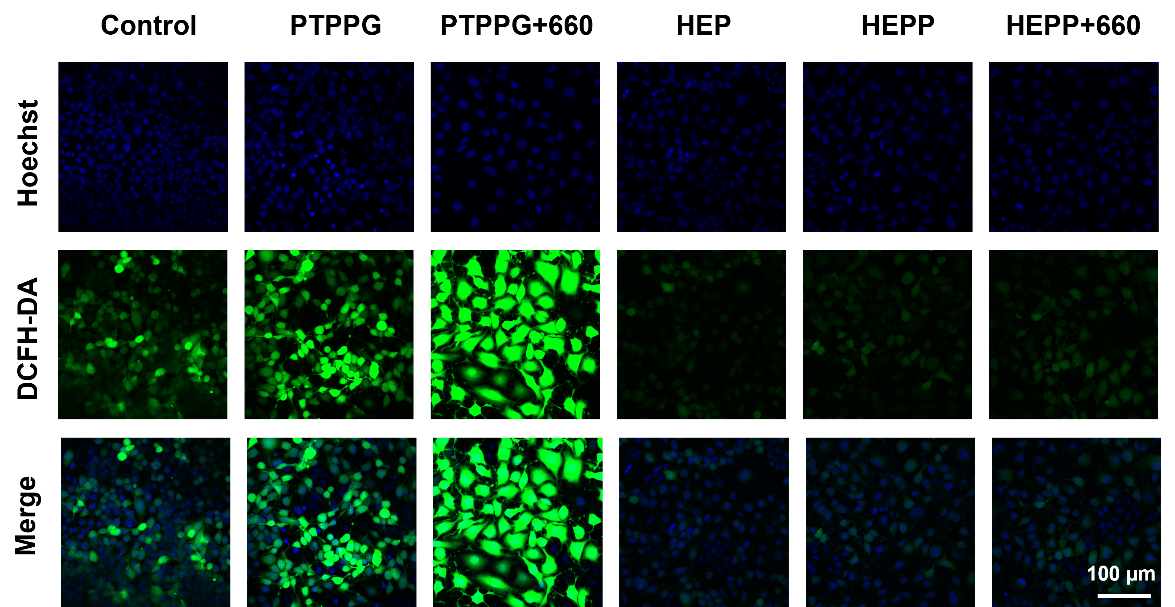


**Figure S48.** ROS fluorescence staining of HUVEC cells treated with different materials.


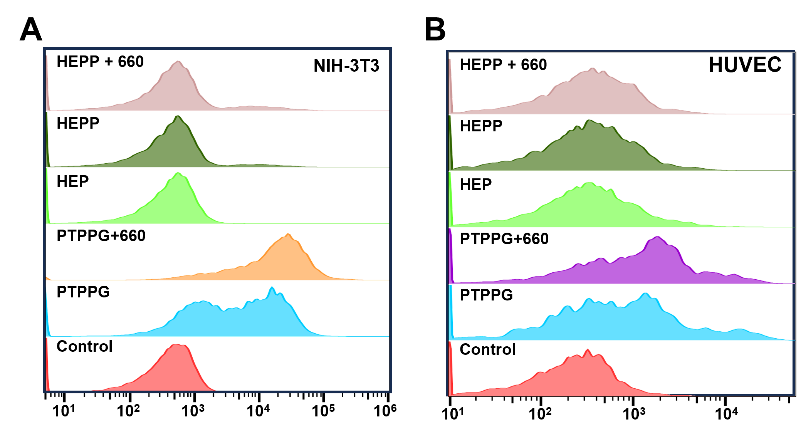


**Figure S49.** Flow cytometry results of DCFH-DA fluorescence in NIH-3T3 (A) and HUVEC cells (B) after different treatments.


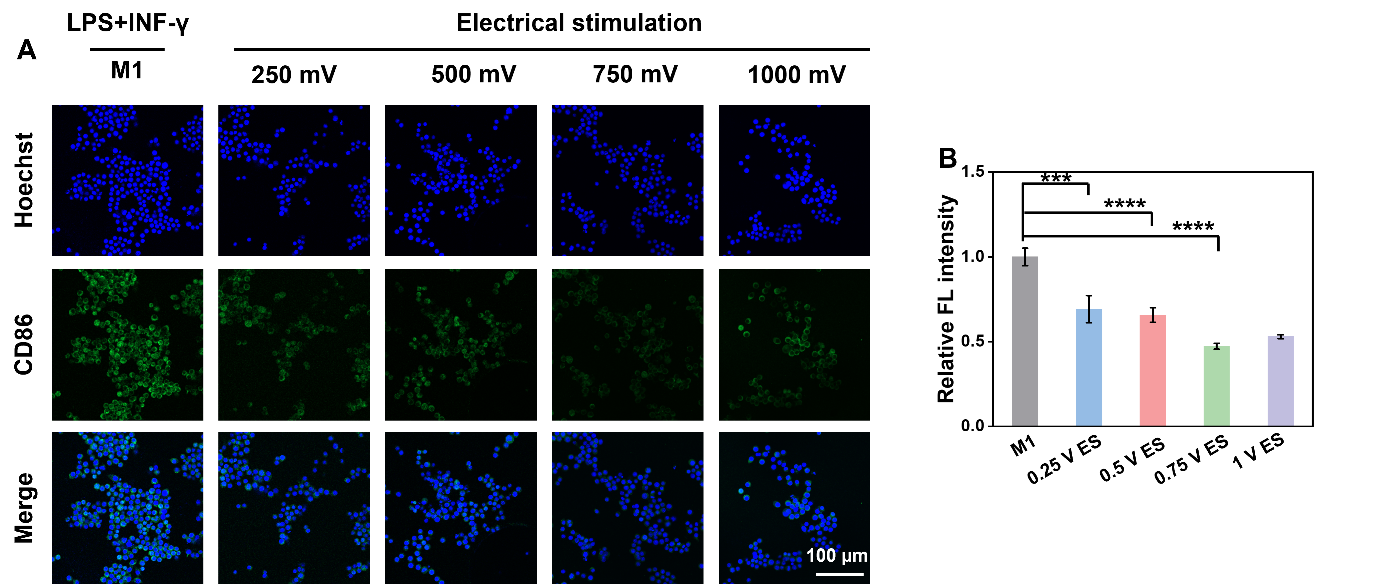


**Figure S50.** (A) Immunofluorescence staining of CD86 expression in RAW264.7 cells under electrical stimulation at different voltages. (B) Assessment of fluorescence relative intensities of CD86 in RAW264.7 cells. Error bars represent the mean ± standard deviation for a sample size of 3. *p < 0.05, **p < 0.01, ***p < 0.001, ****p < 0.0001.


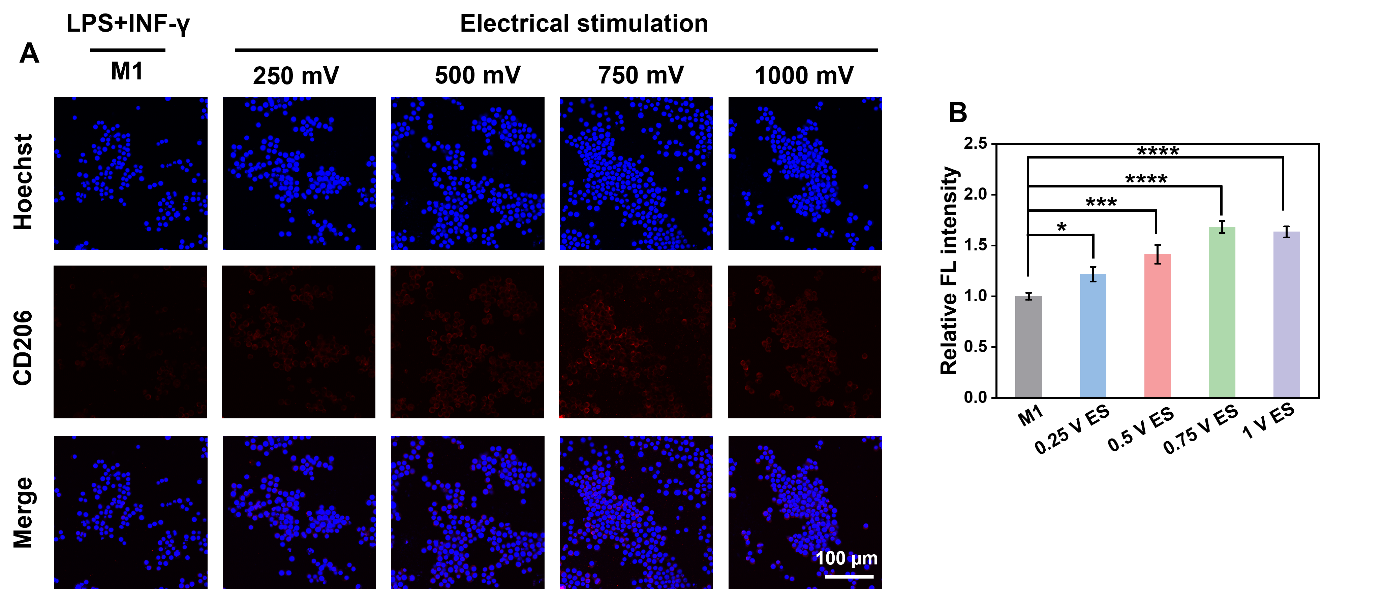


**Figure S51.** (A) Immunofluorescence staining of CD206 expression in RAW264.7 cells under electrical stimulation at different voltages. (B) Assessment of fluorescence relative intensities of CD206 in RAW264.7 cells. Error bars represent the mean ± standard deviation for a sample size of 3. *p < 0.05, **p < 0.01, ***p < 0.001, ****p < 0.0001.


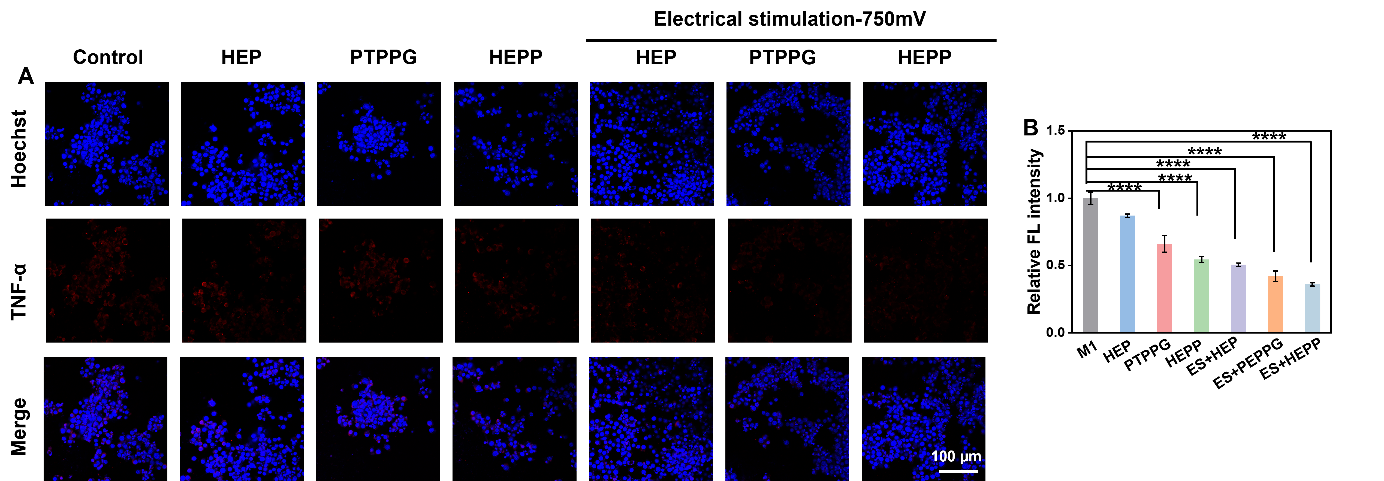


**Figure S52.** (A) Immunofluorescence staining of TNF-α expression in RAW264.7 cells under different stimulations. (B) Assessment of fluorescence relative intensities of TNF-α stains in RAW264.7 cells. Error bars represent the mean ± standard deviation for a sample size of 3. *p < 0.05, **p < 0.01, ***p < 0.001, ****p < 0.0001.


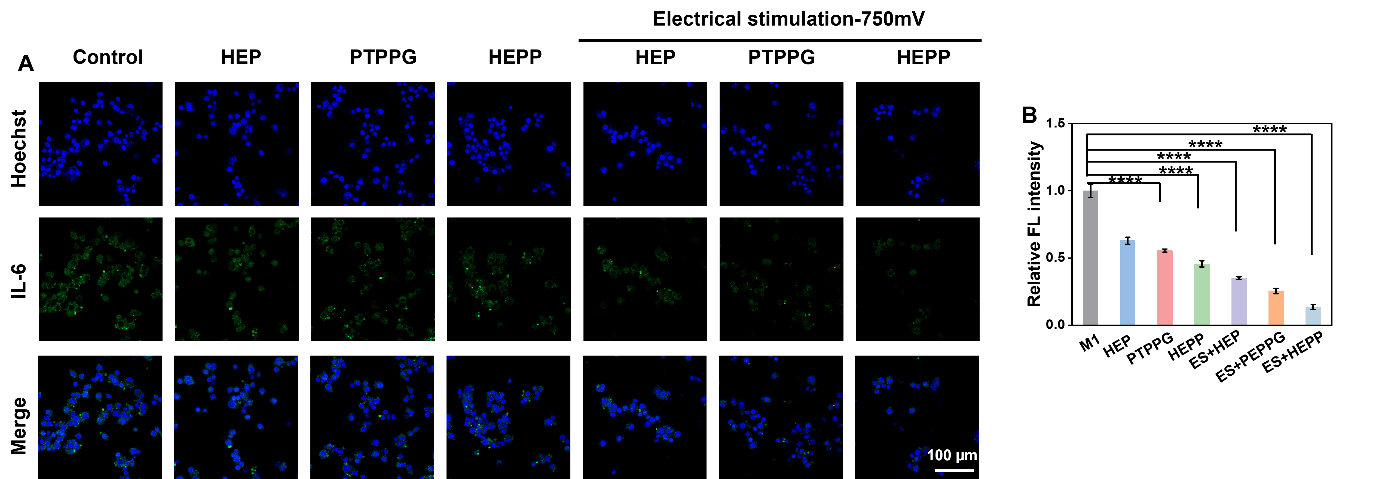


**Figure S53.** (A) Immunofluorescence staining of IL-6 expression in RAW264.7 cells under different stimulations. (B) Assessment of fluorescence relative intensities of IL-6 stains in RAW264.7 cells. Error bars represent the mean ± standard deviation for a sample size of 3. *p < 0.05, **p < 0.01, ***p < 0.001, ****p < 0.0001.


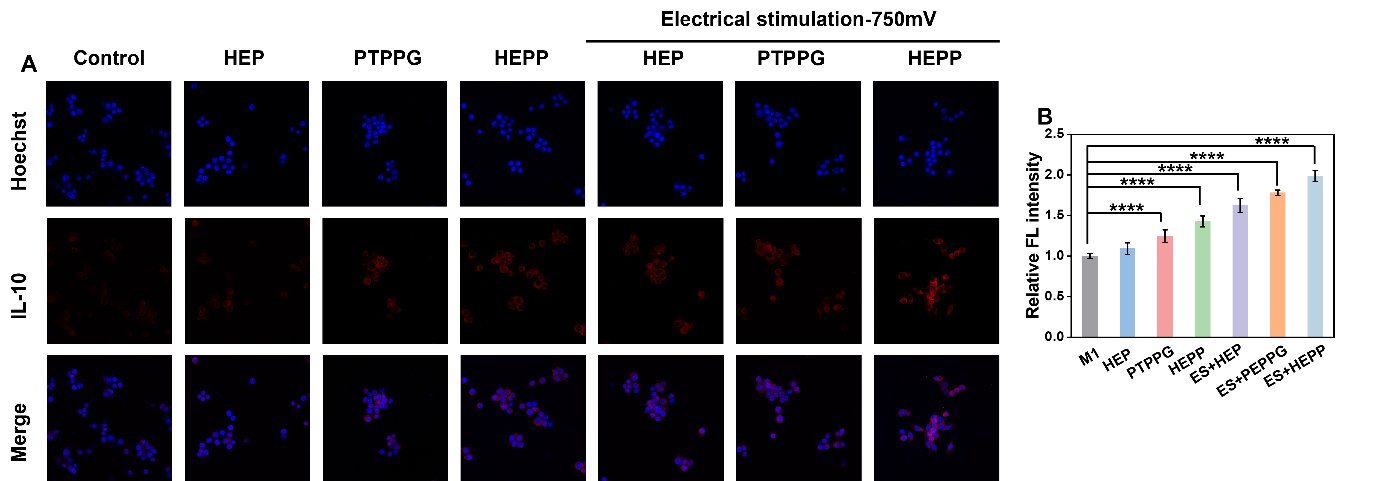


**Figure S54.** (A) Immunofluorescence staining of IL-10 expression in RAW264.7 cells under different stimulations. (B) Assessment of fluorescence relative intensities of IL-10 stains in RAW 264.7 cells. Error bars represent the mean ± standard deviation for a sample size of 3. *p < 0.05, **p < 0.01, ***p < 0.001, ****p < 0.0001.


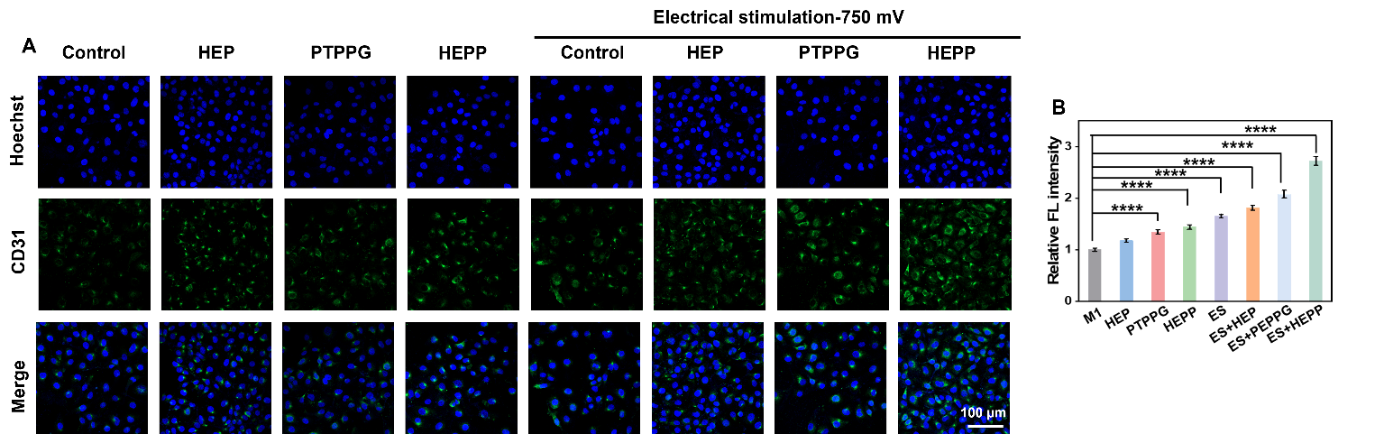


**Figure S55.** (A) Immunofluorescence staining of CD31 expression in cells under different stimulations. (B) Assessment of fluorescence relative intensities of CD31 stains in cells. Error bars represent the mean ± standard deviation for a sample size of 3. *p < 0.05, **p < 0.01, ***p < 0.001, ****p < 0.0001.


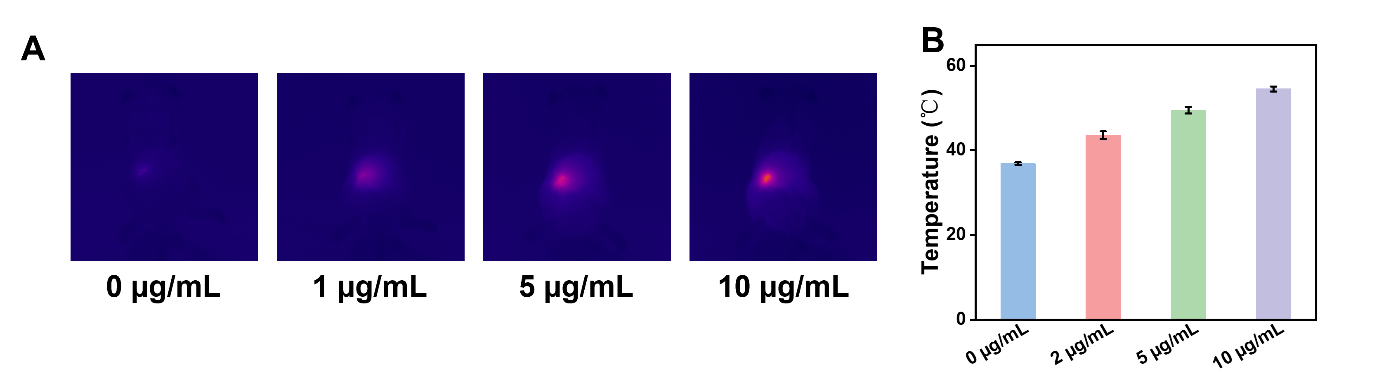


**Figure S56.** Photothermal images (A) and photothermal temperature changes (B) at the wound site incubated with different concentrations of PTPPG in vivo.


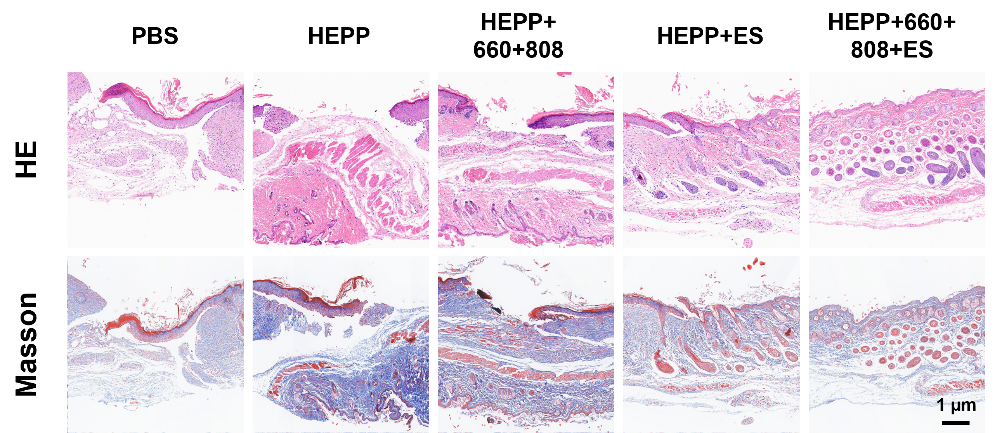


**Figure S57.** Enlarged images of the wound site from both H&E and Masson staining.


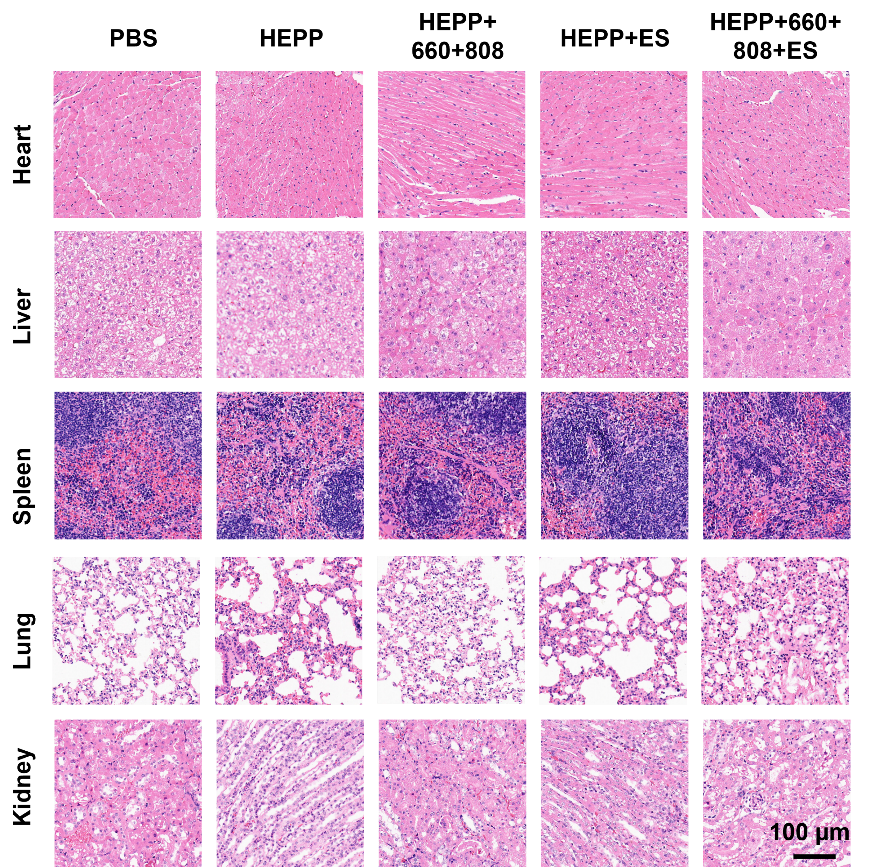


**Figure S58.** Photographs showing H&E strained principal organs from rats after different treatments.


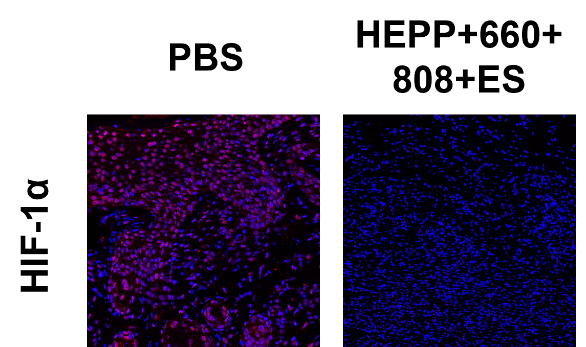


**Figure S59.** Immunofluorescence of HIF-1α in wound tissue sections of animals with different treatments.

**Table S1.** The primers used in the qRT-PCR analysis.

| TNF-α F (mouse) | CACGCTCTTCTGTCTACTGAACTTC |
| --- | --- |
| TNF-α R (mouse) | CTTGGTGGTTTGTGAGTGTGAGG |
| IL-6 F (mouse) | GAGAGGAGACTTCACAGAGGATACC |
| IL-6 R (mouse) | TCATTTCCACGATTTCCCAGAGAAC |
| IL-10 F (mouse) | GGACAACATACTGCTAACCGACTC |
| IL-10 R (mouse) | GGGCATCACTTCTACCAGGTAAAAC |
| TGF-β F (mouse) | TCTGCATTGCACTTATGCTGA |
| TGF-β R (mouse) | AAAGGGCGATCTAGTGATGGA |
| HIF-1α F (mouse) | GAACGTCGAAAAGAAAAGTCTCG |
| HIF-1α F (mouse) | CCTTATCAAGATGCGAACTCACA |
| Actin F (mouse) | ACTGCCGCATCCTCTTCCTC |
| Actin R (mouse) | AACCGCTCGTTGCCAATAGTG |
